# Supplementary material for: Sodium channel-inhibiting drugs and cancer-specific survival: a population-based study of electronic primary care data
Source: BMJ Open. 2023 Feb 3;13(2):e064376. doi: 10.1136/bmjopen-2022-064376 (PMC9900071; doi:10.1136/bmjopen-2022-064376)
Supplement: Supplementary data [file bmjopen-2022-064376supp001.pdf]

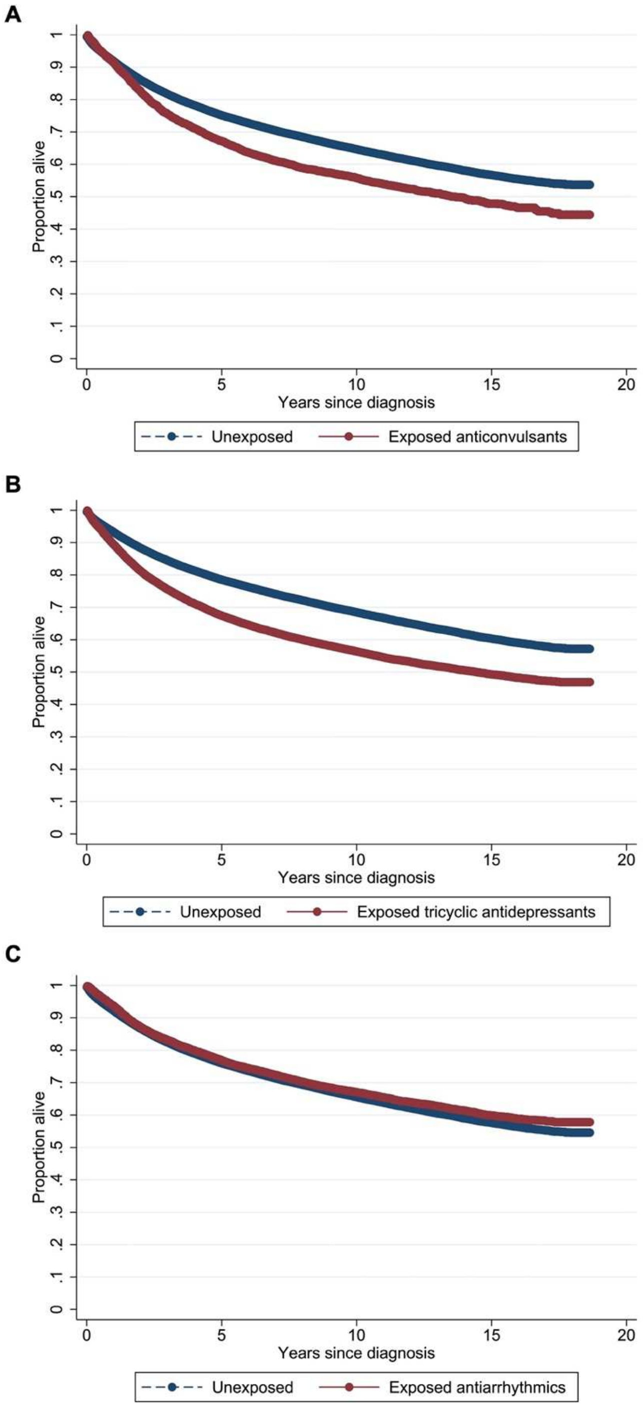

Supplementary Figure 1. Simon-Makuch survival curves for unexposed cancer patients and those ever exposed to VGSC-inhibiting anticonvulsant (A), tricyclic antidepressant (B) and antiarrhythmic (C) drugs in Scenario 3.

Fairhurst C, *et al. BMJ Open* 2023; 13:e064376. doi: 10.1136/bmjopen-2022-064376

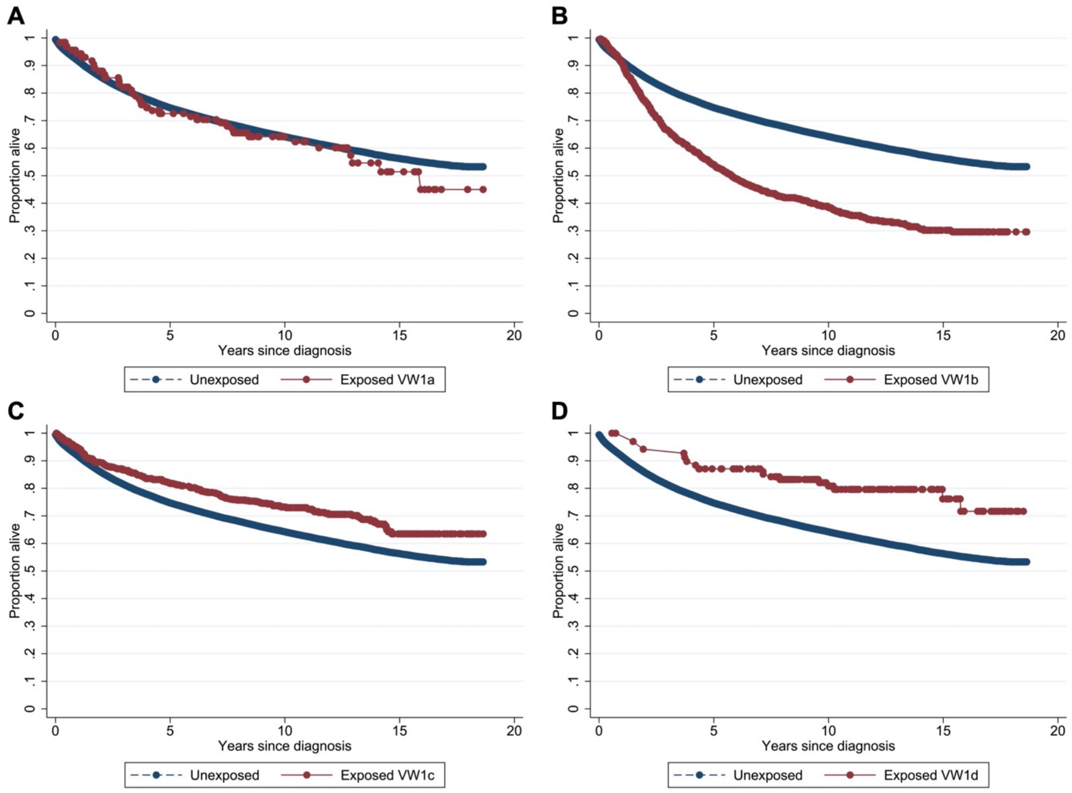

Supplementary Figure 2. Simon-Makuch survival curve for unexposed cancer patients and those ever exposed to Vaughan-Williams Class 1a (A), 1b (B), 1c (C) and 1d (D) drugs in Scenario 3.

Fairhurst C, *et al. BMJ Open* 2023; 13:e064376. doi: 10.1136/bmjopen-2022-064376

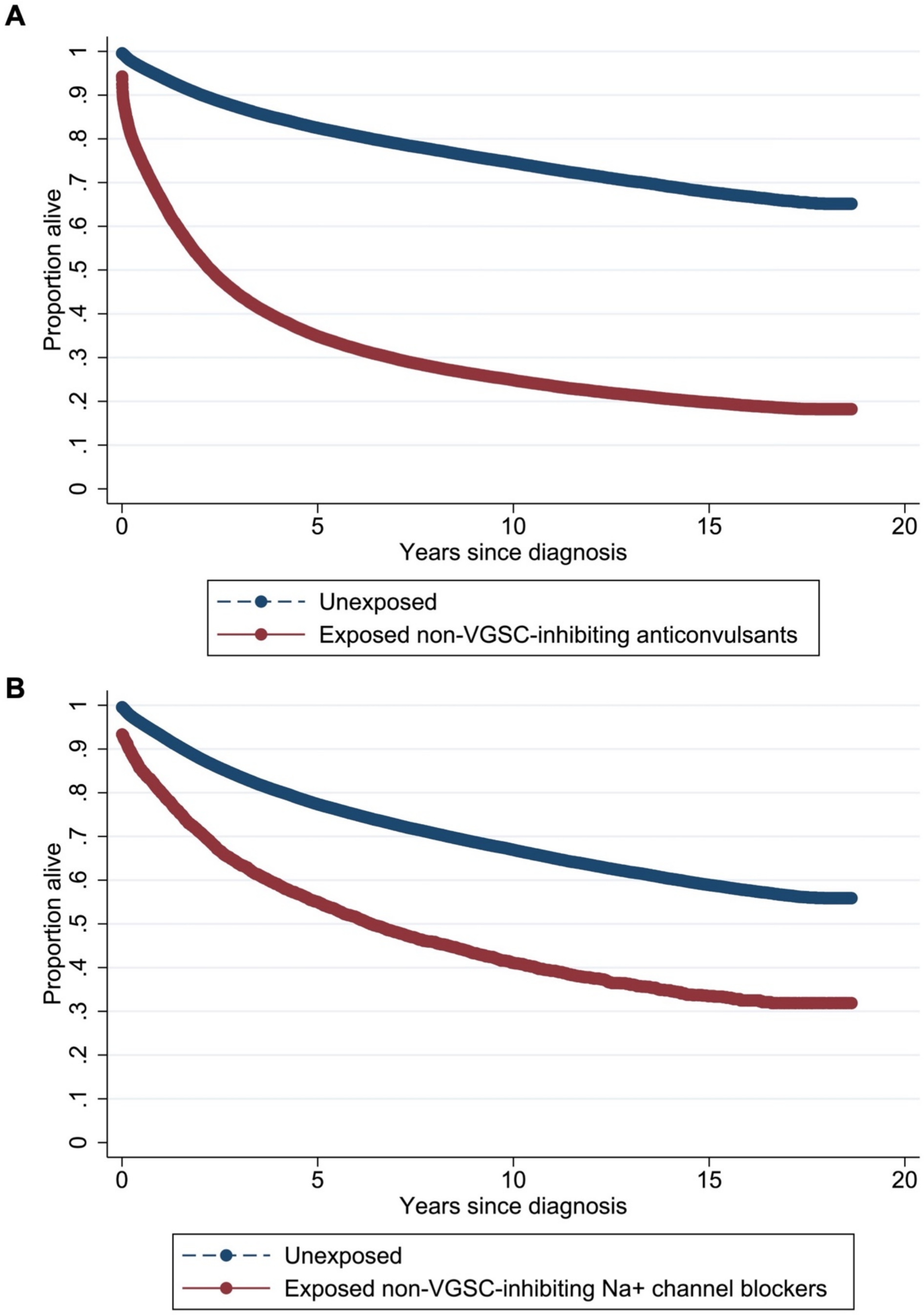

Supplementary Figure 3. Simon-Makuch survival curve for unexposed cancer patients and those ever exposed to non-VGSC-inhibiting anticonvulsants (A) and non-VGSC-inhibiting Na^+ channel blockers (B) in Scenario 3.

Fairhurst C, *et al. BMJ Open* 2023; 13:e064376. doi: 10.1136/bmjopen-2022-064376

Supplementary Table 1. Drug groups and classifications used in this study.

Drug	Classification	Vaughan Williams Classification[a]
**A. VGSC inhibitors**		
Articaine	Amide local anaesthetic	
Bupivacaine	Amide local anaesthetic	
Cinchocaine	Amide local anaesthetic	
Etidocaine	Amide local anaesthetic	
Levobupivacaine	Amide local anaesthetic	
Lidocaine	Amide local anaesthetic	1b
Mepivacaine	Amide local anaesthetic	
Prilocaine	Amide local anaesthetic	
Ropivacaine	Amide local anaesthetic	
Trimecaine	Amide local anaesthetic	
Ranolazine	Antiarrhythmic	1d
Ajmaline	Antiarrhythmic	1a
Amiodarone	Antiarrhythmic	3
Aprindine	Antiarrhythmic	1b
Disopyramide	Antiarrhythmic	1a
Dronedarone	Antiarrhythmic	3
Encainide	Antiarrhythmic	1c
Flecainide	Antiarrhythmic	1c
Mexiletine	Antiarrhythmic	1b
Moricizine/Moricizine hydrochloride	Antiarrhythmic	1c
Pilsicainide	Antiarrhythmic	1c
Procainamide	Antiarrhythmic	1a
Propafenone	Antiarrhythmic	1c
Quinidine	Antiarrhythmic	1a
Tocainide	Antiarrhythmic	1b

Fairhurst C, *et al. BMJ Open* 2023; 13:e064376. doi: 10.1136/bmjopen-2022-064376

Carvedilol	Antiarrhythmic	2
Labetalol	Antiarrhythmic	2
Oxprenolol	Antiarrhythmic	2
Propranolol	Antiarrhythmic	2
Esmolol	Antiarrhythmic	2
Carbamazepine	Anticonvulsant	
Eslicarbazepine	Anticonvulsant	
Eslicarbazepine acetate	Anticonvulsant	
Ethotoin	Anticonvulsant	
Fosphenytoin	Anticonvulsant	
Lacosamide	Anticonvulsant	
Lamotrigine	Anticonvulsant	
Oxcarbazepine	Anticonvulsant	
Phenytoin	Anticonvulsant	1b
Rufinamide	Anticonvulsant	
Sodium Valproate	Anticonvulsant	
Topiramate	Anticonvulsant	
Valproic acid	Anticonvulsant	
Zonisamide	Anticonvulsant	
Benzocaine	Ester local anaesthetic	
Procaine	Ester local anaesthetic	
Tetracaine	Ester local anaesthetic	
Riluzole	ALS treatment	
Amitriptyline	Tricyclic antidepressant	
Desipramine	Tricyclic antidepressant	
Duloxetine	Tricyclic antidepressant	
Fluoxetine	Tricyclic antidepressant	
Imipramine	Tricyclic antidepressant	
Maprotiline	Tricyclic antidepressant	

Fairhurst C, *et al. BMJ Open* 2023; 13:e064376. doi: 10.1136/bmjopen-2022-064376

Nortriptyline	Tricyclic antidepressant	
**A. Non-VGSC Na^+ channel blocker**		
Amiloride	ENaC inhibitor	
Triamterene	ENaC inhibitor	
**B. Non-VGSC-inhibiting anticonvulsants**		
Perampanel	AMPA receptor non-competitive antagonist	
Stiripentol	Aromatic allylic alcohol	
Phenobarbital	Barbiturate	
Primidone	Barbiturate	
Clobazam	Benzodiazepine	
Clonazepam	Benzodiazepine	
Diazepam	Benzodiazepine	
Lorazepam	Benzodiazepine	
Midazolam	Benzodiazepine	
Ethosuximide	Ca^{2+} channel inhibitor	
Gabapentin	Ca^{2+} channel inhibitor	
Pregabalin	Ca^{2+} channel inhibitor	
Acetazolamide	Carbonic anhydrase inhibitor	
Tiagabine	GABA reuptake inhibitor	
Vigabatrin	GABA reuptake inhibitor	
Brivaracetam	SV2A inhibitor	
Levetiracetam	SV2A inhibitor	

[a]According to (37,38).

Fairhurst C, *et al. BMJ Open* 2023; 13:e064376. doi: 10.1136/bmjopen-2022-064376

Supplementary Table 2. Characteristics of the participants stratified by exposure status.

	**A VGSC-inhibitor prescription (any at any time exc anaesthetics) (n=53724)**	**No VGSC-inhibitor prescriptions (n=79272)**	**Total (n=132996)**	**p-value**
**Sex, n (%)**				
Male	22531 (41.9)	41402 (52.2)	63933 (48.1)	<0.001
Female	31193 (58.1)	37870 (47.8)	69063 (51.9)	
**Age at diagnosis, years**				
Mean (SD)	65.9 (13.0)	68.0 (13.3)	67.1 (13.2)	<0.001
**Ethnicity, n (%)**				
White	50495 (94.0)	72056 (90.9)	122551 (92.1)	<0.001
Mixed/Multiple ethnic groups	161 (0.3)	299 (0.4)	460 (0.3)	
Asian/Asian British	801 (1.5)	1297 (1.6)	2098 (1.6)	
Black/Black British	733 (1.4)	1597 (2.0)	2330 (1.8)	
Other	190 (0.4)	336 (0.4)	526 (0.4)	

Not recorded/known	1344 (2.5)	3687 (4.7)	5031 (3.8)	
**Index of Multiple Deprivation, n (%)**				
Mean (SD)	9.2 (5.6)	9.0 (5.5)	9.1 (5.5)	<0.001
**Smoking status, n (%)**				
Heavy smoker	1596 (3.0)	1648 (2.1)	3244 (2.4)	<0.001
Moderate smoker	4503 (8.4)	5259 (6.6)	9762 (7.3)	
Light smoker	1675 (3.1)	2156 (2.7)	3831 (2.9)	
Ex-smoker	16413 (30.6)	23004 (29.0)	39417 (29.6)	
Non-smoker	28676 (53.4)	44072 (55.6)	72748 (54.7)	
Not recorded/known	861 (1.6)	3133 (4.0)	3994 (3.0)	
**Alcohol intake, n (%)**				
Heavy drinker	13656 (25.4)	20565 (25.9)	34221 (25.7)	<0.001
Moderate drinker	3421 (6.4)	5003 (6.3)	8424 (6.3)	
Light drinker	11829 (22.0)	16231 (20.5)	28060 (21.1)	

Non drinker	9001 (16.8)	10442 (13.2)	19443 (14.6)	
Not recorded/known	15817 (29.4)	27031 (34.1)	42848 (32.2)	
**BMI category, n (%)**				
Overweight/Obese	32166 (59.9)	42432 (53.5)	74598 (56.1)	<0.001
Normal range	15526 (28.9)	24137 (30.4)	39663 (29.8)	
Underweight	2345 (4.4)	3598 (4.5)	5943 (4.5)	
Not recorded/known	3687 (6.9)	9105 (11.5)	12792 (9.6)	
**Physical activity, n (%)**				
Very active	2634 (4.9)	4173 (5.3)	6807 (5.1)	<0.001
Moderately active	18614 (34.6)	25806 (32.6)	44420 (33.4)	
Inactive	7551 (14.1)	8179 (10.3)	15730 (11.8)	
Not recorded/known	24925 (46.4)	41114 (51.9)	66039 (49.7)	
**Type of cancer, n (%)**				
Breast	27106 (50.5)	32422 (40.9)	59528 (44.8)	<0.001

Fairhurst C, *et al. BMJ Open* 2023; 13:e064376. doi: 10.1136/bmjopen-2022-064376

Bowel	8435 (15.7)	14432 (18.2)	22867 (17.2)	
Prostate	18183 (33.8)	32418 (40.9)	50601 (38.0)	
**Total CCI score**				
Mean (SD)	6.1 (2.8)	5.9 (2.7)	6.0 (2.7)	<0.001
**VGSC-inhibitor indication[a], n (%)**				
Epilepsy	1915 (3.6)	449 (0.6)	2364 (1.8)	<0.001
Cardiac arrhythmia	9646 (18.0)	9791 (12.4)	19437 (14.6)	<0.001
Amyotrophic lateral sclerosis	0 (0.0)	0 (0.0)	0 (0.0)	-
Neuropathic pain/painful neuropathy	9860 (18.4)	7271 (9.2)	17131 (12.9)	<0.001
**≥1 of above, n (%)**	18744 (34.9)	16048 (20.2)	34792 (26.2)	<0.001

[a] not mutually exclusive

VGSC, voltage gated sodium channel; SD, standard deviation; BMI, body mass index; CCI, Charlson Comorbidity Index score.

Supplementary Table 3. Characteristics of the ‘ever’ exposed group stratified by timing of exposure relative to their cancer diagnosis.

	**Before only (n=14,157)**	**Before and after (n=17,264)**	**After only (n=22,303)**	**VGSC inhibitor prescription (any at any time excluding local anaesthetics) (n=53,724)**
**Sex, n (%)**				
Male	6049 (42.7)	6325 (36.6)	10157 (45.5)	22531 (41.9)
Female	8108 (57.3)	10939 (63.4)	12146 (54.5)	31193 (58.1)
**Age at diagnosis, years**				
Mean (SD)	68.6 (13.4)	65.7 (12.9)	64.3 (12.5)	65.9 (13.0)
**Ethnicity, n (%)**				
White	13189 (93.2)	16366 (94.8)	20940 (93.9)	50495 (94.0)
Mixed/Multiple ethnic groups	35 (0.2)	47 (0.3)	79 (0.4)	161 (0.3)
Asian/Asian British	172 (1.2)	252 (1.5)	377 (1.7)	801 (1.5)
Black/Black British	169 (1.2)	186 (1.1)	378 (1.7)	733 (1.4)
Other	48 (0.3)	54 (0.3)	88 (0.4)	190 (0.4)
Not recorded/known	544 (3.8)	359 (2.1)	441 (2.0)	1344 (2.5)

Fairhurst C, *et al. BMJ Open* 2023; 13:e064376. doi: 10.1136/bmjopen-2022-064376

**Index of Multiple Deprivation, n (%)**				
Mean (SD)	9.2 (5.5)	9.5 (5.7)	9.0 (5.5)	9.2 (5.6)
**Smoking status, n (%)**				
Heavy smoker	373 (2.6)	561 (3.2)	662 (3.0)	1596 (3.0)
Moderate smoker	1139 (8.0)	1554 (9.0)	1810 (8.1)	4503 (8.4)
Light smoker	437 (3.1)	520 (3.0)	718 (3.2)	1675 (3.1)
Ex-smoker	4336 (30.6)	5362 (31.1)	6715 (30.1)	16413 (30.6)
Non-smoker	7562 (53.4)	9020 (52.2)	12094 (54.2)	28676 (53.4)
Not recorded/known	310 (2.2)	247 (1.4)	304 (1.4)	861 (1.6)
**Alcohol intake, n (%)**				
Heavy drinker	3467 (24.5)	4030 (23.3)	6159 (27.6)	13656 (25.4)
Moderate drinker	929 (6.6)	1036 (6.0)	1456 (6.5)	3421 (6.4)
Light drinker	3153 (22.3)	3879 (22.5)	4797 (21.5)	11829 (22.0)
Non drinker	2436 (17.2)	3404 (19.7)	3161 (14.2)	9001 (16.8)
Not recorded/known	4172 (29.5)	4915 (28.5)	6730 (30.2)	15817 (29.4)
**BMI category, n (%)**				

Overweight/Obese	7990 (56.4)	10699 (62.0)	13477 (60.4)	32166 (59.9)
Normal range	4267 (30.1)	4734 (27.4)	6525 (29.3)	15526 (28.9)
Underweight	764 (5.4)	728 (4.2)	853 (3.8)	2345 (4.4)
Not recorded/known	1136 (8.0)	1103 (6.4)	1448 (6.5)	3687 (6.9)
**Physical activity, n (%)**				
Very active	615 (4.3)	752 (4.4)	1267 (5.7)	2634 (4.9)
Moderately active	4497 (31.8)	5896 (34.2)	8221 (36.9)	18614 (34.6)
Inactive	1785 (12.6)	2816 (16.3)	2950 (13.2)	7551 (14.1)
Not recorded/known	7260 (51.3)	7800 (45.2)	9865 (44.2)	24925 (46.4)
**Type of cancer, n (%)**				
Breast	6766 (47.8)	9642 (55.9)	10698 (48.0)	27106 (50.5)
Bowel	2531 (17.9)	2472 (14.3)	3432 (15.4)	8435 (15.7)
Prostate	4860 (34.3)	5150 (29.8)	8173 (36.6)	18183 (33.8)
**Total CCI score**				
Mean (SD)	6.3 (2.8)	6.1 (2.8)	5.9 (2.8)	6.1 (2.8)
**Diagnosis of a VGSC inhibitor indication[a], n (%)**				

Epilepsy	202 (1.4)	1271 (7.4)	442 (2.0)	1915 (3.6)
Cardiac arrhythmia	2611 (18.4)	3362 (19.5)	3673 (16.5)	9646 (18.0)
Amyotrophic lateral sclerosis	0 (0.0)	0 (0.0)	0 (0.0)	0 (0.0)
Neuropathic pain/painful neuropathy	2126 (15.0)	3790 (22.0)	3944 (17.7)	9860 (18.4)
**≥1 of above, n (%)**	4400 (31.1)	7199 (41.7)	7145 (32.0)	18744 (34.9)
**Died, n (%)**	7800 (55.1)	8119 (47.0)	10109 (45.3)	26028 (48.4)
**Most common prescription 1[a]**				
Tricyclic antidepressant	9312 (65.8)	11495 (66.6)	17793 (79.8)	38600 (71.8)
Antiarrhythmic	4023 (28.4)	3828 (22.2)	3181 (14.3)	11032 (20.5)
Anticonvulsant	822 (5.8)	1935 (11.2)	1305 (5.9)	4062 (7.6)
Treatment for ALS	0 (0.0)	6 (0.0)	24 (0.1)	30 (0.1)
**Most common prescription 2[b]**				
Tricyclic antidepressant	7887 (55.7)	10831 (62.7)	15187 (68.1)	33905 (63.1)
Antiarrhythmic	3604 (25.5)	3726 (21.6)	2881 (12.9)	10211 (19.0)
Amide local anaesthetic	1905 (13.5)	782 (4.5)	2951 (13.2)	5638 (10.5)

Anticonvulsant	708 (5.0)	1907 (11.0)	1191 (5.3)	3806 (7.1)
Ester local anaesthetic	53 (0.4)	12 (0.1)	71 (0.3)	136 (0.3)
Treatment for ALS	0 (0.0)	6 (0.0)	22 (0.1)	28 (0.1)
**Length of exposure (days)**				
Mean (SD)	2095.1 (2220.1)	4889.9 (2473.2)	1912.7 (2077.5)	2917.5 (2627.5)
< 6 months, n (%)	3239 (22.9)	40 (0.2)	4589 (20.6)	7868 (14.6)
≥ 6 months, n (%)	10918 (77.1)	17224 (99.8)	17714 (79.4)	45856 (85.4)
**Recent exposure[c], n (%)**	1017 (7.2)	8580 (49.7)	0 (0.0)	9597 (17.9)

[a] excluding local anaesthetics.

[b] including local anaesthetics.

[c] ≥2 prescriptions relating to one of the VGSC-inhibiting drugs within 2 years before the date of the cancer diagnosis, including at least one within 6 months before.

Supplementary Table 4. Deaths stratified by exposure to non-VGSC-inhibiting anticonvulsants and non-VGSC-inhibiting Na^{+} channel blockers.

	A VGSC-inhibitor prescription (any other than Amide or Ester local anaesthetics at any time) (n=53724)	No VGSC-inhibitor prescriptions (except Amide or Ester local anaesthetics) (n=79272)	A non-VGSC-inhibiting anticonvulsant prescription (any at any time) (n=46017)	No exposure to a non-VGSC-inhibiting anticonvulsant prescription (n=86979)	Non-VGSC-inhibiting Na^{+} channel blocker prescription (any at any time) (n=9256)	No exposure to a non-VGSC-inhibiting Na^{+} channel blocker prescription (n=123740)
Died (any cause)	26028 (48.4)	40932 (51.6)	25284 (54.9)	41676 (47.9)	6969 (75.3)	59991 (48.5)
Died with any cancer as underlying cause	15933 (29.7)	26104 (32.9)	17987 (39.1)	24050 (27.7)	3601 (38.9)	38436 (31.1)
Died with any cancer as contributory cause	18598 (34.6)	30492 (38.5)	20003 (43.5)	29087 (33.4)	4540 (49.0)	44550 (36.0)
Died with index cancer as underlying cause	12282 (22.9)	20443 (25.8)	13925 (30.3)	18800 (21.6)	2842 (30.7)	29883 (24.1)

Fairhurst C, *et al. BMJ Open* 2023; 13:e064376. doi: 10.1136/bmjopen-2022-064376

Died with index cancer as contributory cause	15256 (28.4)	25482 (32.1)	16451 (35.7)	24287 (27.9)	3834 (41.4)	36904 (29.8)

Supplementary Table 5. Estimates of the relationship between exposure to non-VGSC-inhibiting drugs, subdivided by type, and cancer-specific mortality (empty cells indicate that analysis was not permitted due to low numbers).

**Non-VGSC-inhibiting anticonvulsants drug groups**	**Exposed* (n=46017), n (%)**	**HR (95% CI) p-value Scenario 1**	**HR (95% CI) p-value Scenario 2**	**HR (95% CI) p-value Scenario 3**
Ever use				
AMPA receptor non-competitive antagonist	4 (0.1)	-	-	-
Aromatic allylic alcohol	0 (0.0)	-	-	-
Barbiturate	469 (1.0)	1.39 (1.19, 1.63) p<0.001	1.28 (1.07, 1.52) p=0.01	1.42 (1.19, 1.69) p<0.001
Benzodiazepine	37696 (81.9)	3.01 (2.95, 3.07) p<0.001	3.73 (3.65, 3.81) p<0.001	4.91 (4.80, 5.02) p<0.001
Calcium channel inhibitor	11643 (21.7)	2.19 (2.12, 2.26) p<0.001	2.27 (2.19, 2.34) p<0.001	2.80 (2.71, 2.90) p<0.001
Carbonic anhydrase inhibitor	14274 (31.0)	0.92 (0.79, 1.06) p=0.25	0.93 (0.76, 1.13) p=0.46	1.25 (1.02, 1.53) p=0.03
GABA reuptake inhibitor	23 (0.1)	-	-	-
SV2A inhibitor	620 (1.4)	2.98 (2.63, 3.38) p<0.001	3.03 (2.67, 3.44) p<0.001	3.75 (3.30, 4.25) p<0.001
Recent use				

AMPA receptor non-competitive antagonist	0 (0.0)	-	-	-
Aromatic allylic alcohol	0 (0.0)	-	-	-
Barbiturate	208 (0.5)	1.44 (1.16, 1.78) p<0.001	1.37 (1.10, 1.71) p<0.001	1.46 (1.18, 1.82) p<0.001
Benzodiazepine	2686 (5.8)	1.46 (1.37, 1.56) p<0.001	1.34 (1.25, 1.44) p<0.001	1.51 (1.41, 1.62) p<0.001
Calcium channel inhibitor	902 (2.0)	1.12 (0.99, 1.26) p=0.08	1.06 (0.93, 1.21) p=0.38	1.20 (1.05, 1.37) p=0.01
Carbonic anhydrase inhibitor	43 (0.1)	-	-	-
GABA reuptake inhibitor	0 (0.0)	-	-	-
SV2A inhibitor	54 (0.1)	-	-	-
Most common VGSC-inhibitor prescription				
AMPA receptor non-competitive antagonist	0 (0.0)	-	-	-
Aromatic allylic alcohol	0 (0.0)	-	-	-
Barbiturate	348 (0.8)	1.43 (1.20, 1.71) p<0.001	1.30 (1.07, 1.59) p=0.01	1.40 (1.15, 1.71) p<0.001

Fairhurst C, *et al. BMJ Open* 2023; 13:e064376. doi: 10.1136/bmjopen-2022-064376

Benzodiazepine	32563 (70.8)	3.06 (2.99, 3.12) p<0.001	4.02 (3.93, 4.11) p<0.001	5.11 (4.99, 5.23) p<0.001
Calcium channel inhibitor	12061 (26.2)	2.22 (2.15, 2.30) p<0.001	2.33 (2.24, 2.41) p<0.001	2.80 (2.70, 2.90) p<0.001
Carbonic anhydrase inhibitor	561 (1.2)	0.92 (0.78, 1.08) p=0.31	0.92 (0.72, 1.18) p=0.52	1.22 (0.96, 1.56) p=0.11
GABA reuptake inhibitor	7 (0.0)	-	-	-
SV2A inhibitor	477 (1.0)	3.16 (2.73, 3.65) p<0.001	3.23 (2.79, 3.74) p<0.001	4.00 (3.45, 4.63) p<0.001

*Figures in this column relate to the number of patients recorded as having at least some follow-up time considered as exposed to the drug group of interest in Scenario 1 for each definition (ever use, recent use, most common), as a percentage of the whole ‘ever’ exposed group. The number of patients with any person-time of follow-up considered as exposed for each drug group will be lower in Scenario 2, and fewer still in Scenario 3.
