# Supplementary material for: Sodium channel-inhibiting drugs and cancer-specific survival: a population-based study of electronic primary care data
Source: BMJ Open. 2023 Feb 3;13(2):e064376. doi: 10.1136/bmjopen-2022-064376 (PMC9900071; doi:10.1136/bmjopen-2022-064376)
Supplement: Author's manuscript [file bmjopen-2022-064376.draft_revisions.pdf]

BMJ Open

BMJ Open is committed to open peer review. As part of this commitment we make the peer review history of every article we publish publicly available.

When an article is published we post the peer reviewers' comments and the authors' responses online. We also post the versions of the paper that were used during peer review. These are the versions that the peer review comments apply to.

The versions of the paper that follow are the versions that were submitted during the peer review process. They are not the versions of record or the final published versions. They should not be cited or distributed as the published version of this manuscript.

BMJ Open is an open access journal and the full, final, typeset and author-corrected version of record of the manuscript is available on our site with no access controls, subscription charges or pay-per-view fees (http://bmjopen.bmj.com).

If you have any questions on BMJ Open's open peer review process please email info.bmjopen@bmj.com

BMJ Open

Sodium channel-inhibiting drugs and cancer-specific survival: a population-based study using the CPRD

Journal:	*BMJ Open*
Manuscript ID	bmjopen-2022-064376
Article Type:	Original research
Date Submitted by the Author:	04-May-2022

Complete List of Authors:	Fairhurst, Caroline; University of York, Health Sciences<br>Martin, Fabiola; University of Queensland<br>Watt, Ian; University of York, Health Sciences<br>Bland, Martin; University of York, Health Sciences<br>Doran, Tim; University of York, Health Sciences<br>Brackenbury, William; University of York, Department of Biology

Keywords:	Epilepsy < NEUROLOGY, ONCOLOGY, Breast tumours < ONCOLOGY

SCHOLARONE™
Manuscripts

I, the Submitting Author has the right to grant and does grant on behalf of all authors of the Work (as defined in the below author licence), an exclusive licence and/or a non-exclusive licence for contributions from authors who are: i) UK Crown employees; ii) where BMJ has agreed a CC-BY licence shall apply, and/or iii) in accordance with the terms applicable for US Federal Government officers or employees acting as part of their official duties; on a worldwide, perpetual, irrevocable, royalty-free basis to BMJ Publishing Group Ltd ("BMJ") its licensees and where the relevant Journal is co-owned by BMJ to the co-owners of the Journal, to publish the Work in this journal and any other BMJ products and to exploit all rights, as set out in our licence.

The Submitting Author accepts and understands that any supply made under these terms is made by BMJ to the Submitting Author unless you are acting as an employee on behalf of your employer or a postgraduate student of an affiliated institution which is paying any applicable article publishing charge ("APC") for Open Access articles. Where the Submitting Author wishes to make the Work available on an Open Access basis (and intends to pay the relevant APC), the terms of reuse of such Open Access shall be governed by a Creative Commons licence – details of these licences and which Creative Commons licence will apply to this Work are set out in our licence referred to above.

Other than as permitted in any relevant BMJ Author's Self Archiving Policies, I confirm this Work has not been accepted for publication elsewhere, is not being considered for publication elsewhere and does not duplicate material already published. I confirm all authors consent to publication of this Work and authorise the granting of this licence.

For peer review only

Sodium channel-inhibiting drugs and cancer-specific survival: a population-based study using the CPRD

Caroline Fairhurst[1], Fabiola Martin[2], Ian Watt[1], Martin Bland[1], Tim Doran[1], William J. Brackenbury[3] (0000-0001-6882-3351)

[1]Department of Health Sciences, University of York, UK

[2]School of Public Health, University of Queensland, Australia

[3]York Biomedical Research Institute, Department of Biology, University of York, UK

*Corresponding author:

William J. Brackenbury, York Biomedical Research Institute, Department of Biology, University of York, Heslington, York, YO10 5DD, UK

E-mail: william.brackenbury@york.ac.uk

Telephone: +44 (0) 1904 328 284

Word count: 4518

Keywords: Anticonvulsants / Cancer / Epilepsy / Sodium channels

For peer review only

ABSTRACT

Objectives: Antiepileptic and antiarrhythmic drugs inhibit voltage-gated sodium (Na^+) channels (VGSCs), and preclinical studies show that these medications reduce tumour growth, invasion and metastasis. We investigated the association between VGSC inhibitor use and survival in breast, bowel and prostate cancer patients.

Design: Retrospective cohort study.

Setting: Individual primary care records extracted from the CPRD database.

Participants: Records for 132,996 patients with a diagnosis of breast, bowel or prostate cancer.

Primary and secondary outcome measures: Adjusted Cox proportional hazards regression was used to analyse cancer-specific survival associated with exposure to VGSC inhibitors. Exposure to non-VGSC-inhibiting antiepileptic medication and other non-VGSC blockers were also considered. Drug exposure was treated as a time-varying covariate to account for immortal time bias.

Results: During 1,002,225 person-years of follow-up, there were 42,037 cancer-specific deaths. 53,724 (40.4%) cancer patients had at least one prescription for a VGSC inhibitor of interest. Increased risk of cancer-specific mortality was associated with exposure to this group of drugs (hazard ratio: 1.59, 95% CI 1.56 to 1.63, p<0.001). This risk applied to VGSC-inhibiting tricyclic antidepressants, local anaesthetics and anticonvulsants, and persisted in sensitivity analyses stratifying by index cancer diagnosis and for other mortality outcomes. In contrast, exposure to VGSC-inhibiting Class 1c and 1d antiarrhythmics was associated with significantly improved cancer-specific survival in exploratory analyses.

Conclusions: Association between VGSC inhibitor use and mortality in cancer patients varies according to indication. Exposure to VGSC-inhibiting antiarrhythmics, but not anticonvulsants, supports findings from preclinical data, with improved survival. However,

For peer review only - http://bmjopen.bmj.com/site/about/guidelines.xhtml

additional confounding factors may underlie these associations, highlighting the need for further study.

Strengths and limitations of this study

- Primary care research data with large sample size and statistical power.
- No direct information on metastasis as an outcome.
- Drug exposure data are based on prescriptions.
- Drug exposure is treated as a time-varying covariate to account for immortal time bias.

For peer review only

INTRODUCTION

Metastatic disease is the leading cause of death from solid tumours (1), and there is an enduring need to identify new antimetastatic targets and therapies (2). One approach is to repurpose existing drugs used in the management of other conditions. In particular, ion channel blockers have been proposed as novel agents to treat cancer, including metastatic disease (3). However, no such agent has yet been progressed through to clinical use.

Voltage-gated sodium (Na^+) channels (VGSCs) are expressed on electrically excitable cells including neurons and muscle cells, where they regulate action potential firing (4). VGSC-inhibiting drugs are prescribed for a range of excitability-related conditions, including epilepsy, pain and cardiac arrhythmia (5,6). VGSCs are also widely expressed on malignant cells from a range of cancers, where they regulate Na^+ handling, pH buffering and the plasma membrane potential, promoting proliferation, migration, invasion and metastasis (7–12). Numerous preclinical studies have shown that VGSC-inhibiting medications can reduce tumour growth, invasion and metastasis (13–21). Although some antiepileptic drugs have been tested in clinical trials (22,23), their effect on VGSC activity in patient tumours has not been investigated. Several observational cohort studies have shown reduced cancer incidence (24,25) and risk of recurrence (26–28) in patients prescribed VGSC-inhibiting medications. In contrast, we have previously reported that exposure to VGSC-inhibiting medication was associated with reduced overall survival in cancer patients in a retrospective analysis (29). However, we were unable to control for epilepsy diagnosis, which is independently associated with increased all-cause mortality (30). In the present study, we conducted a retrospective cohort study using primary care data from the Clinical Practice Research Datalink (CPRD) in order to test the hypothesis that exposure to VGSC inhibitors prolongs cancer-specific survival. We controlled for epilepsy diagnosis and timing of exposure to VGSC-inhibiting and considered other antiepileptic medications.

For peer review only - http://bmjopen.bmj.com/site/about/guidelines.xhtml

METHODS

Patient data

The study protocol has been published previously (31). Several additional analyses were performed as detailed below. Primary care records for patients with a first diagnosis of any cancer between 2001 and 2011 and aged 25 years or over at diagnosis were obtained from the CPRD GOLD and Aurum databases. CPRD contains anonymised individual patient data on morbidity, mortality, prescribing, treatment and referrals collected from primary care practices in England. Data were extracted in August 2019. Within this dataset, we identified patients with a recorded medical code for breast, bowel or prostate cancer (hereafter referred to as the *index* cancers). The role of VGSCs has been extensively studied in these three types of cancer, and they are among the most common (12,31). Prescription data were interrogated to identify patients with a recorded prescription for VGSC-inhibiting medications (including anticonvulsants, local anaesthetics, antiarrhythmics and certain tricyclic antidepressants; Supplementary Table 1). We also identified patients with a recorded prescription for non-VGSC-inhibiting anticonvulsants (e.g. gabapentinoids, benzodiazepines) and medications targeting other (non-voltage-gated) Na^+ channels (e.g. the epithelial Na^+ channel [ENaC]), at any time (Supplementary Table 1). We searched diagnostic codes to identify patients with a recorded VGSC inhibitor indication (epilepsy, cardiac arrhythmia, amyotrophic lateral sclerosis (ALS), and neuropathic pain) (5,6).

Statistical analysis

Time-dependent Cox proportional hazards regression was used to analyse survival time from cancer diagnosis associated with exposure to the medication group of interest, and all models were adjusted for type of index cancer, sex and age at diagnosis (age+age^2), unless otherwise stated. Right censoring occurred if the patient died of any other cause, or was still alive at the point the data were extracted or the patient transferred out of a CPRD GP practice.

For peer review only

To account for potential immortal time bias (32) in patients whose prescriptions only begin after their cancer diagnosis, drug exposure status was considered as a time-dependent covariate in the following three ways:

Scenario 1: all person-time of follow-up from diagnosis to death/censor was classified as exposed for patients who have at least one prescription of interest before their diagnosis; while for those who only have prescriptions after their diagnosis, their survival time was classified as unexposed between diagnosis and date of first prescription, and as exposed thereafter.

Scenario 2: person-time was considered as unexposed until the date of the first prescription and as exposed thereafter for patients whose prescriptions either: (i) start before diagnosis and extend after; or (ii) start after diagnosis. This differs from Scenario 1 in that, for patients whose first and last prescriptions are before their cancer diagnosis, their survival time is treated as exposed in Scenario 1 and unexposed in this scenario.

Scenario 3: person-time was considered as unexposed until the date of the first prescription *following* the date of cancer diagnosis and as exposed thereafter. This differs from Scenario 2 in that, for patients whose prescriptions of interest start before diagnosis and extend after, the time between diagnosis and the first prescription after diagnosis is considered exposed in Scenario 2 and unexposed in this scenario.

In all scenarios, all person-time of follow-up for patients who have never had a recorded prescription of interest was classified as unexposed. A depiction of these scenarios is presented in the published protocol (31).

Multivariable-adjusted hazard ratios (HR) are presented with a 95% confidence interval (CI) and p value. Analyses were conducted in Stata v15 (33), using two-sided statistical tests at the 5% significance level.

For peer review only - http://bmjopen.bmj.com/site/about/guidelines.xhtml

Survival graphs were produced using the Simon–Makuch method, which is an alternative to Kaplan–Meier that appropriately accounts for the time-varying covariate of exposure (34).

Patient characteristics

Patient characteristics, stratified by 'ever' and 'never' exposure to a VGSC-inhibiting medication, are summarised using mean and standard deviation (SD) for continuous data and count and percentage for categorical variables, and compared using a t- or χ^2-test as appropriate. Amide and ester local anaesthetic injections were not included within the definition of 'exposed', due to their short-term use and transient effect.

Characteristics of the 'ever' exposed group stratified by timing of drug exposure relative to their cancer diagnosis (before only, before and after, and after only) are also presented, including length of drug exposure and most commonly prescribed drug class. Extent of drug exposure was estimated by calculating the time between the first and last recorded prescription, plus a number of weeks (the average interval between all prescriptions) to account for the time patients were assumed to be taking their final recorded prescription. Based on this, patients were classified into short (< 6 months), or long (≥ 6 months) exposure groups. Those who had two or more prescriptions relating to one of the VGSC-inhibiting drugs within two years before the date of the cancer diagnosis, including at least one within six months before, were classified as having recent (to cancer diagnosis) exposure. Alternative medications were summarised for patients with a recorded diagnostic code for an indication for a VGSC-inhibiting drug (epilepsy, neuropathic pain, cardiac arrhythmia, ALS) who did not have a recorded prescription for a VGSC-inhibiting drug.

Primary analysis

The primary analysis investigated cancer-specific mortality (any cancer as the underlying cause) associated with drug exposure, treated as a time-varying covariate according to the

For peer review only - http://bmjopen.bmj.com/site/about/guidelines.xhtml

three scenarios described above, using adjusted Cox proportional hazards regression models.

Sensitivity analyses

The primary analyses were repeated with the Cox models additionally adjusted for: ethnicity, BMI, physical activity, smoking status, alcohol consumption, Charlson comorbidity index (CCI) score, presence of an indication for VGSC-inhibiting medication and area-level social deprivation, using the Index of Multiple Deprivation (IMD) in twentiles (1=least deprived to 20=most deprived) based on patient postcode (2010). In further sensitivity analyses, missing values for the confounding factors (previously included in a 'not recorded' category) were imputed using multiple imputation and the analysis models rerun.

In addition, the primary analysis was repeated using competing-risks regression, according to the method of Fine and Gray and implemented using the *stcrreg* command in Stata, (35) with death by any other cause but cancer as the competing risk, and also after introducing a lag such that patients were not considered as exposed until three months after drug use. This excludes prescriptions shortly before death and therefore minimises potential reverse causation (36).

Secondary analyses

The primary analyses were repeated stratified by index cancer diagnosis (not adjusting for index cancer, and also removing sex as a covariate for prostate cancer analysis as all patients were male, and for breast cancer as nearly all patients were female), and comparing time to: i) death from index cancer (underlying or contributory cause); ii) death from any cancer (underlying or contributory cause); and iii) all-cause mortality. The primary analyses were also repeated by including in the 'ever' exposed group, in turn, only patients who had: i) ever used; ii) had recent exposure (according to definition as above) to; or iii) whose most commonly prescribed VGSC inhibitor (not including local anaesthetic injections) was tricyclic

For peer review only - http://bmjopen.bmj.com/site/about/guidelines.xhtml

antidepressants, anticonvulsants, and antiarrhythmics. Other drug classes were not considered due to insufficient numbers in these groups.

Vaughan Williams classification of antiarrhythmics

We repeated the primary analyses considering exposure to VGSC-inhibiting antiarrhythmics subdivided according to the updated Vaughan Williams classification (Supplementary Table 1) (37,38). Exposure to different classes of antiarrhythmic medications (Supplementary Table 1) was assessed depending on whether the patient's use of the drugs was defined as: (i) ever use; (ii) recent use; or (iii) their most common VGSC inhibitor prescription.

Amide or ester local anaesthetic injections

We repeated the time-dependent analysis (Scenario 3 only since 1 and 2 are not applicable here) including only those patients whose VGSC-inhibiting drug prescriptions were solely for amide or ester local anaesthetic injections (Supplementary Table 1) following their diagnosis in the exposed group, since there is evidence that local anaesthetics used perioperatively can be associated with reduced tumour recurrence (12).

Non-VGSC-targeting antiepileptic medication

We repeated the primary analyses considering exposure to non-VGSC-targeting antiepileptic medications, and blockers of other (non voltage-gated) Na^+ channels (Supplementary Table 1).

Ethics approval

This study was performed following ethics approval from the Department of Biology Ethics Committee, University of York (WB201909).

Patient and public involvement

No patient involved.

For peer review only - http://bmjopen.bmj.com/site/about/guidelines.xhtml

RESULTS

Population characteristics

The CPRD dataset contained records for 515,987 patients from 1,057 GP practices, including 132,996 (25.8%) patients with a diagnostic code for breast (n=59,528), prostate (n=50,601) or bowel (n=22,867) cancer recorded during at least one of their GP consultations. Of the 132,996 index cancer patients, 79,164 (59.5%) had at least one prescription, at any time, for a specified VGSC-inhibiting drug; tricyclic antidepressant was the most commonly prescribed VGSC-inhibiting drug group for the majority of exposed patients (n=33,905, 42.8%), followed by amide local anaesthetics (n=30,091, 38.0%). For a third of these 79,164 patients (n=25,440, 32.1%), their only exposure to a VGSC-inhibiting drug was to amide or ester local anaesthetics. These patients were classified as unexposed for most of the described analyses, due to the short-term exposure, so 53,724 (40.4%) patients were observed to have had at least some exposure to a VGSC inhibitor of interest, before and/or after cancer diagnosis, and 79,272 (59.6%) were not (Supplementary Table 2). Stratified by index cancer, the proportion of 'ever' exposed patients was: breast 59.5%; bowel 54.7%; and prostate 61.7%.

Between the 'ever' and 'never' exposure groups, formal comparisons indicated statistically significant differences in all observed characteristics, even where differences were very small such as in the Charlson Comorbidity Index (mean 6.1 in the 'ever' exposed group and 5.9 in the unexposed group), which is likely to be an artefact of the large sample size (Supplementary Table 2). On visual inspection, the two exposure groups appear similar for most patient characteristics, including age, but there was a notable imbalance in sex, with a greater proportion of females in the 'ever' exposed group than in the unexposed group. There were expected differences in the proportions of patients with an indication for treatment with a VGSC inhibitor; for example, 3.6% of the 'ever' exposed group had a diagnosis of epilepsy, compared with 0.6% of the unexposed group.

For peer review only - http://bmjopen.bmj.com/site/about/guidelines.xhtml

Within the 'ever' exposed group, 14,157 patients (26.4%) only had prescriptions of interest dated before a cancer diagnosis, 17,264 (32.1%) had prescriptions dated both before and after diagnosis, and 22,303 (41.5%) only had prescriptions dated after diagnosis (Supplementary Table 3). For patients who initiated VGSC inhibitors after their cancer diagnosis, the mean interval between diagnosis and first recorded prescription was 4.0 years (SD 3.5, median 3.0, range 1 day to 18.2 years).

For the subset of patients with a recorded diagnosis of an indication for VGSC-inhibiting medication in their medical records who did not have a recorded prescription for a VGSC-inhibiting drug (n=16,048), the most common prescriptions were for angiotensin-converting enzyme inhibitors (727,736 prescriptions among 9,887 (61.6%) patients), lipid-regulating drugs (647,200 prescriptions among 8,099 (50.5% patients), antiplatelet drugs (622,772 prescriptions among 10,602 (66.1%) patients), beta-adrenoceptor blocking drugs (515,888 prescriptions among 7,798 (48.6%) patients) and voltage-gated calcium channel blockers (503,847 prescriptions among 8,044 (50.1%) patients). These proportions were very similar for the subset of patients with a recorded diagnosis of an indication for VGSC-inhibiting medication in their medical records who did have a recorded prescription for a VGSC-inhibiting drug (n=18,744), except that a slightly higher proportion of these patients had a prescription for a beta-blocker (54.9%).

The maximum follow-up from diagnosis was 18.6 years (median 7.9 years). During 1,002,225 person-years of follow-up, there were 66,960 deaths from any cause (Supplementary Table 4). A similar proportion of deaths from any cause were recorded in the data for the two groups ('ever' exposed 48.4%, unexposed 51.6%), and of deaths with any cancer listed as the underlying cause (primary outcome, total n=42,037; 'ever' exposed 29.7%, unexposed 32.9%) or as at least a contributory cause (n=32,725; 'ever' exposed 34.6%, unexposed 38.5%) (Supplementary Table 4).

Primary, sensitivity and secondary analyses

For peer review only - http://bmjopen.bmj.com/site/about/guidelines.xhtml

The main text focuses on results from analyses relating to Scenario 3, as this most closely matches the design of relevant preclinical studies (13,14), but all results are presented in the tables. In the primary analysis, we considered the relationship between all VGSC inhibitors (excluding local anaesthetics) and cancer-specific survival across all three index cancer types (breast, bowel and prostate) combined. Exposure to VGSC inhibitors was associated with a statistically significant increased risk of death from cancer (HR 1.59, 95% CI 1.56 to 1.63, p<0.001; Table 1; Figure 1). The HR increased in the sensitivity analysis additionally adjusted for ethnicity, BMI, physical activity, smoking status, alcohol consumption, IMD, CCI score, and presence of a VGSC-inhibitor indication (1.65, 95% CI 1.62 to 1.69), and in the competing-risks analysis (1.65, 95% CI 1.53 to 1.78), but was similar after missing covariate data were imputed albeit with a wider confidence interval (1.60, 95% CI 1.49 to 1.72). A smaller but still significant effect was observed in the analysis that utilised a lag of three months to discount drug use shortly before death (HR 1.37, 95% CI 1.34 to 1.41, p<0.001; Table 1).

For peer review only

Table 1. Estimates of the relationship between exposure to VGSC inhibitors and cancer specific mortality - primary and sensitivity analyses.

Cancer-specific mortality (underlying cause)	HR (95% CI)	p-value
**Primary analysis**		
Scenario 1	1.33 (1.31, 1.36)	<0.001
Scenario 2	1.31 (1.28, 1.34)	<0.001
Scenario 3	1.59 (1.56, 1.63)	<0.001
**Sensitivity analyses 1[a]**		
Scenario 1	1.42 (1.39, 1.45)	<0.001
Scenario 2	1.38 (1.34, 1.41)	<0.001
Scenario 3	1.65 (1.62, 1.69)	<0.001
**Sensitivity analyses 2[b]**		
Scenario 1	1.34 (1.26, 1.43)	<0.001
Scenario 2	1.31 (1.22, 1.41)	<0.001
Scenario 3	1.60 (1.49, 1.72)	<0.001
**Sensitivity analyses 3[c]**		
Scenario 1	1.34 (1.26, 1.43)	<0.001
Scenario 2	1.35 (1.25, 1.45)	<0.001
Scenario 3	1.65 (1.53, 1.78)	<0.001
**Sensitivity analyses 4[d]**		
Scenario 1	1.20 (1.18, 1.23)	<0.001
Scenario 2	1.17 (1.14, 1.20)	<0.001
Scenario 3	1.37 (1.34, 1.41)	<0.001

[a]primary analyses additionally adjusted for ethnicity, BMI, physical activity, smoking status, alcohol consumption, CCI score, IMD score and presence of: epilepsy; cardiac arrhythmias; ALS; neuropathic pain/painful neuropathy.
[b]sensitivity analyses 1 repeated after unknown values of ethnicity, BMI, physical activity, smoking status, IMD and alcohol consumption imputed using multiple imputation.
[c]competing-risks regression using *stcrreg* command in Stata adjusting for exposure group, type of cancer, sex, age and age^2, with death by any other cause but cancer as the competing risk.
[d]primary analysis repeated after introducing a three month lag to exposure.

For peer review only - http://bmjopen.bmj.com/site/about/guidelines.xhtml

In secondary analyses, we stratified by cancer type; there was a statistically significantly (p<0.001) increased mortality rate associated with exposure to VGSC-inhibiting medication across all three cancers, HR (95% CI) for: breast 1.49 (1.43 to 1.54); prostate 1.65 (1.60 to 1.71); and bowel 1.64 (1.57 to 1.71) (Table 2). Similarly, for the outcomes of time to death from specific index cancer (HR 1.58, 95% CI 1.55 to 1.62), cancer as an underlying or contributory cause (1.56, 1.53 to 1.60) and all-cause mortality (1.50, 1.48 to 1.53) (Table 2).

For peer review only

For peer review only - http://bmjopen.bmj.com/site/about/guidelines.xhtml

Table 2. Estimates of the relationship between exposure to VGSC inhibitors and mortality - secondary analyses.

**Secondary analyses**	**HR (95% CI)**	**p-value**
Primary analyses by type of cancer		
Breast		
Scenario 1	1.27 (1.23, 1.32)	<0.001
Scenario 2	1.22 (1.18, 1.27)	<0.001
Scenario 3	1.49 (1.43, 1.54)	<0.001
Prostate		
Scenario 1	1.38 (1.33, 1.42)	<0.001
Scenario 2	1.42 (1.37, 1.47)	<0.001
Scenario 3	1.65 (1.60, 1.71)	<0.001
Bowel		
Scenario 1	1.34 (1.29, 1.40)	<0.001
Scenario 2	1.26 (1.21, 1.32)	<0.001
Scenario 3	1.64 (1.57, 1.71)	<0.001
Death from index cancer (underlying or contributory cause)		
Scenario 1	1.33 (1.31, 1.36)	<0.001
Scenario 2	1.30 (1.27, 1.32)	<0.001
Scenario 3	1.58 (1.55, 1.62)	<0.001
Cancer-specific mortality (underlying or contributory cause)		
Scenario 1	1.33 (1.31, 1.36)	<0.001
Scenario 2	1.29 (1.27, 1.32)	<0.001
Scenario 3	1.56 (1.53, 1.60)	<0.001
All-cause mortality		
Scenario 1	1.34 (1.32, 1.36)	<0.001
Scenario 2	1.28 (1.26, 1.30)	<0.001
Scenario 3	1.50 (1.48, 1.53)	<0.001

For peer review only - http://bmjopen.bmj.com/site/about/guidelines.xhtml

VGSC-inhibiting anticonvulsants and tricyclic antidepressants

Among patients with exposure to anticonvulsants (ever use n=6,391), VGSC inhibitor use was associated with significantly increased risk of death from cancer (HR 1.40, 95% CI 1.34 to 1.48, p<0.001; Supplementary Figure 1A; Table 3). A higher HR was observed among those for whom anticonvulsants were the most frequent prescription for a VGSC inhibitor (1.62, 95% CI 1.53 to 1.72), but lower for recent use (1.11, 95% CI 1.02 to 1.21). Among patients with exposure to tricyclic antidepressants (ever use n=42,715), VGSC inhibitor use was similarly associated with significantly increased risk of death from cancer (HR 1.61, 95% CI 1.5 to 1.65, p<0.001; Supplementary Figure 1B; Table 3); again, a higher HR was associated with tricyclic antidepressants being the most frequent prescription for a VGSC inhibitor (1.67, 95% CI .63 to 1.71), but lower (and non-statistically significant) for recent use (0.98, 95% CI 0.93 to 1.04, p=0.59).

For peer review only - http://bmjopen.bmj.com/site/about/guidelines.xhtml

Table 3. Estimates of the relationship between exposure to VGSC-inhibiting drugs, subdivided by type, and cancer-specific mortality.

VGSC inhibitor drug group	Exposed* (n=53724), n (%)	HR (95% CI) p-value Scenario 1	HR (95% CI) p-value Scenario 2	HR (95% CI) p-value Scenario 3
Ever use				
Antiarrhythmic	15538 (28.9)	0.91 (0.88, 0.95) p<0.001	0.84 (0.80, 0.87) p<0.001	1.02 (0.98, 1.06) p=0.34
Anticonvulsant	6391 (11.9)	1.19 (1.14, 1.24) p<0.001	1.17 (1.12, 1.23) p<0.001	1.40 (1.34, 1.48) p<0.001
Tricyclic antidepressant	42715 (79.5)	1.32 (1.29, 1.35) p<0.001	1.30 (1.27, 1.33) p<0.001	1.61 (1.57, 1.65) p<0.001
Recent use				
Antiarrhythmic	2807 (5.2)	0.95 (0.89, 1.01) p=0.12	0.87 (0.81, 0.93) p<0.001	0.92 (0.86, 0.99) p=0.03
Anticonvulsant	1656 (3.1)	1.14 (1.05, 1.24) p<0.001	1.05 (0.96, 1.15) p=0.27	1.11 (1.02, 1.21) p=0.02
Tricyclic antidepressant	5408 (10.1)	1.01 (0.96, 1.06) p=0.76	0.90 (0.85, 0.95) p<0.001	0.98 (0.93, 1.04) p=0.59
Most common VGSC inhibitor prescription				
Antiarrhythmic	11032 (20.5)	0.94 (0.91, 0.98) p<0.001	0.87 (0.83, 0.91) p<0.001	1.00 (0.95, 1.05) p=0.94
Anticonvulsant	4062 (7.6)	1.41 (1.34, 1.48) p<0.001	1.45 (1.36, 1.53) p<0.001	1.62 (1.53, 1.72) p<0.001
Tricyclic antidepressant	38600 (71.9)	1.36 (1.33, 1.39) p<0.001	1.37 (1.33, 1.40) p<0.001	1.67 (1.63, 1.71) p<0.001

*Figures in this column relate to the number of patients recorded as having at least some follow-up time considered as exposed to the drug class of interest in Scenario 1 for each definition (ever use, recent use, most common), as a percentage of the whole 'ever' exposed group. The number of patients with any person-time of follow-up considered as exposed for each drug class will be lower in Scenario 2, and fewer still in Scenario 3.

For peer review only - http://bmjopen.bmj.com/site/about/guidelines.xhtml

A total of 12,140 patients received VGSC-inhibiting drug prescriptions solely in the form of amide or ester local anaesthetic injections following their cancer diagnosis, of which 3,656 (30.1%) died with (any) cancer as the underlying cause. Exposure to these injections was associated with a statistically significantly increased risk of death from any cancer (HR 1.49, 95% CI 1.43 to 1.55, p<0.001).

Class 1-3 antiarrhythmics

In contrast to the VGSC-inhibiting anticonvulsants and tricyclic antidepressants, exposure to VGSC-inhibiting antiarrhythmic drugs was associated with decreased risk of cancer-specific mortality (recent use HR 0.92, 95% CI 0.86 to 0.99, p=0.03), or no difference (Supplementary Figure 1C; Table 3). In exploratory analyses, these drugs were separated into their Vaughan Williams classes (Table 4, Supplementary Figures 2A-2D) (37,38). Exposure to Class 1a antiarrhythmic drugs (n=188) had no impact on cancer-specific survival (ever use HR 1.05, 95% CI 0.76 to 1.46, p=0.77; Supplementary Figure 2A; Table 4). Exposure to Class 1b drugs (n=1088), some of which are also indicated as anticonvulsants (e.g. phenytoin), was associated with significantly reduced cancer-specific survival (ever use HR 2.06, 95% CI 1.88 to 2.26, p<0.001; Supplementary Figure 2B; Table 4). In contrast, exposure to Class 1c drugs (n=860) was associated with significantly improved cancer-specific survival (ever use HR 0.75, 95% CI 0.64 to 0.88, p<0.001; Supplementary Figure 2C; Table 4). The Class 1d drug ranolazine (n=165) was associated with significantly improved cancer-specific survival (ever use HR 0.54, 95% CI 0.33 to 0.88, p=0.01; Supplementary Figure 2D; Table 4). However, Class 2 drugs (beta blockers; n=11,643) were not associated with cancer-specific survival (ever use HR 0.99, 95% CI 0.94 to 1.04, p=0.70; Table 4). Finally, Class 3 drugs (which are also K^+ channel blockers; n=3532) also were not associated with cancer-specific survival, (ever use HR 1.06, 95% CI 0.98 to 1.13, p=0.14; Table 4).

For peer review only - http://bmjopen.bmj.com/site/about/guidelines.xhtml

Table 4. Estimates of the relationship between exposure to antiarrhythmic drugs, subdivided by Vaughan Williams classification, and cancer-specific mortality.

Vaughan Williams drug groups	Exposed[a] (n=53724) n (%)	HR (95% CI) p-value Scenario 1	HR (95% CI) p-value Scenario 2	HR (95% CI) p-value Scenario 3
Ever use				
1a Fast VGSC block, K^+ channel block	188 (0.3)	0.94 (0.73, 1.21) p=0.64	0.88 (0.64, 1.22) p=0.45	1.05 (0.76, 1.46) p=0.77
1b[b] VGSC block, fast association/ disassociation	1088 (2.0)	1.82 (1.67, 1.99) p<0.001	1.84 (1.68, 2.02) p<0.001	2.06 (1.88, 2.26) p<0.001
1c VGSC block, slow association/ disassociation	860 (1.6)	0.73 (0.64, 0.84) p<0.001	0.67 (0.57, 0.78) p<0.001	0.75 (0.64, 0.88) p<0.001
1d Persistent current block	165 (0.3)	0.41 (0.25, 0.68) p<0.001	0.42 (0.26, 0.68) p<0.001	0.54 (0.33, 0.88) p=0.01
2 Beta adrenergic block	11643 (21.7)	0.87 (0.84, 0.91) p<0.001	0.79 (0.75, 0.83) p<0.001	0.99 (0.94, 1.04) p=0.70
3 K^+ channel block	3532 (6.6)	0.98 (0.92, 1.04) p=0.50	0.92 (0.85, 0.98) p=0.02	1.06 (0.98, 1.13) p=0.14
Recent use				
1a	45 (0.1)	1.17 (0.73, 1.88) p=0.52	1.00 (0.59, 1.69) p=1.00	1.06 (0.63, 1.79) p=0.83
1b[a]	429 (0.8)	1.22 (1.05, 1.42) p=0.01	1.15 (0.98, 1.34) p=0.10	1.21 (1.03, 1.42) p=0.02
1c	298 (0.6)	0.82 (0.66, 1.01) p=0.06	0.80 (0.65, 0.99) p=0.04	0.84 (0.68, 1.04) p=0.11
1d	4 (0.0)	-	-	-
2	1752 (3.3)	0.92 (0.84, 1.00) p=0.06	0.84 (0.77, 0.92) p<0.001	0.89 (0.81, 0.98) p=0.01
3	738 (1.4)	1.03 (0.92, 1.16) p=0.59	0.94 (0.83, 1.07) p=0.37	1.01 (0.89, 1.14) p=0.91
Most common VGSC inhibitor prescription				
1a	107 (0.2)	1.06 (0.77, 1.45) p=0.71	0.97 (0.62, 1.52) p=0.89	1.10 (0.70, 1.73) p=0.67

For peer review only - http://bmjopen.bmj.com/site/about/guidelines.xhtml

1b[a]	756 (1.4)	1.95 (1.76, 2.16) p<0.001	1.97 (1.76, 2.19) p<0.001	2.16 (1.94, 2.41) p<0.001
1c	632 (1.2)	0.76 (0.65, 0.89) p<0.001	0.71 (0.59, 0.86) p<0.001	0.78 (0.65, 0.94) p=0.01
1d	126 (0.2)	0.43 (0.24, 0.78) p=0.01	0.44 (0.24, 0.79) p=0.01	0.57 (0.31, 1.02) p=0.06
2	8025 (14.9)	0.92 (0.88, 0.96) p<0.001	0.84 (0.79, 0.89) p<0.001	1.01 (0.95, 1.07) p=0.84
3	2786 (5.2)	1.03 (0.96, 1.10) p=0.46	0.96 (0.89, 1.04) p=0.34	1.08 (1.00, 1.17) p=0.06

[a]Figures in this column relate to the number of patients recorded as having at least some follow-up time considered as exposed to the drug group of interest in Scenario 1 for each definition (ever use, recent use, most common), as a percentage of the whole 'ever' exposed group. The number of patients with any person-time of follow-up considered as exposed for each drug group will be lower in Scenario 2, and fewer still in Scenario 3.
[b]Excluding lidocaine, which is commonly prescribed as a local anaesthetic.

For peer review only

Non-VGSC-targeting antiepileptic medications and other Na^+ channel blockers

To investigate whether the reduced cancer-specific survival of patients exposed to VGSC-inhibiting anticonvulsants is attributable to their Na^+ current-inhibiting action, we considered the impact of two other drug groups: (1) anticonvulsants that do not target VGSCs; and (2) drugs that target other types of Na^+ channels, independent of VGSCs. A third (n=46,017, 34.6%) of patients had a prescription for a non-VGSC-targeting anticonvulsant, and 7% (n=9,256) for a non-VGSC-targeting Na^+ channel blocker (Supplementary Table 1). For both drug groups, there was a higher proportion of deaths (from any cause) among those exposed than among those not exposed, and this was true when cancer was considered among the causes of death (Supplementary Table 4). Among those who died, patients exposed to a non-VGSC-targeting antiepileptic medication were more likely to die with any cancer as an underlying cause than unexposed patients (71.1% versus 57.7%); whereas patients exposed to a non-VGSC-targeting Na^+ channel blocker were less likely (51.7% versus 64.1%). Exposure to both drug groups was associated with increased risk of cancer-specific mortality (HR 4.60, 95% CI 4.51 to 4.70, $p<0.001$ for non-VGSC-inhibiting anticonvulsants; and 1.42, 95% CI 1.35 to 1.49, $p<0.001$ for non-VGSC-inhibiting Na^+ channel blockers; Supplementary Figure 3A, B; Table 5). Findings are presented by drug class in Supplementary Table 5.

For peer review only - http://bmjopen.bmj.com/site/about/guidelines.xhtml

Table 5. Estimates of the relationship between exposure to non-VGSC-inhibiting anticonvulsants, non-VGSC-inhibiting Na^+ channel blockers and cancer-specific mortality.

Cancer-specific mortality (underlying cause)	Non-VGSC-inhibiting anticonvulsant		Non-VGSC-inhibiting Na^+ channel blocker	
	HR (95% CI)	p-value	HR (95% CI)	p-value
Scenario 1	2.98 (2.92, 3.04)	<0.001	1.25 (1.21, 1.30)	<0.001
Scenario 2	3.60 (3.53, 3.68)	<0.001	1.32 (1.26, 1.39)	<0.001
Scenario 3	4.60 (4.51, 4.70)	<0.001	1.42 (1.35, 1.49)	<0.001

For peer review only

For peer review only - http://bmjopen.bmj.com/site/about/guidelines.xhtml

DISCUSSION

This study shows that exposure to VGSC-inhibiting drugs (anticonvulsants, local anaesthetics and tricyclic antidepressants) in breast, bowel and prostate cancer patients is associated with a statistically significant increased risk of death from cancer. This risk is elevated for patients who were exposed to this class of medication before, as well as after, their cancer diagnosis. In addition, both non-VGSC-targeting anticonvulsants and non-VGSC-targeting Na^+ channel blockers are associated with significantly increased risk of death from cancer. Notably, the risk of death from cancer is approximately two times higher for non-VGSC-targeting vs. VGSC-inhibiting anticonvulsants. In contrast, VGSC-inhibiting antiarrhythmic medications display a different pattern, and are associated with moderately improved cancer-specific survival. When subdivided according to the updated Vaughan Williams classification, Class 1c and 1d VGSC inhibitors (which have slow receptor association/disassociation, producing persistent current block) are associated with significantly improved cancer-specific survival in several scenarios.

Strengths and weaknesses of the study

The study uses data from the CPRD, the largest prospectively collected primary care database in the UK containing information on causes of death, comorbidities, and drug exposure based on prescription data (39,40). In addition, we studied cancer-specific mortality in addition to overall mortality, and we controlled for other potentially confounding life-limiting indications for which VGSC-inhibiting medications are prescribed (5,6,31,41). A key limitation of observational studies of drug effects on survival is immortal time bias, where patients in the exposed group can enter an “immortal” period in the follow up time between index diagnosis and first prescription of the drug under study (42). We implemented a person-time approach to control for this issue, where exposure status was considered as a time-dependent covariate (31,42). However, this adjustment did not alter the overall conclusions. We also conducted analyses that added a lag of three months to exposure to minimise issues of reverse causation; again, conclusions were unchanged.

For peer review only - http://bmjopen.bmj.com/site/about/guidelines.xhtml

There are several important limitations to the study. Firstly, GP records, including diagnostic codes, covariate data and prescription information, may be incomplete or contain errors. Additionally, a prescription record does not account for non-adherence, and so exposure to the drugs of interest is inferred. Secondly, although the dataset was linked to causes of death, it was not linked to secondary care databases, including the National Cancer Data Repository (43), and so we did not have access to information on cancer stage, progression or treatment. Thirdly, although we were able to identify those cancer patients who had a diagnostic code for a confounding life-limiting indication, for example epilepsy, we had limited information on the severity of the conditions, which is linked to both medication use and survival. It is possible that additional uncontrolled confounding factors in the population may underlie the associations, for example cardiovascular complications (44,45), underscoring a key problem with such retrospective cohort studies. We also did not measure metastasis directly, hence further work is required to establish why cancer patients exposed to these medications have altered survival.

Comparison to other studies

Our findings partially agree with our previous study showing that exposure to VGSC-inhibiting medications is associated with reduced overall survival of cancer patients (29,46). Refinements to the design of the current study, including adjustment for epilepsy diagnosis, and analysis of cancer-specific survival in addition to overall survival (31), did not alter this conclusion. However, subdividing VGSC inhibitors according to their primary indication revealed positive associations between exposure to antiarrhythmics (in particular Class 1c and 1d drugs) and cancer-specific survival. In addition, the current study showed for the first time that the negative association between anticonvulsant exposure and cancer-specific survival was greater for non-VGSC-targeting anticonvulsants than for VGSC-inhibiting anticonvulsants. A number of preclinical studies indicate that VGSC-inhibiting medications reduce survival, proliferation, migration, invasion and metastasis of cancer cells (13–15,47–50). These would support the hypothesis that such drugs may have value as anti-metastatic

For peer review only - http://bmjopen.bmj.com/site/about/guidelines.xhtml

agents. In addition, several clinical studies have shown valproate, another VGSC blocker, to have anti-tumour activity (23,51–53). However, this may, at least partially, be as a result of its action as a histone deacetylase inhibitor (15,54).

Implications for clinical practice

The disagreement between the preclinical observations and the primary care data presented here raises the possibility that any beneficial effect of VGSC-inhibiting medications on cancer progression may be masked by larger effects in the population. We previously postulated that estimation of a positive association may be affected by confounding by indication (29). VGSC-inhibiting medications are indicated primarily for epilepsy, but are also prescribed for other life-limiting conditions, including cardiac arrhythmias, ALS and neuropathic pain/painful neuropathy (5,6,31,41). Epilepsy patients have an elevated risk of death from all causes, including cancer, compared to the general population (standardised mortality ratio >2.2) (30,55,56), possibly due to a poorer general health and/or social status (44,57,58). Adjustment for comorbidities and social deprivation had no effect on the relationship between exposure and reduced survival. In addition, several VGSC-inhibiting antiepileptic drugs, including carbamazepine and phenytoin, can induce activity of the hepatic cytochrome P450 isoenzyme system, which in turn metabolises certain chemotherapeutic agents, including camptothecin analogues, methotrexate, taxanes, teniposide and vinca alkaloids (59,60). Some VGSC inhibitors, including phenytoin, have also been shown to impact on immune function (61). Alterations in bioavailability and efficacy of such agents in the presence of VGSC inhibitors, as well as potential interactions with other treatments, should be studied further.

The observation that non-VGSC-targeting anticonvulsants were associated with worse survival than VGSC-inhibiting anticonvulsants raises the possibility that VGSC inhibition may indeed be beneficial in this cohort of cancer patients, thus indirectly supporting the preclinical hypothesis (9). Moreover, the improved cancer-specific survival of patients exposed to Class 1c and 1d antiarrhythmics, which preferentially target the persistent Na^+ current that is

For peer review only - http://bmjopen.bmj.com/site/about/guidelines.xhtml

responsible for VGSC-dependent metastatic behaviour in preclinical models (11,14,37), further supports the notion that inhibition of these channels may be beneficial in the clinical setting. However, we cannot exclude the possibility that other confounders may exist between patients within these subgroups, for example epilepsy or cardiac arrhythmia severity.

CONCLUSIONS

The unique positive association between antiarrhythmic drug prescriptions and improved survival may point to a specific beneficial effect of certain VGSC inhibitors with this indication, e.g. ranolazine (14,21), and warrants further investigation. These results should be replicated in a study with robust cancer stage data, and an appropriately designed and controlled prospective clinical trial to establish the effect of VGSC inhibition on tumour progression. Such a trial would separate possible uncontrolled confounding from cancer-specific mortality, and could also exploit emerging novel pathophysiological biomarkers of disease progression, for example circulating tumour DNA and ^{23}Na-MRI.

ADDITIONAL INFORMATION

Acknowledgements

The authors acknowledge the MRC and the Wellcome Trust for funding this study.

Author Contributions

CF, TD and WB had the original idea for this study. CF conducted the analysis under supervision of TD and WB. CF, TD and WB wrote the draft of the manuscript. IW, FM and MB contributed to the development of the idea, the study design, interpretation of the findings and revising the manuscript. All authors approved the final submitted version of the manuscript.

For peer review only

Ethics approval and consent to participate

This study was performed following ethics approval from the Department of Biology Ethics Committee, University of York (WB201909). GPs do not seek individual patient consent when they share de-identified data with the CPRD (CPRD policy here: https://www.cprd.com/public). The study was performed in accordance with the Declaration of Helsinki.

Consent for publication

Not applicable.

Data availability

The datasets used and/or analysed during the current study are available from the corresponding author on reasonable request.

Competing interests

The authors declare no competing interests.

Funding information

This work was supported by the Medical Research Council (G1000508) and Wellcome Trust through the Centre for Future Health at the University of York (award number not available).

REFERENCES

1. Fidler IJ, Kripke ML. The challenge of targeting metastasis. Cancer Metastasis Rev. 2015 Dec;34(4):635–41.

2. Anderson RL, Balasas T, Callaghan J, Coombes RC, Evans J, Hall JA, et al. A framework for the development of effective anti-metastatic agents. Nat Rev Clin Oncol. 2019 Mar;16(3):185–204.

3. Capatina AL, Lagos D, Brackenbury WJ. Targeting Ion Channels for Cancer Treatment: Current Progress and Future Challenges. Rev Physiol Biochem Pharmacol [Internet]. 2020 Sep 1;2020/09/01. Available from: http://dx.doi.org/10.1007/112_2020_46

4. Hille B. Ionic channels of excitable membranes. 2nd ed. Vol. 2nd. Sunderland (Massachusetts): Sinauer Associates Inc.; 1992.

For peer review only

5. Mantegazza M, Curia G, Biagini G, Ragsdale DS, Avoli M. Voltage-gated sodium channels as therapeutic targets in epilepsy and other neurological disorders. Lancet Neurol. 2010 Apr;9(4):413–24.

6. George AL Jr. Inherited disorders of voltage-gated sodium channels. J Clin Invest. 2005 Aug;115(8):1990–9.

7. Leslie TK, James AD, Zaccagna F, Grist JT, Deen S, Kennerley A, et al. Sodium homeostasis in the tumour microenvironment. Biochim Biophys Acta Rev Cancer. 2019;1872(2):188304.

8. Yang M, Brackenbury WJ. Membrane potential and cancer progression. Front Physiol. 2013;4:185.

9. Djamgoz MBA, Fraser SP, Brackenbury WJ. In Vivo Evidence for Voltage-Gated Sodium Channel Expression in Carcinomas and Potentiation of Metastasis. Cancers [Internet]. 2019;11(11). Available from: https://www.ncbi.nlm.nih.gov/pubmed/31661908

10. Besson P, Driffort V, Bon E, Gradek F, Chevalier S, Roger S. How do voltage-gated sodium channels enhance migration and invasiveness in cancer cells? Biochim Biophys Acta. 2015 Oct;1848(10 Pt B):2493–501.

11. Yang M, James AD, Suman R, Kasprowicz R, Nelson M, O'Toole PJ, et al. Voltage-dependent activation of Rac1 by Nav 1.5 channels promotes cell migration. J Cell Physiol. 2020 Apr;235(4):3950–72.

12. Lopez-Charcas O, Pukkanasut P, Velu SE, Brackenbury WJ, Hales TG, Besson P, et al. Pharmacological and nutritional targeting of voltage-gated sodium channels in the treatment of cancers. iScience [Internet]. 2021 Apr 23 [cited 2021 Mar 22];24(4). Available from: https://cell.com/iscience/retrieve/pii/S2589004221002388?_returnURL=https%3A%2F%2Flinkinghub.elsevier.com%2Fretrieve%2Fpii%2FS2589004221002388%3Fshowall%3Dtrue

13. Nelson M, Yang M, Dowle AA, Thomas JR, Brackenbury WJ. The sodium channel-blocking antiepileptic drug phenytoin inhibits breast tumour growth and metastasis. Mol Cancer. 2015;14(1):13.

14. Driffort V, Gillet L, Bon E, Marionneau-Lambot S, Oullier T, Joulin V, et al. Ranolazine inhibits NaV1.5-mediated breast cancer cell invasiveness and lung colonization. Mol Cancer. 2014;13(1):264.

15. Martin F, Ufodiama C, Watt I, Bland M, Brackenbury WJ. Therapeutic value of voltage-gated sodium channel inhibitors in breast, colorectal and prostate cancer: a systematic review. Front Pharmacol. 2015;6:273.

16. Chamaraux-Tran TN, Mathelin C, Aprahamian M, Joshi GP, Tomasetto C, Diemunsch P, et al. Antitumor Effects of Lidocaine on Human Breast Cancer Cells: An In Vitro and In Vivo Experimental Trial. Anticancer Res. 2018 Jan;38(1):95–105.

17. Johnson MZ, Crowley PD, Foley AG, Xue C, Connolly C, Gallagher HC, et al. Effect of perioperative lidocaine on metastasis after sevoflurane or ketamine-xylazine anaesthesia for breast tumour resection in a murine model. Br J Anaesth. 2018 Jul;121(1):76–85.

18. Freeman J, Crowley PD, Foley AG, Gallagher HC, Iwasaki M, Ma D, et al. Effect of

For peer review only - http://bmjopen.bmj.com/site/about/guidelines.xhtml

Perioperative Lidocaine and Cisplatin on Metastasis in a Murine Model of Breast Cancer Surgery. Anticancer Res. 2018 Oct;38(10):5599–606.

19. Freeman J, Crowley PD, Foley AG, Gallagher HC, Iwasaki M, Ma D, et al. Effect of Perioperative Lidocaine, Propofol and Steroids on Pulmonary Metastasis in a Murine Model of Breast Cancer Surgery. Cancers [Internet]. 2019 May 1;11(5). Available from: http://dx.doi.org/10.3390/cancers11050613

20. Tada M, Imazeki F, Fukai K, Sakamoto A, Arai M, Mikata R, et al. Procaine inhibits the proliferation and DNA methylation in human hepatoma cells. Hepatol Int. 2007 Sep;1(3):355–64.

21. Bugan I, Kucuk S, Karagoz Z, Fraser SP, Kaya H, Dodson A, et al. Anti-metastatic effect of ranolazine in an in vivo rat model of prostate cancer, and expression of voltage-gated sodium channel protein in human prostate. Prostate Cancer Prostatic Dis [Internet]. 2019;2019/03/22. Available from: http://dx.doi.org/10.1038/s41391-019-0128-3

22. Raderer M, Depisch D, Haider K, Kwasny W, Djavanmard M, Scheithauer W. A Phase I/II Study of Quinidine, a Potential Multidrug Resistance-Reversing Agent, in Combination with Pirarubicin in Patients with Advanced Refractory Breast Cancer. Onkologie. 1993;16:450–3.

23. Wheler JJ, Janku F, Falchook GS, Jackson TL, Fu S, Naing A, et al. Phase I study of anti-VEGF monoclonal antibody bevacizumab and histone deacetylase inhibitor valproic acid in patients with advanced cancers. Cancer Chemother Pharmacol. 2014 Mar;73(3):495–501.

24. Takada M, Fujimoto M, Motomura H, Hosomi K. Inverse Association between Sodium Channel-Blocking Antiepileptic Drug Use and Cancer: Data Mining of Spontaneous Reporting and Claims Databases. Int J Med Sci. 2016;13(1):48–59.

25. Walker AJ, Card T, Bates TE, Muir K. Tricyclic antidepressants and the incidence of certain cancers: a study using the GPRD. Br J Cancer. 2011;104(1):193–7.

26. Exadaktylos AK, Buggy DJ, Moriarty DC, Mascha E, Sessler DI. Can anesthetic technique for primary breast cancer surgery affect recurrence or metastasis? Anesthesiology. 2006 Oct;105(4):660–4.

27. Biki B, Mascha E, Moriarty DC, Fitzpatrick JM, Sessler DI, Buggy DJ. Anesthetic technique for radical prostatectomy surgery affects cancer recurrence: a retrospective analysis. Anesthesiology. 2008 Aug;109(2):180–7.

28. Reddy JP, Dawood S, Mitchell M, Debeb BG, Bloom E, Gonzalez-Angulo AM, et al. Antiepileptic drug use improves overall survival in breast cancer patients with brain metastases in the setting of whole brain radiotherapy. Radiother Oncol. 2015 Nov;117(2):308–14.

29. Fairhurst C, Watt I, Martin F, Bland M, Brackenbury WJ. Sodium channel-inhibiting drugs and survival of breast, colon and prostate cancer: a population-based study. Sci Rep. 2015;5:16758.

30. Neligan A, Bell GS, Johnson AL, Goodridge DM, Shorvon SD, Sander JW. The long-term risk of premature mortality in people with epilepsy. Brain. 2011 Feb;134(Pt 2):388–95.

For peer review only - http://bmjopen.bmj.com/site/about/guidelines.xhtml

31. Fairhurst C, Martin F, Watt I, Doran T, Bland M, Brackenbury WJ. Sodium channel-inhibiting drugs and cancer survival: protocol for a cohort study using the CPRD primary care database. BMJ Open. 2016;6(9):e011661.

32. Suissa S. Immortal time bias in pharmaco-epidemiology. Am J Epidemiol. 2008 Feb 15;167(4):492–9.

33. College Station, TX: StataCorp LP. Stata Statistical Software: Release 14. 2015.

34. Simon R, Makuch RW. A non-parametric graphical representation of the relationship between survival and the occurrence of an event: application to responder versus non-responder bias. Stat Med. 1984 Jan;3(1):35–44.

35. Fine JP, Gray RJ. A Proportional Hazards Model for the Subdistribution of a Competing Risk. J Am Stat Assoc. 1999 Jun 1;94(446):496–509.

36. Chubak J, Boudreau DM, Wirtz HS, McKnight B, Weiss NS. Threats to validity of nonrandomized studies of postdiagnosis exposures on cancer recurrence and survival. J Natl Cancer Inst. 2013 Oct 2;105(19):1456–62.

37. Lei M, Wu L, Terrar DA, Huang CL-H. Modernized Classification of Cardiac Antiarrhythmic Drugs. Circulation. 2018 Oct 23;138(17):1879–96.

38. Vaughan Williams EM. Classification of antidysrhythmic drugs. Pharmacol Ther B. 1975;1(1):115–38.

39. Herrett E, Gallagher AM, Bhaskaran K, Forbes H, Mathur R, van Staa T, et al. Data Resource Profile: Clinical Practice Research Datalink (CPRD). Int J Epidemiol. 2015 Jun;44(3):827–36.

40. Jick SS, Kaye JA, Vasilakis-Scaramozza C, Garcia Rodriguez LA, Ruigomez A, Meier CR, et al. Validity of the general practice research database. Pharmacotherapy. 2003 May;23(5):686–9.

41. Clare JJ, Tate SN, Nobbs M, Romanos MA. Voltage-gated sodium channels as therapeutic targets. Drug Discov Today. 2000;5(11):506–20.

42. Suissa S. Immortal time bias in observational studies of drug effects. Pharmacoepidemiol Drug Saf. 2007 Mar;16(3):241–9.

43. Morris EJ, Jordan C, Thomas JD, Cooper M, Brown JM, Thorpe H, et al. Comparison of treatment and outcome information between a clinical trial and the National Cancer Data Repository. Br J Surg. 2011 Feb;98(2):299–307.

44. Keezer MR, Sisodiya SM, Sander JW. Comorbidities of epilepsy: current concepts and future perspectives. Lancet Neurol. 2016 Jan;15(1):106–15.

45. Shmuely S, van der Lende M, Lamberts RJ, Sander JW, Thijs RD. The heart of epilepsy: Current views and future concepts. Seizure. 2017 Jan;44:176–83.

46. Fairhurst C, Watt I, Martin F, Bland M, Brackenbury WJ. Exposure to sodium channel-inhibiting drugs and cancer survival: protocol for a cohort study using the QResearch primary care database. BMJ Open. 2014;4(11):e006604.

47. Yang M, Kozminski DJ, Wold LA, Modak R, Calhoun JD, Isom LL, et al. Therapeutic potential for phenytoin: targeting Na(v)1.5 sodium channels to reduce migration and invasion in metastatic breast cancer. Breast Cancer Res Treat. 2012 Jul;134(2):603–15.

For peer review only - http://bmjopen.bmj.com/site/about/guidelines.xhtml

48. Brackenbury WJ. Voltage-gated sodium channels and metastatic disease. Channels . 2012;6(5):352–61.

49. Fraser SP, Diss JK, Chioni AM, Mycielska ME, Pan H, Yamaci RF, et al. Voltage-gated sodium channel expression and potentiation of human breast cancer metastasis. Clin Cancer Res. 2005;11(15):5381–9.

50. Baptista-Hon DT, Robertson FM, Robertson GB, Owen SJ, Rogers GW, Lydon EL, et al. Potent inhibition by ropivacaine of metastatic colon cancer SW620 cell invasion and NaV1.5 channel function. Br J Anaesth. 2014 Jul;113 Suppl 1:i39–48.

51. Tassara M, Dohner K, Brossart P, Held G, Gotze K, Horst HA, et al. Valproic acid in combination with all-trans retinoic acid and intensive therapy for acute myeloid leukemia in older patients. Blood. 2014;123(26):4027–36.

52. Munster P, Marchion D, Bicaku E, Lacevic M, Kim J, Centeno B, et al. Clinical and biological effects of valproic acid as a histone deacetylase inhibitor on tumor and surrogate tissues: phase I/II trial of valproic acid and epirubicin/FEC. Clin Cancer Res. 2009;15(7):2488–96.

53. Munster P, Marchion D, Bicaku E, Schmitt M, Lee JH, DeConti R, et al. Phase I trial of histone deacetylase inhibition by valproic acid followed by the topoisomerase II inhibitor epirubicin in advanced solid tumors: a clinical and translational study. J Clin Oncol. 2007;25(15):1979–85.

54. Michaelis M, Doerr HW, Cinatl J Jr. Valproic acid as anti-cancer drug. Curr Pharm Des. 2007;13(33):3378–93.

55. Shackleton DP, Westendorp RG, Trenite DG, Vandenbroucke JP. Mortality in patients with epilepsy: 40 years of follow up in a Dutch cohort study. J Neurol Neurosurg Psychiatry. 1999 May;66(5):636–40.

56. Nevalainen O, Raitanen J, Ansakorpi H, Artama M, Isojarvi J, Auvinen A. Long-term mortality risk by cause of death in newly diagnosed patients with epilepsy in Finland: a nationwide register-based study. Eur J Epidemiol. 2013 Dec;28(12):981–90.

57. Gaitatzis A, Carroll K, Majeed A, Sander JW. The epidemiology of the comorbidity of epilepsy in the general population. Epilepsia. 2004 Dec;45(12):1613–22.

58. Singh G, Fletcher O, Bell GS, McLean AE, Sander JW. Cancer mortality amongst people with epilepsy: a study of two cohorts with severe and presumed milder epilepsy. Epilepsy Res. 2009 Feb;83(2-3):190–7.

59. Vecht CJ, Wagner GL, Wilms EB. Interactions between antiepileptic and chemotherapeutic drugs. Lancet Neurol. 2003 Jul;2(7):404–9.

60. Singh G, Driever PH, Sander JW. Cancer risk in people with epilepsy: the role of antiepileptic drugs. Brain. 2005 Jan;128(Pt 1):7–17.

61. Beghi E, Shorvon S. Antiepileptic drugs and the immune system. Epilepsia. 2011 May;52 Suppl 3:40–4.

For peer review only

Figure Legends

Figure 1. Simon-Makuch survival curve for unexposed cancer patients and those ever exposed to VGSC-inhibiting drugs in Scenario 3.

For peer review only

For peer review only - http://bmjopen.bmj.com/site/about/guidelines.xhtml

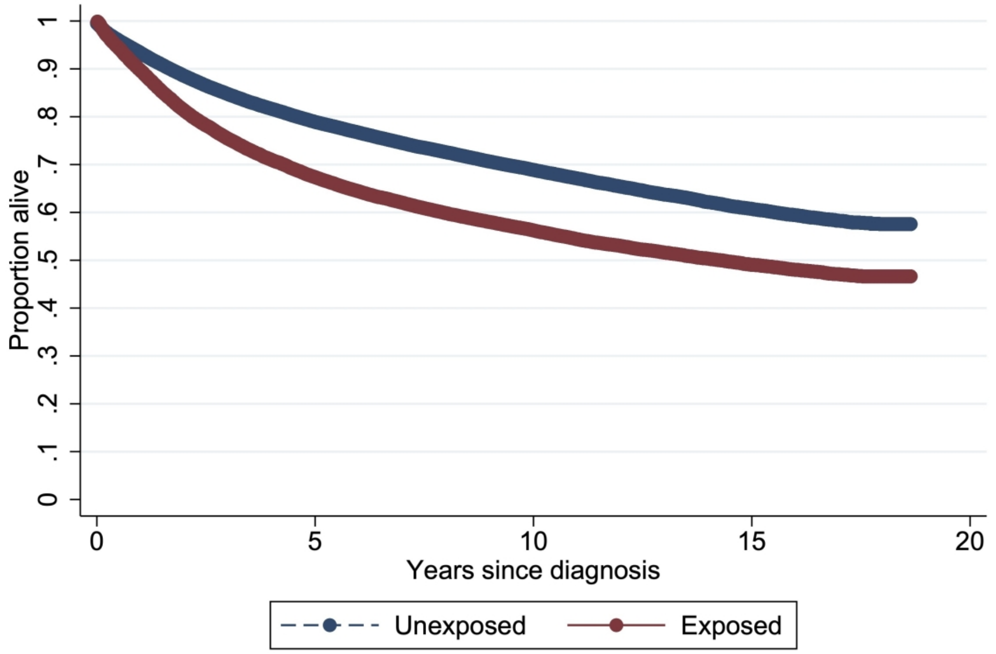

Figure 1

1125x741mm (72 x 72 DPI)

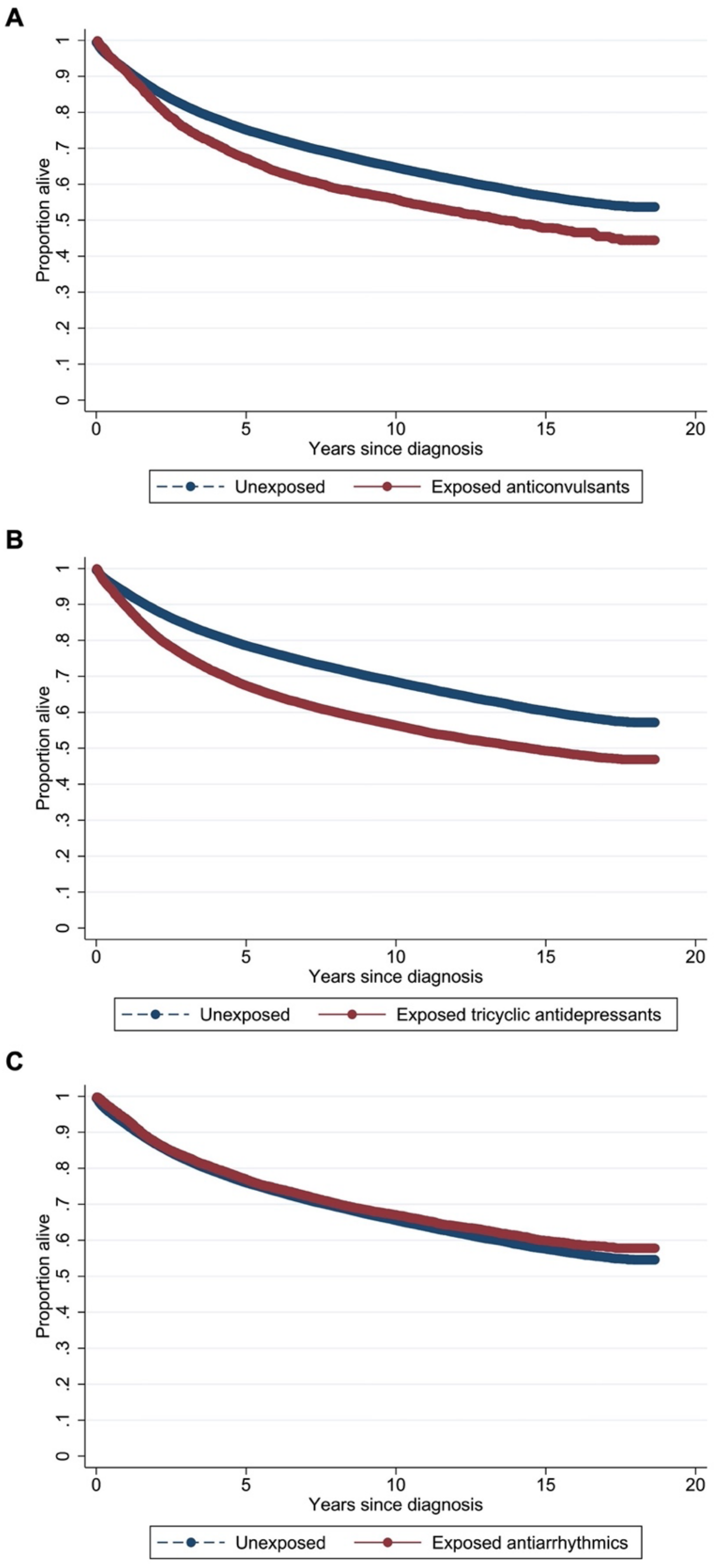

Supplementary Figure 1. Simon-Makuch survival curves for unexposed cancer patients and those ever exposed to VGSC-inhibiting anticonvulsant (A), tricyclic antidepressant (B) and antiarrhythmic (C) drugs in Scenario 3.

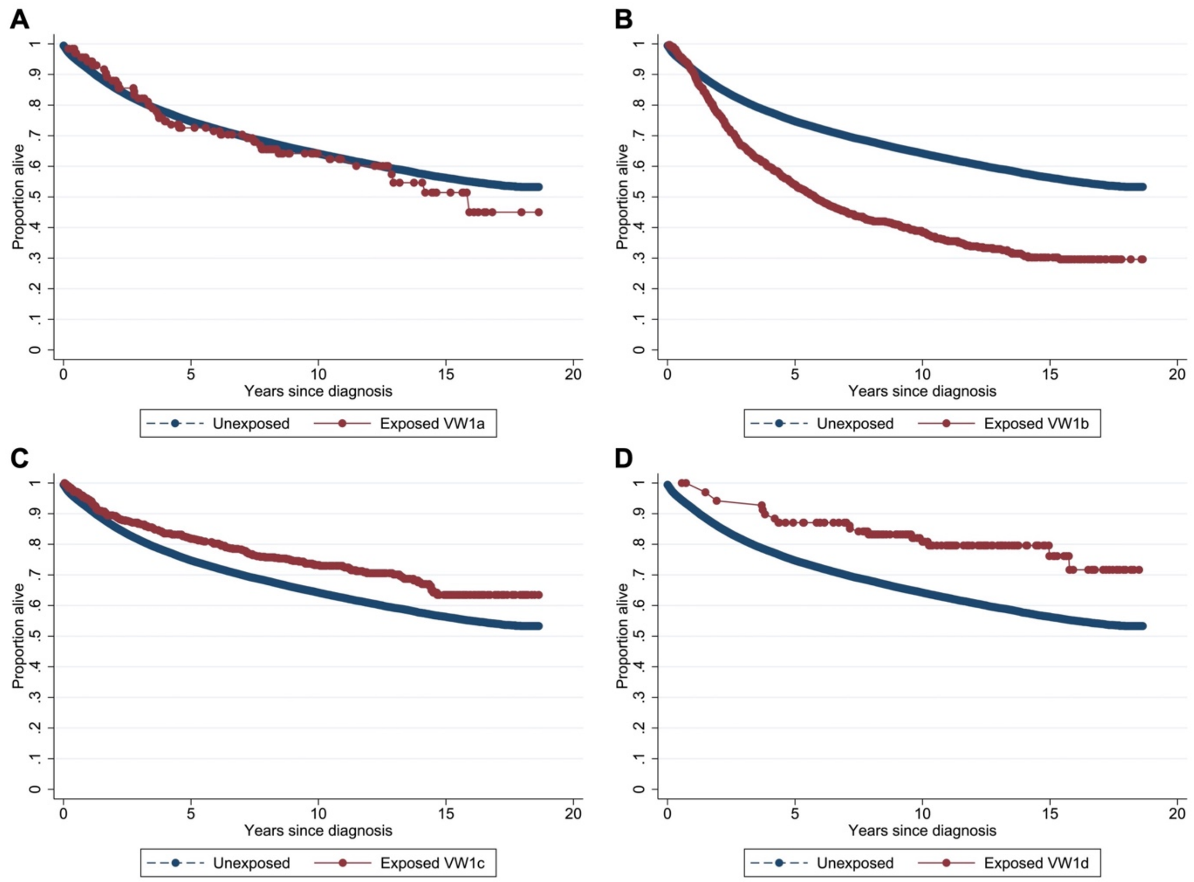

Supplementary Figure 2. Simon-Makuch survival curve for unexposed cancer patients and those ever exposed to Vaughan-Williams Class 1a (A), 1b (B), 1c (C) and 1d (D) drugs in Scenario 3.

For peer review only - http://bmjopen.bmj.com/site/about/guidelines.xhtml

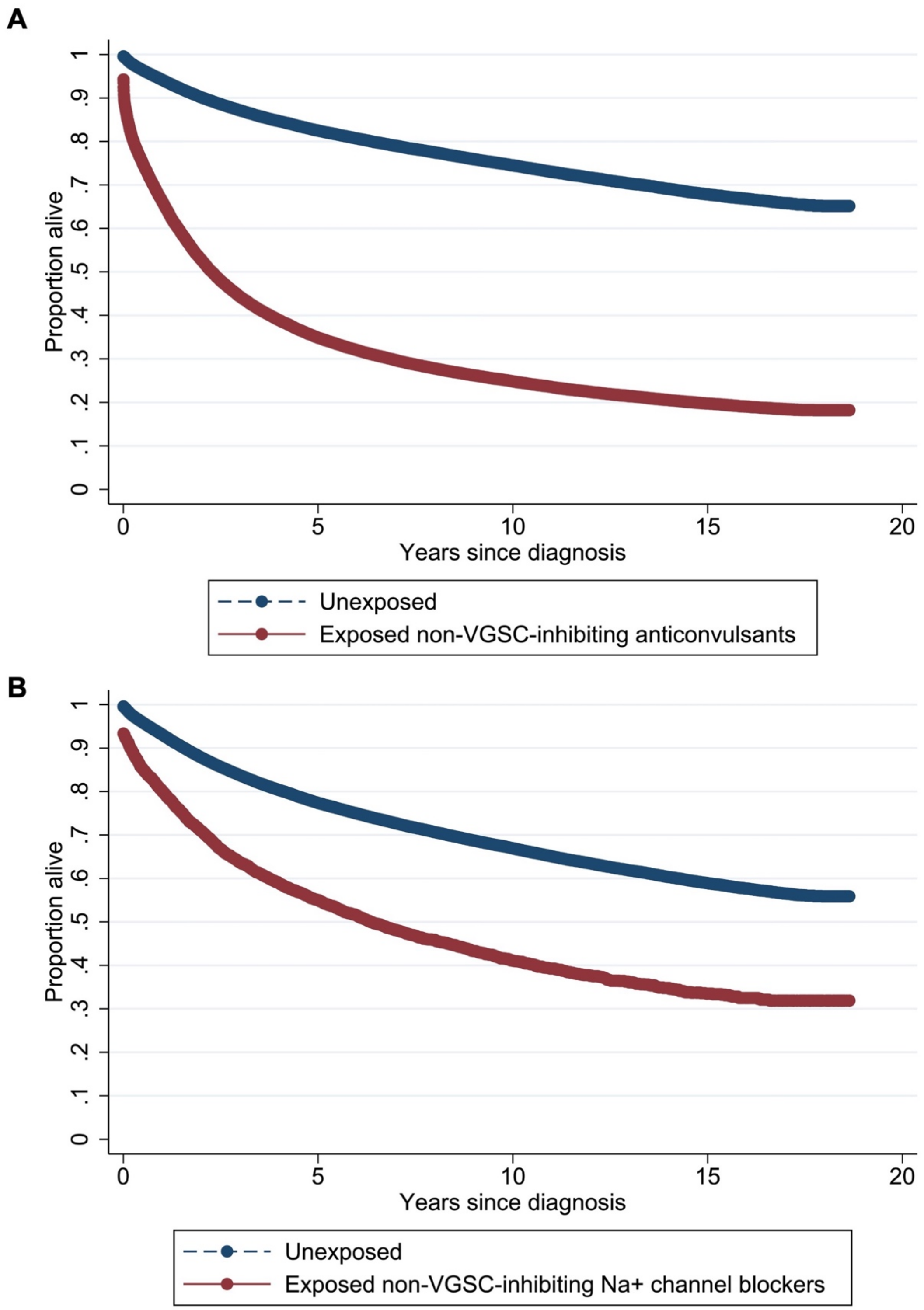

Supplementary Figure 3. Simon-Makuch survival curve for unexposed cancer patients and those ever exposed to non-VGSC-inhibiting anticonvulsants (A) and non-VGSC-inhibiting Na^+ channel blockers (B) in Scenario 3.

For peer review only - http://bmjopen.bmj.com/site/about/guidelines.xhtml

Supplementary Table 1. Drug groups and classifications used in this study.

Drug	Classification	Vaughan Williams Classification[a]
**A. VGSC inhibitors**		
Articaine	Amide local anaesthetic	
Bupivacaine	Amide local anaesthetic	
Cinchocaine	Amide local anaesthetic	
Etidocaine	Amide local anaesthetic	
Levobupivacaine	Amide local anaesthetic	
Lidocaine	Amide local anaesthetic	1b
Mepivacaine	Amide local anaesthetic	
Prilocaine	Amide local anaesthetic	
Ropivacaine	Amide local anaesthetic	
Trimecaine	Amide local anaesthetic	
Ranolazine	Antiarrhythmic	1d
Ajmaline	Antiarrhythmic	1a
Amiodarone	Antiarrhythmic	3
Aprindine	Antiarrhythmic	1b
Disopyramide	Antiarrhythmic	1a
Dronedarone	Antiarrhythmic	3
Encainide	Antiarrhythmic	1c
Flecainide	Antiarrhythmic	1c
Mexiletine	Antiarrhythmic	1b
Moricizine/Moricizine hydrochloride	Antiarrhythmic	1c
Pilsicainide	Antiarrhythmic	1c
Procainamide	Antiarrhythmic	1a
Propafenone	Antiarrhythmic	1c
Quinidine	Antiarrhythmic	1a
Tocainide	Antiarrhythmic	1b

For peer review only - http://bmjopen.bmj.com/site/about/guidelines.xhtml

Carvedilol	Antiarrhythmic	2
Labetalol	Antiarrhythmic	2
Oxprenolol	Antiarrhythmic	2
Propranolol	Antiarrhythmic	2
Esmolol	Antiarrhythmic	2
Carbamazepine	Anticonvulsant	
Eslicarbazepine	Anticonvulsant	
Eslicarbazepine acetate	Anticonvulsant	
Ethotoin	Anticonvulsant	
Fosphenytoin	Anticonvulsant	
Lacosamide	Anticonvulsant	
Lamotrigine	Anticonvulsant	
Oxcarbazepine	Anticonvulsant	
Phenytoin	Anticonvulsant	1b
Rufinamide	Anticonvulsant	
Sodium Valproate	Anticonvulsant	
Topiramate	Anticonvulsant	
Valproic acid	Anticonvulsant	
Zonisamide	Anticonvulsant	
Benzocaine	Ester local anaesthetic	
Procaine	Ester local anaesthetic	
Tetracaine	Ester local anaesthetic	
Riluzole	ALS treatment	
Amitriptyline	Tricyclic antidepressant	
Desipramine	Tricyclic antidepressant	
Duloxetine	Tricyclic antidepressant	
Fluoxetine	Tricyclic antidepressant	
Imipramine	Tricyclic antidepressant	
Maprotiline	Tricyclic antidepressant	

For peer review only - http://bmjopen.bmj.com/site/about/guidelines.xhtml

Nortriptyline	Tricyclic antidepressant	
**A. Non-VGSC Na^+ channel blocker**		
Amiloride	ENaC inhibitor	
Triamterene	ENaC inhibitor	
**B. Non-VGSC-inhibiting anticonvulsants**		
Perampanel	AMPA receptor non-competitive antagonist	
Stiripentol	Aromatic allylic alcohol	
Phenobarbital	Barbiturate	
Primidone	Barbiturate	
Clobazam	Benzodiazepine	
Clonazepam	Benzodiazepine	
Diazepam	Benzodiazepine	
Lorazepam	Benzodiazepine	
Midazolam	Benzodiazepine	
Ethosuximide	Ca^{2+} channel inhibitor	
Gabapentin	Ca^{2+} channel inhibitor	
Pregabalin	Ca^{2+} channel inhibitor	
Acetazolamide	Carbonic anhydrase inhibitor	
Tiagabine	GABA reuptake inhibitor	
Vigabatrin	GABA reuptake inhibitor	
Brivaracetam	SV2A inhibitor	
Levetiracetam	SV2A inhibitor	

[a]According to (37,38).

For peer review only - http://bmjopen.bmj.com/site/about/guidelines.xhtml

For peer review only

Supplementary Table 2. Characteristics of the participants stratified by exposure status.

	A VGSC-inhibitor prescription (any at any time exc anaesthetics) (n=53724)	No VGSC-inhibitor prescriptions (n=79272)	Total (n=132996)	p-value
**Sex, n (%)**				
Male	22531 (41.9)	41402 (52.2)	63933 (48.1)	<0.001
Female	31193 (58.1)	37870 (47.8)	69063 (51.9)	
**Age at diagnosis, years**				
Mean (SD)	65.9 (13.0)	68.0 (13.3)	67.1 (13.2)	<0.001
**Ethnicity, n (%)**				
White	50495 (94.0)	72056 (90.9)	122551 (92.1)	<0.001
Mixed/Multiple ethnic groups	161 (0.3)	299 (0.4)	460 (0.3)	
Asian/Asian British	801 (1.5)	1297 (1.6)	2098 (1.6)	
Black/Black British	733 (1.4)	1597 (2.0)	2330 (1.8)	
Other	190 (0.4)	336 (0.4)	526 (0.4)	

For peer review only - http://bmjopen.bmj.com/site/about/guidelines.xhtml

Not recorded/known	1344 (2.5)	3687 (4.7)	5031 (3.8)	
**Index of Multiple Deprivation, n (%)**				
Mean (SD)	9.2 (5.6)	9.0 (5.5)	9.1 (5.5)	<0.001
**Smoking status, n (%)**				
Heavy smoker	1596 (3.0)	1648 (2.1)	3244 (2.4)	<0.001
Moderate smoker	4503 (8.4)	5259 (6.6)	9762 (7.3)	
Light smoker	1675 (3.1)	2156 (2.7)	3831 (2.9)	
Ex-smoker	16413 (30.6)	23004 (29.0)	39417 (29.6)	
Non-smoker	28676 (53.4)	44072 (55.6)	72748 (54.7)	
Not recorded/known	861 (1.6)	3133 (4.0)	3994 (3.0)	
**Alcohol intake, n (%)**				
Heavy drinker	13656 (25.4)	20565 (25.9)	34221 (25.7)	<0.001
Moderate drinker	3421 (6.4)	5003 (6.3)	8424 (6.3)	
Light drinker	11829 (22.0)	16231 (20.5)	28060 (21.1)	

For peer review only - http://bmjopen.bmj.com/site/about/guidelines.xhtml

Non drinker	9001 (16.8)	10442 (13.2)	19443 (14.6)	
Not recorded/known	15817 (29.4)	27031 (34.1)	42848 (32.2)	
**BMI category, n (%)**				
Overweight/Obese	32166 (59.9)	42432 (53.5)	74598 (56.1)	<0.001
Normal range	15526 (28.9)	24137 (30.4)	39663 (29.8)	
Underweight	2345 (4.4)	3598 (4.5)	5943 (4.5)	
Not recorded/known	3687 (6.9)	9105 (11.5)	12792 (9.6)	
**Physical activity, n (%)**				
Very active	2634 (4.9)	4173 (5.3)	6807 (5.1)	<0.001
Moderately active	18614 (34.6)	25806 (32.6)	44420 (33.4)	
Inactive	7551 (14.1)	8179 (10.3)	15730 (11.8)	
Not recorded/known	24925 (46.4)	41114 (51.9)	66039 (49.7)	
**Type of cancer, n (%)**				
Breast	27106 (50.5)	32422 (40.9)	59528 (44.8)	<0.001

For peer review only - http://bmjopen.bmj.com/site/about/guidelines.xhtml

Bowel	8435 (15.7)	14432 (18.2)	22867 (17.2)	
Prostate	18183 (33.8)	32418 (40.9)	50601 (38.0)	
**Total CCI score**				
Mean (SD)	6.1 (2.8)	5.9 (2.7)	6.0 (2.7)	<0.001
**VGSC-inhibitor indication[a], n (%)**				
Epilepsy	1915 (3.6)	449 (0.6)	2364 (1.8)	<0.001
Cardiac arrhythmia	9646 (18.0)	9791 (12.4)	19437 (14.6)	<0.001
Amyotrophic lateral sclerosis	0 (0.0)	0 (0.0)	0 (0.0)	-
Neuropathic pain/painful neuropathy	9860 (18.4)	7271 (9.2)	17131 (12.9)	<0.001
**≥1 of above, n (%)**	18744 (34.9)	16048 (20.2)	34792 (26.2)	<0.001

[a] not mutually exclusive

VGSC, voltage gated sodium channel; SD, standard deviation; BMI, body mass index; CCI, Charlson Comorbidity Index score.

For peer review only - http://bmjopen.bmj.com/site/about/guidelines.xhtml

Supplementary Table 3. Characteristics of the 'ever' exposed group stratified by timing of exposure relative to their cancer diagnosis.

	**Before only (n=14,157)**	**Before and after (n=17,264)**	**After only (n=22,303)**	**VGSC inhibitor prescription (any at any time excluding local anaesthetics) (n=53,724)**
**Sex, n (%)**				
Male	6049 (42.7)	6325 (36.6)	10157 (45.5)	22531 (41.9)
Female	8108 (57.3)	10939 (63.4)	12146 (54.5)	31193 (58.1)
**Age at diagnosis, years**				
Mean (SD)	68.6 (13.4)	65.7 (12.9)	64.3 (12.5)	65.9 (13.0)
**Ethnicity, n (%)**				
White	13189 (93.2)	16366 (94.8)	20940 (93.9)	50495 (94.0)
Mixed/Multiple ethnic groups	35 (0.2)	47 (0.3)	79 (0.4)	161 (0.3)
Asian/Asian British	172 (1.2)	252 (1.5)	377 (1.7)	801 (1.5)
Black/Black British	169 (1.2)	186 (1.1)	378 (1.7)	733 (1.4)
Other	48 (0.3)	54 (0.3)	88 (0.4)	190 (0.4)
Not recorded/known	544 (3.8)	359 (2.1)	441 (2.0)	1344 (2.5)

For peer review only - http://bmjopen.bmj.com/site/about/guidelines.xhtml

**Index of Multiple Deprivation, n (%)**				
Mean (SD)	9.2 (5.5)	9.5 (5.7)	9.0 (5.5)	9.2 (5.6)
**Smoking status, n (%)**				
Heavy smoker	373 (2.6)	561 (3.2)	662 (3.0)	1596 (3.0)
Moderate smoker	1139 (8.0)	1554 (9.0)	1810 (8.1)	4503 (8.4)
Light smoker	437 (3.1)	520 (3.0)	718 (3.2)	1675 (3.1)
Ex-smoker	4336 (30.6)	5362 (31.1)	6715 (30.1)	16413 (30.6)
Non-smoker	7562 (53.4)	9020 (52.2)	12094 (54.2)	28676 (53.4)
Not recorded/known	310 (2.2)	247 (1.4)	304 (1.4)	861 (1.6)
**Alcohol intake, n (%)**				
Heavy drinker	3467 (24.5)	4030 (23.3)	6159 (27.6)	13656 (25.4)
Moderate drinker	929 (6.6)	1036 (6.0)	1456 (6.5)	3421 (6.4)
Light drinker	3153 (22.3)	3879 (22.5)	4797 (21.5)	11829 (22.0)
Non drinker	2436 (17.2)	3404 (19.7)	3161 (14.2)	9001 (16.8)
Not recorded/known	4172 (29.5)	4915 (28.5)	6730 (30.2)	15817 (29.4)
**BMI category, n (%)**				

For peer review only - http://bmjopen.bmj.com/site/about/guidelines.xhtml

Overweight/Obese	7990 (56.4)	10699 (62.0)	13477 (60.4)	32166 (59.9)
Normal range	4267 (30.1)	4734 (27.4)	6525 (29.3)	15526 (28.9)
Underweight	764 (5.4)	728 (4.2)	853 (3.8)	2345 (4.4)
Not recorded/known	1136 (8.0)	1103 (6.4)	1448 (6.5)	3687 (6.9)
**Physical activity, n (%)**				
Very active	615 (4.3)	752 (4.4)	1267 (5.7)	2634 (4.9)
Moderately active	4497 (31.8)	5896 (34.2)	8221 (36.9)	18614 (34.6)
Inactive	1785 (12.6)	2816 (16.3)	2950 (13.2)	7551 (14.1)
Not recorded/known	7260 (51.3)	7800 (45.2)	9865 (44.2)	24925 (46.4)
**Type of cancer, n (%)**				
Breast	6766 (47.8)	9642 (55.9)	10698 (48.0)	27106 (50.5)
Bowel	2531 (17.9)	2472 (14.3)	3432 (15.4)	8435 (15.7)
Prostate	4860 (34.3)	5150 (29.8)	8173 (36.6)	18183 (33.8)
**Total CCI score**				
Mean (SD)	6.3 (2.8)	6.1 (2.8)	5.9 (2.8)	6.1 (2.8)
**Diagnosis of a VGSC inhibitor indication[a], n (%)**				

For peer review only - http://bmjopen.bmj.com/site/about/guidelines.xhtml

Epilepsy	202 (1.4)	1271 (7.4)	442 (2.0)	1915 (3.6)
Cardiac arrhythmia	2611 (18.4)	3362 (19.5)	3673 (16.5)	9646 (18.0)
Amyotrophic lateral sclerosis	0 (0.0)	0 (0.0)	0 (0.0)	0 (0.0)
Neuropathic pain/painful neuropathy	2126 (15.0)	3790 (22.0)	3944 (17.7)	9860 (18.4)
**≥1 of above, n (%)**	4400 (31.1)	7199 (41.7)	7145 (32.0)	18744 (34.9)
**Died, n (%)**	7800 (55.1)	8119 (47.0)	10109 (45.3)	26028 (48.4)
**Most common prescription 1**[a]				
Tricyclic antidepressant	9312 (65.8)	11495 (66.6)	17793 (79.8)	38600 (71.8)
Antiarrhythmic	4023 (28.4)	3828 (22.2)	3181 (14.3)	11032 (20.5)
Anticonvulsant	822 (5.8)	1935 (11.2)	1305 (5.9)	4062 (7.6)
Treatment for ALS	0 (0.0)	6 (0.0)	24 (0.1)	30 (0.1)
**Most common prescription 2**[b]				
Tricyclic antidepressant	7887 (55.7)	10831 (62.7)	15187 (68.1)	33905 (63.1)
Antiarrhythmic	3604 (25.5)	3726 (21.6)	2881 (12.9)	10211 (19.0)
Amide local anaesthetic	1905 (13.5)	782 (4.5)	2951 (13.2)	5638 (10.5)

For peer review only - http://bmjopen.bmj.com/site/about/guidelines.xhtml

Anticonvulsant	708 (5.0)	1907 (11.0)	1191 (5.3)	3806 (7.1)
Ester local anaesthetic	53 (0.4)	12 (0.1)	71 (0.3)	136 (0.3)
Treatment for ALS	0 (0.0)	6 (0.0)	22 (0.1)	28 (0.1)
**Length of exposure (days)**				
Mean (SD)	2095.1 (2220.1)	4889.9 (2473.2)	1912.7 (2077.5)	2917.5 (2627.5)
< 6 months, n (%)	3239 (22.9)	40 (0.2)	4589 (20.6)	7868 (14.6)
≥ 6 months, n (%)	10918 (77.1)	17224 (99.8)	17714 (79.4)	45856 (85.4)
**Recent exposure[c], n (%)**	1017 (7.2)	8580 (49.7)	0 (0.0)	9597 (17.9)

[a] excluding local anaesthetics.

[b] including local anaesthetics.

[c] ≥2 prescriptions relating to one of the VGSC-inhibiting drugs within 2 years before the date of the cancer diagnosis, including at least one within 6 months before.

For peer review only

Supplementary Table 4. Deaths stratified by exposure to non-VGSC-inhibiting anticonvulsants and non-VGSC-inhibiting Na^+ channel blockers.

	A VGSC-inhibitor prescription (any other than Amide or Ester local anaesthetics at any time) (n=53724)	No VGSC-inhibitor prescriptions (except Amide or Ester local anaesthetics) (n=79272)	A non-VGSC-inhibiting anticonvulsant prescription (any at any time) (n=46017)	No exposure to a non-VGSC-inhibiting anticonvulsant prescription (n=86979)	Non-VGSC-inhibiting Na^+ channel blocker prescription (any at any time) (n=9256)	No exposure to a non-VGSC-inhibiting Na^+ channel blocker prescription (n=123740)
Died (any cause)	26028 (48.4)	40932 (51.6)	25284 (54.9)	41676 (47.9)	6969 (75.3)	59991 (48.5)
Died with any cancer as underlying cause	15933 (29.7)	26104 (32.9)	17987 (39.1)	24050 (27.7)	3601 (38.9)	38436 (31.1)
Died with any cancer as contributory cause	18598 (34.6)	30492 (38.5)	20003 (43.5)	29087 (33.4)	4540 (49.0)	44550 (36.0)
Died with index cancer as underlying cause	12282 (22.9)	20443 (25.8)	13925 (30.3)	18800 (21.6)	2842 (30.7)	29883 (24.1)

For peer review only - http://bmjopen.bmj.com/site/about/guidelines.xhtml

Died with index cancer as contributory cause	15256 (28.4)	25482 (32.1)	16451 (35.7)	24287 (27.9)	3834 (41.4)	36904 (29.8)

For peer review only

Supplementary Table 5. Estimates of the relationship between exposure to non-VGSC-inhibiting drugs, subdivided by type, and cancer-specific mortality (empty cells indicate that analysis was not permitted due to low numbers).

**Non-VGSC-inhibiting anticonvulsants drug groups**	**Exposed* (n=46017), n (%)**	**HR (95% CI) p-value Scenario 1**	**HR (95% CI) p-value Scenario 2**	**HR (95% CI) p-value Scenario 3**
Ever use				
AMPA receptor non-competitive antagonist	4 (0.1)	-	-	-
Aromatic allylic alcohol	0 (0.0)	-	-	-
Barbiturate	469 (1.0)	1.39 (1.19, 1.63) p<0.001	1.28 (1.07, 1.52) p=0.01	1.42 (1.19, 1.69) p<0.001
Benzodiazepine	37696 (81.9)	3.01 (2.95, 3.07) p<0.001	3.73 (3.65, 3.81) p<0.001	4.91 (4.80, 5.02) p<0.001
Calcium channel inhibitor	11643 (21.7)	2.19 (2.12, 2.26) p<0.001	2.27 (2.19, 2.34) p<0.001	2.80 (2.71, 2.90) p<0.001
Carbonic anhydrase inhibitor	14274 (31.0)	0.92 (0.79, 1.06) p=0.25	0.93 (0.76, 1.13) p=0.46	1.25 (1.02, 1.53) p=0.03
GABA reuptake inhibitor	23 (0.1)	-	-	-
SV2A inhibitor	620 (1.4)	2.98 (2.63, 3.38) p<0.001	3.03 (2.67, 3.44) p<0.001	3.75 (3.30, 4.25) p<0.001
Recent use				

For peer review only - http://bmjopen.bmj.com/site/about/guidelines.xhtml

AMPA receptor non-competitive antagonist	0 (0.0)	-	-	-
Aromatic allylic alcohol	0 (0.0)	-	-	-
Barbiturate	208 (0.5)	1.44 (1.16, 1.78) p<0.001	1.37 (1.10, 1.71) p<0.001	1.46 (1.18, 1.82) p<0.001
Benzodiazepine	2686 (5.8)	1.46 (1.37, 1.56) p<0.001	1.34 (1.25, 1.44) p<0.001	1.51 (1.41, 1.62) p<0.001
Calcium channel inhibitor	902 (2.0)	1.12 (0.99, 1.26) p=0.08	1.06 (0.93, 1.21) p=0.38	1.20 (1.05, 1.37) p=0.01
Carbonic anhydrase inhibitor	43 (0.1)	-	-	-
GABA reuptake inhibitor	0 (0.0)	-	-	-
SV2A inhibitor	54 (0.1)	-	-	-
Most common VGSC-inhibitor prescription				
AMPA receptor non-competitive antagonist	0 (0.0)	-	-	-
Aromatic allylic alcohol	0 (0.0)	-	-	-
Barbiturate	348 (0.8)	1.43 (1.20, 1.71) p<0.001	1.30 (1.07, 1.59) p=0.01	1.40 (1.15, 1.71) p<0.001

For peer review only - http://bmjopen.bmj.com/site/about/guidelines.xhtml

Benzodiazepine	32563 (70.8)	3.06 (2.99, 3.12) p<0.001	4.02 (3.93, 4.11) p<0.001	5.11 (4.99, 5.23) p<0.001
Calcium channel inhibitor	12061 (26.2)	2.22 (2.15, 2.30) p<0.001	2.33 (2.24, 2.41) p<0.001	2.80 (2.70, 2.90) p<0.001
Carbonic anhydrase inhibitor	561 (1.2)	0.92 (0.78, 1.08) p=0.31	0.92 (0.72, 1.18) p=0.52	1.22 (0.96, 1.56) p=0.11
GABA reuptake inhibitor	7 (0.0)	-	-	-
SV2A inhibitor	477 (1.0)	3.16 (2.73, 3.65) p<0.001	3.23 (2.79, 3.74) p<0.001	4.00 (3.45, 4.63) p<0.001

*Figures in this column relate to the number of patients recorded as having at least some follow-up time considered as exposed to the drug group of interest in Scenario 1 for each definition (ever use, recent use, most common), as a percentage of the whole 'ever' exposed group. The number of patients with any person-time of follow-up considered as exposed for each drug group will be lower in Scenario 2, and fewer still in Scenario 3.

For peer review only - http://bmjopen.bmj.com/site/about/guidelines.xhtml

Reporting checklist for cohort study.

Based on the STROBE cohort guidelines.

Instructions to authors

Complete this checklist by entering the page numbers from your manuscript where readers will find each of the items listed below.

Your article may not currently address all the items on the checklist. Please modify your text to include the missing information. If you are certain that an item does not apply, please write "n/a" and provide a short explanation.

Upload your completed checklist as an extra file when you submit to a journal.

In your methods section, say that you used the STROBE cohortreporting guidelines, and cite them as:

von Elm E, Altman DG, Egger M, Pocock SJ, Gotzsche PC, Vandenbroucke JP. The Strengthening the Reporting of Observational Studies in Epidemiology (STROBE) Statement: guidelines for reporting observational studies.

		Reporting Item	Page Number
**Title and abstract**			
Title	#1a	Indicate the study's design with a commonly used term in the title or the abstract	1
Abstract	#1b	Provide in the abstract an informative and balanced summary of what was done and what was found	2
**Introduction**			
Background / rationale	#2	Explain the scientific background and rationale for the investigation being reported	4
Objectives	#3	State specific objectives, including any prespecified hypotheses	4
**Methods**			
Study design	#4	Present key elements of study design early in the paper	5
Setting	#5	Describe the setting, locations, and relevant dates, including periods of	5

For peer review only

		recruitment, exposure, follow-up, and data collection	
Eligibility criteria	#6a	Give the eligibility criteria, and the sources and methods of selection of participants. Describe methods of follow-up.	5
Eligibility criteria	#6b	For matched studies, give matching criteria and number of exposed and unexposed	5
Variables	#7	Clearly define all outcomes, exposures, predictors, potential confounders, and effect modifiers. Give diagnostic criteria, if applicable	7
Data sources / measurement	#8	For each variable of interest give sources of data and details of methods of assessment (measurement). Describe comparability of assessment methods if there is more than one group. Give information separately for for exposed and unexposed groups if applicable.	7
Bias	#9	Describe any efforts to address potential sources of bias	6
Study size	#10	Explain how the study size was arrived at	5
Quantitative variables	#11	Explain how quantitative variables were handled in the analyses. If applicable, describe which groupings were chosen, and why	7
Statistical methods	#12a	Describe all statistical methods, including those used to control for confounding	
5			
Statistical methods	#12b	Describe any methods used to examine subgroups and interactions	8
Statistical methods	#12c	Explain how missing data were addressed	8
Statistical methods	#12d	If applicable, explain how loss to follow-up was addressed	6
Statistical methods	#12e	Describe any sensitivity analyses	
8			

Results

Participants	#13a	Report numbers of individuals at each stage of study—eg numbers potentially eligible, examined for eligibility, confirmed eligible,	10

For peer review only

		included in the study, completing follow-up, and analysed. Give information separately for for exposed and unexposed groups if applicable.	
Participants	#13b	Give reasons for non-participation at each stage	10
Participants	#13c	Consider use of a flow diagram	
10			
Descriptive data	#14a	Give characteristics of study participants (eg demographic, clinical, social) and information on exposures and potential confounders. Give information separately for exposed and unexposed groups if applicable.	10
Descriptive data	#14b	Indicate number of participants with missing data for each variable of interest	
10			
Descriptive data	#14c	Summarise follow-up time (eg, average and total amount)	
11			
Outcome data	#15	Report numbers of outcome events or summary measures over time. Give information separately for exposed and unexposed groups if applicable.	
11			
Main results	#16a	Give unadjusted estimates and, if applicable, confounder-adjusted estimates and their precision (eg, 95% confidence interval). Make clear which confounders were adjusted for and why they were included	12
Main results	#16b	Report category boundaries when continuous variables were categorized	12
Main results	#16c	If relevant, consider translating estimates of relative risk into absolute risk for a meaningful time period	
12			
Other analyses	#17	Report other analyses done—eg analyses of subgroups and interactions, and sensitivity analyses	14
**Discussion**			
Key results	#18	Summarise key results with reference to study objectives	23

For peer review only

Limitations	#19	Discuss limitations of the study, taking into account sources of potential bias or imprecision. Discuss both direction and magnitude of any potential bias.	24
Interpretation	#20	Give a cautious overall interpretation considering objectives, limitations, multiplicity of analyses, results from similar studies, and other relevant evidence.	25
Generalisability	#21	Discuss the generalisability (external validity) of the study results	25
**Other Information**			
Funding	#22	Give the source of funding and the role of the funders for the present study and, if applicable, for the original study on which the present article is based	27

The STROBE checklist is distributed under the terms of the Creative Commons Attribution License CC-BY. This checklist was completed on 26. April 2022 using https://www.goodreports.org/, a tool made by the EQUATOR Network in collaboration with Penelope.ai

For peer review only

BMJ Open

Sodium channel-inhibiting drugs and cancer-specific survival: a population-based study of electronic primary care data

Journal:	*BMJ Open*
Manuscript ID	bmjopen-2022-064376.R1
Article Type:	Original research
Date Submitted by the Author:	18-Jan-2023
Complete List of Authors:	Fairhurst, Caroline; University of York, Health Sciences<br>Martin, Fabiola; University of Queensland<br>Watt, Ian; University of York, Health Sciences<br>Bland, Martin; University of York, Health Sciences<br>Doran, Tim; University of York, Health Sciences<br>Brackenbury, William; University of York, Department of Biology
**Primary Subject Heading**:	Oncology
Secondary Subject Heading:	Epidemiology, Pharmacology and therapeutics, Neurology
Keywords:	Epilepsy < NEUROLOGY, ONCOLOGY, Breast tumours < ONCOLOGY, CLINICAL PHARMACOLOGY

SCHOLARONE™
Manuscripts

I, the Submitting Author has the right to grant and does grant on behalf of all authors of the Work (as defined in the below author licence), an exclusive licence and/or a non-exclusive licence for contributions from authors who are: i) UK Crown employees; ii) where BMJ has agreed a CC-BY licence shall apply, and/or iii) in accordance with the terms applicable for US Federal Government officers or employees acting as part of their official duties; on a worldwide, perpetual, irrevocable, royalty-free basis to BMJ Publishing Group Ltd ("BMJ") its licensees and where the relevant Journal is co-owned by BMJ to the co-owners of the Journal, to publish the Work in this journal and any other BMJ products and to exploit all rights, as set out in our licence.

The Submitting Author accepts and understands that any supply made under these terms is made by BMJ to the Submitting Author unless you are acting as an employee on behalf of your employer or a postgraduate student of an affiliated institution which is paying any applicable article publishing charge ("APC") for Open Access articles. Where the Submitting Author wishes to make the Work available on an Open Access basis (and intends to pay the relevant APC), the terms of reuse of such Open Access shall be governed by a Creative Commons licence – details of these licences and which Creative Commons licence will apply to this Work are set out in our licence referred to above.

Other than as permitted in any relevant BMJ Author's Self Archiving Policies, I confirm this Work has not been accepted for publication elsewhere, is not being considered for publication elsewhere and does not duplicate material already published. I confirm all authors consent to publication of this Work and authorise the granting of this licence.

For peer review only

Sodium channel-inhibiting drugs and cancer-specific survival: a population-based study of electronic primary care data

Caroline Fairhurst[1], Fabiola Martin[2], Ian Watt[1], Martin Bland[1], Tim Doran[1], William J. Brackenbury[3] (0000-0001-6882-3351)

[1]Department of Health Sciences, University of York, York, UK

[2]School of Public Health, University of Queensland, QLD, Australia

[3]York Biomedical Research Institute, Department of Biology, University of York, York, UK

*Correspondence to:

William J. Brackenbury, York Biomedical Research Institute, Department of Biology, University of York, Heslington, York, YO10 5DD, UK

E-mail: william.brackenbury@york.ac.uk

Telephone: +44 (0) 1904 328 284

Word count: 4518

Keywords: Anticonvulsants / Cancer / Epilepsy / Sodium channels

For peer review only

ABSTRACT

Objectives: Antiepileptic and antiarrhythmic drugs inhibit voltage-gated sodium (Na^{+}) channels (VGSCs), and preclinical studies show that these medications reduce tumour growth, invasion and metastasis. We investigated the association between VGSC inhibitor use and survival in breast, bowel and prostate cancer patients.

Design: Retrospective cohort study.

Setting: Individual electronic primary healthcare records extracted from the Clinical Practice Research Datalink (CPRD).

Participants: Records for 132,996 patients with a diagnosis of breast, bowel or prostate cancer.

Outcome measures: Adjusted Cox proportional hazards regression was used to analyse cancer-specific survival associated with exposure to VGSC inhibitors. Exposure to non-VGSC-inhibiting antiepileptic medication and other non-VGSC blockers were also considered. Drug exposure was treated as a time-varying covariate to account for immortal time bias.

Results: During 1,002,225 person-years of follow-up, there were 42,037 cancer-specific deaths. 53,724 (40.4%) cancer patients had at least one prescription for a VGSC inhibitor of interest. Increased risk of cancer-specific mortality was associated with exposure to this group of drugs (HR 1.59, 95% CI 1.56-1.63, p<0.001). This applied to VGSC-inhibiting tricyclic antidepressants (HR 1.61, 95% CI 1.50-1.65, p<0.001), local anaesthetics (HR 1.49, 95% CI 1.43-1.55, p<0.001) and anticonvulsants (HR 1.40, 95% CI 1.34-1.48, p<0.001), and persisted in sensitivity analyses. In contrast, exposure to VGSC-inhibiting Class 1c and 1d antiarrhythmics was associated with significantly improved cancer-specific survival (HR 0.75, 95% CI 0.64-0.88, p<0.001 and HR 0.54, 95% CI 0.33-0.88, p=0.01, respectively).

For peer review only

Conclusions: Association between VGSC inhibitor use and mortality in cancer patients varies according to indication. Exposure to VGSC-inhibiting antiarrhythmics, but not anticonvulsants, supports findings from preclinical data, with improved survival. However, additional confounding factors may underlie these associations, highlighting the need for further study.

Strengths and limitations of this study

- Primary care research data with large sample size and statistical power.
- Drug exposure is treated as a time-varying covariate to account for immortal time bias.
- No direct information on metastasis as an outcome.
- Drug exposure data are based on prescriptions.

For peer review only

For peer review only - http://bmjopen.bmj.com/site/about/guidelines.xhtml

INTRODUCTION

Metastatic disease is the leading cause of death from solid tumours (1), and there is an enduring need to identify new antimetastatic targets and therapies (2). One approach is to repurpose existing drugs used in the management of other conditions. In particular, ion channel blockers have been proposed as novel agents to treat cancer, including metastatic disease (3). However, no such agent has yet been progressed through to clinical use.

Voltage-gated sodium (Na^+) channels (VGSCs) are expressed on electrically excitable cells including neurons and muscle cells, where they regulate action potential firing (4). VGSC-inhibiting drugs are prescribed for a range of excitability-related conditions, including epilepsy, pain and cardiac arrhythmia (5,6). VGSCs are also widely expressed on malignant cells from a range of cancers, where they regulate Na^+ handling, pH buffering and the plasma membrane potential, promoting proliferation, migration, invasion and metastasis (7–12). Numerous preclinical studies have shown that VGSC-inhibiting medications can reduce tumour growth, invasion and metastasis (13–21). Although some antiepileptic drugs have been tested in clinical trials (22,23), their effect on VGSC activity in patient tumours has not been investigated. Several observational cohort studies have shown reduced cancer incidence (24,25) and risk of recurrence (26–28) in patients prescribed VGSC-inhibiting medications. In contrast, we have previously reported that exposure to VGSC-inhibiting medication was associated with reduced overall survival in cancer patients in a retrospective analysis (29). However, we were unable to control for epilepsy diagnosis, which is independently associated with increased all-cause mortality (30). In the present study, we conducted a retrospective cohort study using primary care data from the Clinical Practice Research Datalink (CPRD) in order to test the hypothesis that exposure to VGSC inhibitors prolongs cancer-specific survival. We controlled for epilepsy diagnosis and timing of exposure to VGSC-inhibiting drugs and considered other antiepileptic medications.

For peer review only - http://bmjopen.bmj.com/site/about/guidelines.xhtml

METHODS

Patient data

The study protocol has been published previously (31). Several additional analyses were performed as detailed below. Primary care records for patients with a first diagnosis of any cancer between 2001 and 2011 and aged 25 years or over at diagnosis were obtained from the CPRD GOLD and Aurum databases. CPRD contains anonymised individual patient data on morbidity, mortality, prescribing, treatment and referrals collected from primary care practices in England. Data were extracted in August 2019. Within this dataset, we identified patients with a recorded medical code for breast, bowel or prostate cancer (hereafter referred to as the *index* cancers). The role of VGSCs has been extensively studied in these three types of cancer, and they are among the most common in the UK (12,31). Prescription data were interrogated to identify patients with a recorded prescription for VGSC-inhibiting medications (including anticonvulsants, local anaesthetics, antiarrhythmics and certain tricyclic antidepressants; Supplementary Table 1). We also identified patients with a recorded prescription for non-VGSC-inhibiting anticonvulsants (e.g. gabapentinoids, benzodiazepines) and medications targeting other (non-voltage-gated) Na^+ channels (e.g. the epithelial Na^+ channel [ENaC]), at any time (Supplementary Table 1). We searched diagnostic codes to identify patients with a recorded VGSC inhibitor indication (epilepsy, cardiac arrhythmia, amyotrophic lateral sclerosis (ALS), and neuropathic pain) (5,6).

Statistical analysis

Time-dependent Cox proportional hazards regression was used to analyse survival time from cancer diagnosis associated with exposure to the medication group of interest, and all models were adjusted for type of index cancer, sex and age at diagnosis (age+age^2), unless otherwise stated. Right censoring occurred if the patient died of any other cause, was still alive at the point the data were extracted or the patient transferred out of a CPRD GP practice.

For peer review only - http://bmjopen.bmj.com/site/about/guidelines.xhtml

To account for potential immortal time bias (32) in patients whose prescriptions only begin after their cancer diagnosis, drug exposure status was considered as a time-dependent covariate in the following three ways:

Scenario 1: all person-time of follow-up from diagnosis to death/censor was classified as exposed for patients who have at least one prescription of interest before their diagnosis; while for those who only have prescriptions after their diagnosis, their survival time was classified as unexposed between diagnosis and date of first prescription, and as exposed thereafter.

Scenario 2: person-time was considered as unexposed until the date of the first prescription and as exposed thereafter for patients whose prescriptions either: (i) start before diagnosis and extend after; or (ii) start after diagnosis. This differs from Scenario 1 in that, for patients whose first and last prescriptions are before their cancer diagnosis, their survival time is treated as exposed in Scenario 1 and unexposed in this scenario.

Scenario 3: person-time was considered as unexposed until the date of the first prescription *following* the date of cancer diagnosis and as exposed thereafter. This differs from Scenario 2 in that, for patients whose prescriptions of interest start before diagnosis and extend after, the time between diagnosis and the first prescription after diagnosis is considered exposed in Scenario 2 and unexposed in this scenario.

In all scenarios, all person-time of follow-up for patients who have never had a recorded prescription of interest was classified as unexposed. A depiction of these scenarios is presented in the published protocol (31).

Multivariable-adjusted hazard ratios (HR) are presented with a 95% confidence interval (CI) and p value. Analyses were conducted in Stata v15 (33), using two-sided statistical tests at the 5% significance level.

For peer review only - http://bmjopen.bmj.com/site/about/guidelines.xhtml

Survival graphs were produced using the Simon–Makuch method, which is an alternative to Kaplan–Meier that appropriately accounts for the time-varying covariate of exposure (34).

Patient characteristics

Patient characteristics, stratified by 'ever' and 'never' exposure to a VGSC-inhibiting medication, are summarised using mean and standard deviation (SD) for continuous data and count and percentage for categorical variables, and compared using a t- or χ^2-test as appropriate. Amide and ester local anaesthetic injections were not included within the definition of 'exposed', due to their short-term use and transient effect.

Characteristics of the 'ever' exposed group stratified by timing of drug exposure relative to their cancer diagnosis (before only, before and after, and after only) are also presented, including length of drug exposure and most commonly prescribed drug class. Extent of drug exposure was estimated by calculating the time between the first and last recorded prescription, plus a number of weeks (the average interval between all prescriptions) to account for the time patients were assumed to be taking their final recorded prescription. Based on this, patients were classified into short (< 6 months), or long (≥ 6 months). exposure groups. Those who had two or more prescriptions relating to one of the VGSC-inhibiting drugs within two years before the date of the cancer diagnosis, including at least one within six months before, were classified as having recent (to cancer diagnosis) exposure. Alternative medications were summarised for patients with a recorded diagnostic code for an indication for a VGSC-inhibiting drug (epilepsy, neuropathic pain, cardiac arrhythmia, ALS) who did not have a recorded prescription for a VGSC-inhibiting drug.

Primary analysis

The primary analysis investigated cancer-specific mortality (any cancer as the underlying cause) associated with drug exposure, treated as a time-varying covariate according to the three scenarios described above, using adjusted Cox proportional hazards regression models.

For peer review only - http://bmjopen.bmj.com/site/about/guidelines.xhtml

Sensitivity analyses

The primary analyses were repeated with the Cox models additionally adjusted for: ethnicity, BMI, physical activity, smoking status, alcohol consumption, Charlson comorbidity index (CCI) score, presence of an indication for VGSC-inhibiting medication and area-level social deprivation, using the Index of Multiple Deprivation (IMD) in twentiles (1=least deprived to 20=most deprived) based on patient postcode (2010). In further sensitivity analyses, missing values for the confounding factors (previously included in a 'not recorded' category) were imputed using multiple imputation and the analysis models rerun.

In addition, the primary analysis was repeated using competing-risks regression, according to the method of Fine and Gray and implemented using the *stcrreg* command in Stata (35), with death by any other cause but cancer as the competing risk, and also after introducing a lag such that patients were not considered as exposed until three months after drug use. This excludes prescriptions shortly before death and therefore minimises potential reverse causation (36).

Secondary analyses

The primary analyses were repeated stratified by index cancer diagnosis (not adjusting for index cancer, and also removing sex as a covariate for prostate cancer analysis as all patients were male, and for breast cancer as nearly all patients were female) and comparing time to: i) death from index cancer (underlying or contributory cause); ii) death from any cancer (underlying or contributory cause); and iii) all-cause mortality. The primary analyses were also repeated by including in the 'ever' exposed group, in turn, only patients who had: i) ever used; ii) had recent exposure (according to definition as above) to; or iii) whose most commonly prescribed VGSC inhibitor (not including local anaesthetic injections) was tricyclic antidepressants, anticonvulsants, and antiarrhythmics. Other drug classes were not considered due to insufficient numbers in these groups.

Vaughan Williams classification of antiarrhythmics

For peer review only - http://bmjopen.bmj.com/site/about/guidelines.xhtml

We repeated the primary analyses considering exposure to VGSC-inhibiting antiarrhythmics subdivided according to the updated Vaughan Williams classification (Supplementary Table 1) (37,38). Exposure to different classes of antiarrhythmic medications was assessed depending on whether the patient's use of the drugs was defined as: (i) ever use; (ii) recent use; or (iii) their most common VGSC inhibitor prescription.

Amide or ester local anaesthetic injections

We repeated the time-dependent analysis (Scenario 3 only since 1 and 2 are not applicable here) including only those patients whose VGSC-inhibiting drug prescriptions were solely for amide or ester local anaesthetic injections (Supplementary Table 1) following their diagnosis in the exposed group, since there is evidence that local anaesthetics used perioperatively can be associated with reduced tumour recurrence (12).

Non-VGSC-targeting antiepileptic medication

We repeated the primary analyses considering exposure to non-VGSC-targeting antiepileptic medications, and blockers of other (non voltage-gated) Na^+ channels (Supplementary Table 1).

Ethics approval

This study was performed following ethics approval from the Department of Biology Ethics Committee, University of York (WB201909).

Patient and public involvement

None.

RESULTS

Population characteristics

The CPRD dataset contained records for 515,987 patients from 1,057 GP practices, including 132,996 (25.8%) patients with a diagnostic code for breast (n=59,528), prostate

For peer review only - http://bmjopen.bmj.com/site/about/guidelines.xhtml

(n=50,601) or bowel (n=22,867) cancer recorded during at least one of their GP consultations. Of the 132,996 index cancer patients, 79,164 (59.5%) had at least one prescription, at any time, for a specified VGSC-inhibiting drug; tricyclic antidepressant was the most commonly prescribed VGSC-inhibiting drug group for the majority of exposed patients (n=33,905, 42.8%), followed by amide local anaesthetics (n=30,091, 38.0%). For a third of these 79,164 patients (n=25,440, 32.1%), their only exposure to a VGSC-inhibiting drug was to amide or ester local anaesthetics. These patients were classified as unexposed for most of the described analyses, due to the short-term exposure, so 53,724 (40.4%) patients were observed to have had at least some exposure to a VGSC inhibitor of interest, before and/or after cancer diagnosis, and 79,272 (59.6%) were not (Supplementary Table 2). Stratified by index cancer, the proportion of 'ever' exposed patients was: breast 59.5%; bowel 54.7%; and prostate 61.7%.

Between the 'ever' and 'never' exposure groups, formal comparisons indicated statistically significant differences in all observed characteristics, even where differences were very small such as in the Charlson Comorbidity Index (mean 6.1 in the 'ever' exposed group and 5.9 in the unexposed group), which is likely to be an artefact of the large sample size (Supplementary Table 2). On visual inspection, the two exposure groups appear similar for most patient characteristics, including age, but there was a notable imbalance in sex, with a greater proportion of females in the 'ever' exposed group than in the unexposed group. There were expected differences in the proportions of patients with an indication for treatment with a VGSC inhibitor; for example, 3.6% of the 'ever' exposed group had a diagnosis of epilepsy, compared with 0.6% of the unexposed group.

Within the 'ever' exposed group, 14,157 patients (26.4%) only had prescriptions of interest dated before a cancer diagnosis, 17,264 (32.1%) had prescriptions dated both before and after diagnosis, and 22,303 (41.5%) only had prescriptions dated after diagnosis (Supplementary Table 3). For patients who initiated VGSC inhibitors after their cancer

For peer review only - http://bmjopen.bmj.com/site/about/guidelines.xhtml

diagnosis, the mean interval between diagnosis and first recorded prescription was 4.0 years (SD 3.5, median 3.0, range 1 day to 18.2 years).

For the subset of patients with a recorded diagnosis of an indication for VGSC-inhibiting medication in their medical records who did not have a recorded prescription for a VGSC-inhibiting drug (n=16,048), the most common prescriptions were for angiotensin-converting enzyme inhibitors (727,736 prescriptions among 9,887 (61.6%) patients), lipid-regulating drugs (647,200 prescriptions among 8,099 (50.5% patients), antiplatelet drugs (622,772 prescriptions among 10,602 (66.1%) patients), beta-adrenoceptor blocking drugs (515,888 prescriptions among 7,798 (48.6%) patients) and voltage-gated calcium channel blockers (503,847 prescriptions among 8,044 (50.1%) patients). These proportions were very similar for the subset of patients with a recorded diagnosis of an indication for VGSC-inhibiting medication in their medical records who did have a recorded prescription for a VGSC-inhibiting drug (n=18,744), except that a slightly higher proportion of these patients had a prescription for a beta-blocker (54.9%).

The maximum follow-up from diagnosis was 18.6 years (median 7.9 years). During 1,002,225 person-years of follow-up, there were 66,960 deaths from any cause (Supplementary Table 4). A similar proportion of deaths from any cause were recorded in the data for the two groups ('ever' exposed 48.4%, unexposed 51.6%), and of deaths with any cancer listed as the underlying cause (primary outcome, total n=42,037; 'ever' exposed 29.7%, unexposed 32.9%) or as at least a contributory cause (n=32,725; 'ever' exposed 34.6%, unexposed 38.5%) (Supplementary Table 4).

Primary, sensitivity and secondary analyses

The main text focuses on results from analyses relating to Scenario 3, as this most closely matches the design of relevant preclinical studies (13,14), but all results are presented in the tables. In the primary analysis, we considered the relationship between all VGSC inhibitors (excluding local anaesthetics) and cancer-specific survival across all three index cancer

For peer review only - http://bmjopen.bmj.com/site/about/guidelines.xhtml

types (breast, bowel and prostate) combined. Exposure to VGSC inhibitors was associated with a statistically significant increased risk of death from cancer (HR 1.59, 95% CI 1.56 to 1.63, $p<0.001$; Table 1; Figure 1). The HR increased in the sensitivity analysis additionally adjusted for ethnicity, BMI, physical activity, smoking status, alcohol consumption, IMD, CCI score, and presence of a VGSC-inhibitor indication (1.65, 95% CI 1.62 to 1.69), and in the competing-risks analysis (1.65, 95% CI 1.53 to 1.78), but was similar after missing covariate data were imputed albeit with a wider confidence interval (1.60, 95% CI 1.49 to 1.72). A smaller but still significant effect was observed in the analysis that utilised a lag of three months to discount drug use shortly before death (HR 1.37, 95% CI 1.34 to 1.41, $p<0.001$; Table 1).

For peer review only - http://bmjopen.bmj.com/site/about/guidelines.xhtml

Table 1. Estimates of the relationship between exposure to VGSC inhibitors and cancer specific mortality—primary and sensitivity analyses

Cancer-specific mortality (underlying cause)	HR (95% CI)	p-value
**Primary analysis**		
Scenario 1	1.33 (1.31, 1.36)	<0.001
Scenario 2	1.31 (1.28, 1.34)	<0.001
Scenario 3	1.59 (1.56, 1.63)	<0.001
**Sensitivity analyses 1[a]**		
Scenario 1	1.42 (1.39, 1.45)	<0.001
Scenario 2	1.38 (1.34, 1.41)	<0.001
Scenario 3	1.65 (1.62, 1.69)	<0.001
**Sensitivity analyses 2[b]**		
Scenario 1	1.34 (1.26, 1.43)	<0.001
Scenario 2	1.31 (1.22, 1.41)	<0.001
Scenario 3	1.60 (1.49, 1.72)	<0.001
**Sensitivity analyses 3[c]**		
Scenario 1	1.34 (1.26, 1.43)	<0.001
Scenario 2	1.35 (1.25, 1.45)	<0.001
Scenario 3	1.65 (1.53, 1.78)	<0.001
**Sensitivity analyses 4[d]**		
Scenario 1	1.20 (1.18, 1.23)	<0.001
Scenario 2	1.17 (1.14, 1.20)	<0.001
Scenario 3	1.37 (1.34, 1.41)	<0.001

[a]primary analyses additionally adjusted for ethnicity, BMI, physical activity, smoking status, alcohol consumption, CCI score, IMD score and presence of: epilepsy; cardiac arrhythmias; ALS; neuropathic pain/painful neuropathy.
[b]sensitivity analyses 1 repeated after unknown values of ethnicity, BMI, physical activity, smoking status, IMD and alcohol consumption imputed using multiple imputation.
[c]competing-risks regression using *stcrreg* command in Stata adjusting for exposure group, type of cancer, sex, age and age^2, with death by any other cause but cancer as the competing risk.
[d]primary analysis repeated after introducing a three month lag to exposure.

For peer review only - http://bmjopen.bmj.com/site/about/guidelines.xhtml

In secondary analyses, we stratified by cancer type; there was a statistically significantly ($p<0.001$) increased mortality rate associated with exposure to VGSC-inhibiting medication across all three cancers, HR (95% CI) for: breast 1.49 (1.43 to 1.54); prostate 1.65 (1.60 to 1.71); and bowel 1.64 (1.57 to 1.71) (Table 2). There was a similar relationship for the outcomes of time to death from specific index cancer (HR 1.58, 95% CI 1.55 to 1.62), cancer as an underlying or contributory cause (1.56, 1.53 to 1.60) and all-cause mortality (1.50, 1.48 to 1.53) (Table 2).

For peer review only

For peer review only - http://bmjopen.bmj.com/site/about/guidelines.xhtml

Table 2. Estimates of the relationship between exposure to VGSC inhibitors and mortality—secondary analyses

**Secondary analyses**	**HR (95% CI)**	**p-value**
Primary analyses by type of cancer		
Breast		
Scenario 1	1.27 (1.23, 1.32)	<0.001
Scenario 2	1.22 (1.18, 1.27)	<0.001
Scenario 3	1.49 (1.43, 1.54)	<0.001
Prostate		
Scenario 1	1.38 (1.33, 1.42)	<0.001
Scenario 2	1.42 (1.37, 1.47)	<0.001
Scenario 3	1.65 (1.60, 1.71)	<0.001
Bowel		
Scenario 1	1.34 (1.29, 1.40)	<0.001
Scenario 2	1.26 (1.21, 1.32)	<0.001
Scenario 3	1.64 (1.57, 1.71)	<0.001
Death from index cancer (underlying or contributory cause)		
Scenario 1	1.33 (1.31, 1.36)	<0.001
Scenario 2	1.30 (1.27, 1.32)	<0.001
Scenario 3	1.58 (1.55, 1.62)	<0.001
Cancer-specific mortality (underlying or contributory cause)		
Scenario 1	1.33 (1.31, 1.36)	<0.001
Scenario 2	1.29 (1.27, 1.32)	<0.001
Scenario 3	1.56 (1.53, 1.60)	<0.001
All-cause mortality		
Scenario 1	1.34 (1.32, 1.36)	<0.001
Scenario 2	1.28 (1.26, 1.30)	<0.001
Scenario 3	1.50 (1.48, 1.53)	<0.001

For peer review only - http://bmjopen.bmj.com/site/about/guidelines.xhtml

For peer review only

VGSC-inhibiting anticonvulsants and tricyclic antidepressants

Among patients with exposure to anticonvulsants (ever use n=6,391), VGSC inhibitor use was associated with significantly increased risk of death from cancer (HR 1.40, 95% CI 1.34 to 1.48, p<0.001; Supplementary Figure 1A; Table 3). A higher HR was observed among those for whom anticonvulsants were the most frequent prescription for a VGSC inhibitor (1.62, 95% CI 1.53 to 1.72), but lower for recent use (1.11, 95% CI 1.02 to 1.21). Among patients with exposure to tricyclic antidepressants (ever use n=42,715), VGSC inhibitor use was similarly associated with significantly increased risk of death from cancer (HR 1.61, 95% CI 1.5 to 1.65, p<0.001; Supplementary Figure 1B; Table 3); again, a higher HR was associated with tricyclic antidepressants being the most frequent prescription for a VGSC inhibitor (1.67, 95% CI .63 to 1.71), but lower (and non-statistically significant) for recent use (0.98, 95% CI 0.93 to 1.04, p=0.59).

For peer review only - http://bmjopen.bmj.com/site/about/guidelines.xhtml

Table 3. Estimates of the relationship between exposure to VGSC-inhibiting drugs, subdivided by type, and cancer-specific mortality

VGSC inhibitor drug group	Exposed* (n=53724), n (%)	HR (95% CI) p-value Scenario 1	HR (95% CI) p-value Scenario 2	HR (95% CI) p-value Scenario 3
Ever use				
Antiarrhythmic	15538 (28.9)	0.91 (0.88, 0.95) p<0.001	0.84 (0.80, 0.87) p<0.001	1.02 (0.98, 1.06) p=0.34
Anticonvulsant	6391 (11.9)	1.19 (1.14, 1.24) p<0.001	1.17 (1.12, 1.23) p<0.001	1.40 (1.34, 1.48) p<0.001
Tricyclic antidepressant	42715 (79.5)	1.32 (1.29, 1.35) p<0.001	1.30 (1.27, 1.33) p<0.001	1.61 (1.57, 1.65) p<0.001
Recent use				
Antiarrhythmic	2807 (5.2)	0.95 (0.89, 1.01) p=0.12	0.87 (0.81, 0.93) p<0.001	0.92 (0.86, 0.99) p=0.03
Anticonvulsant	1656 (3.1)	1.14 (1.05, 1.24) p<0.001	1.05 (0.96, 1.15) p=0.27	1.11 (1.02, 1.21) p=0.02
Tricyclic antidepressant	5408 (10.1)	1.01 (0.96, 1.06) p=0.76	0.90 (0.85, 0.95) p<0.001	0.98 (0.93, 1.04) p=0.59
Most common VGSC inhibitor prescription				
Antiarrhythmic	11032 (20.5)	0.94 (0.91, 0.98) p<0.001	0.87 (0.83, 0.91) p<0.001	1.00 (0.95, 1.05) p=0.94
Anticonvulsant	4062 (7.6)	1.41 (1.34, 1.48) p<0.001	1.45 (1.36, 1.53) p<0.001	1.62 (1.53, 1.72) p<0.001
Tricyclic antidepressant	38600 (71.9)	1.36 (1.33, 1.39) p<0.001	1.37 (1.33, 1.40) p<0.001	1.67 (1.63, 1.71) p<0.001

*Figures in this column relate to the number of patients recorded as having at least some follow-up time considered as exposed to the drug class of interest in Scenario 1 for each definition (ever use, recent use, most common), as a percentage of the whole 'ever' exposed group. The number of patients with any person-time of follow-up considered as exposed for each drug class will be lower in Scenario 2, and fewer still in Scenario 3.

For peer review only - http://bmjopen.bmj.com/site/about/guidelines.xhtml

A total of 12,140 patients received VGSC-inhibiting drug prescriptions solely in the form of amide or ester local anaesthetic injections following their cancer diagnosis, of which 3,656 (30.1%) died with (any) cancer as the underlying cause. Exposure to these injections was associated with a statistically significantly increased risk of death from any cancer (HR 1.49, 95% CI 1.43 to 1.55, p<0.001).

Class 1-3 antiarrhythmics

In contrast to the VGSC-inhibiting anticonvulsants and tricyclic antidepressants, exposure to VGSC-inhibiting antiarrhythmic drugs was associated with decreased risk of cancer-specific mortality (recent use HR 0.92, 95% CI 0.86 to 0.99, p=0.03), or no difference (Supplementary Figure 1C; Table 3). In exploratory analyses, these drugs were separated into their Vaughan Williams classes (Table 4, Supplementary Figures 2A-2D) (37,38). Exposure to Class 1a antiarrhythmic drugs (n=188) had no impact on cancer-specific survival (ever use HR 1.05, 95% CI 0.76 to 1.46, p=0.77; Supplementary Figure 2A; Table 4). Exposure to Class 1b drugs (n=1088), some of which are also indicated as anticonvulsants (e.g. phenytoin), was associated with significantly reduced cancer-specific survival (ever use HR 2.06, 95% CI 1.88 to 2.26, p<0.001; Supplementary Figure 2B; Table 4). In contrast, exposure to Class 1c drugs (n=860) was associated with significantly improved cancer-specific survival (ever use HR 0.75, 95% CI 0.64 to 0.88, p<0.001; Supplementary Figure 2C; Table 4). The Class 1d drug ranolazine (n=165) was associated with significantly improved cancer-specific survival (ever use HR 0.54, 95% CI 0.33 to 0.88, p=0.01; Supplementary Figure 2D; Table 4). However, Class 2 drugs (beta blockers; n=11,643) were not associated with altered cancer-specific survival (ever use HR 0.99, 95% CI 0.94 to 1.04, p=0.70; Table 4). Finally, Class 3 drugs (which are also K^+ channel blockers; n=3532) also were not associated with altered cancer-specific survival, (ever use HR 1.06, 95% CI 0.98 to 1.13, p=0.14; Table 4).

For peer review only - http://bmjopen.bmj.com/site/about/guidelines.xhtml

Table 4. Estimates of the relationship between exposure to antiarrhythmic drugs, subdivided by Vaughan Williams classification, and cancer-specific mortality

Vaughan Williams drug groups	Exposed[a] (n=53724) n (%)	HR (95% CI) p-value Scenario 1	HR (95% CI) p-value Scenario 2	HR (95% CI) p-value Scenario 3
Ever use				
1a Fast VGSC block, K^+ channel block	188 (0.3)	0.94 (0.73, 1.21) p=0.64	0.88 (0.64, 1.22) p=0.45	1.05 (0.76, 1.46) p=0.77
1b[b] VGSC block, fast association/ disassociation	1088 (2.0)	1.82 (1.67, 1.99) p<0.001	1.84 (1.68, 2.02) p<0.001	2.06 (1.88, 2.26) p<0.001
1c VGSC block, slow association/ disassociation	860 (1.6)	0.73 (0.64, 0.84) p<0.001	0.67 (0.57, 0.78) p<0.001	0.75 (0.64, 0.88) p<0.001
1d Persistent current block	165 (0.3)	0.41 (0.25, 0.68) p<0.001	0.42 (0.26, 0.68) p<0.001	0.54 (0.33, 0.88) p=0.01
2 Beta adrenergic block	11643 (21.7)	0.87 (0.84, 0.91) p<0.001	0.79 (0.75, 0.83) p<0.001	0.99 (0.94, 1.04) p=0.70
3 K^+ channel block	3532 (6.6)	0.98 (0.92, 1.04) p=0.50	0.92 (0.85, 0.98) p=0.02	1.06 (0.98, 1.13) p=0.14
Recent use				
1a	45 (0.1)	1.17 (0.73, 1.88) p=0.52	1.00 (0.59, 1.69) p=1.00	1.06 (0.63, 1.79) p=0.83
1b[a]	429 (0.8)	1.22 (1.05, 1.42) p=0.01	1.15 (0.98, 1.34) p=0.10	1.21 (1.03, 1.42) p=0.02
1c	298 (0.6)	0.82 (0.66, 1.01) p=0.06	0.80 (0.65, 0.99) p=0.04	0.84 (0.68, 1.04) p=0.11
1d	4 (0.0)	-	-	-
2	1752 (3.3)	0.92 (0.84, 1.00) p=0.06	0.84 (0.77, 0.92) p<0.001	0.89 (0.81, 0.98) p=0.01
3	738 (1.4)	1.03 (0.92, 1.16) p=0.59	0.94 (0.83, 1.07) p=0.37	1.01 (0.89, 1.14) p=0.91
Most common VGSC inhibitor prescription				
1a	107 (0.2)	1.06 (0.77, 1.45) p=0.71	0.97 (0.62, 1.52) p=0.89	1.10 (0.70, 1.73) p=0.67

For peer review only - http://bmjopen.bmj.com/site/about/guidelines.xhtml

1b[a]	756 (1.4)	1.95 (1.76, 2.16) p<0.001	1.97 (1.76, 2.19) p<0.001	2.16 (1.94, 2.41) p<0.001
1c	632 (1.2)	0.76 (0.65, 0.89) p<0.001	0.71 (0.59, 0.86) p<0.001	0.78 (0.65, 0.94) p=0.01
1d	126 (0.2)	0.43 (0.24, 0.78) p=0.01	0.44 (0.24, 0.79) p=0.01	0.57 (0.31, 1.02) p=0.06
2	8025 (14.9)	0.92 (0.88, 0.96) p<0.001	0.84 (0.79, 0.89) p<0.001	1.01 (0.95, 1.07) p=0.84
3	2786 (5.2)	1.03 (0.96, 1.10) p=0.46	0.96 (0.89, 1.04) p=0.34	1.08 (1.00, 1.17) p=0.06

[a]Figures in this column relate to the number of patients recorded as having at least some follow-up time considered as exposed to the drug group of interest in Scenario 1 for each definition (ever use, recent use, most common), as a percentage of the whole 'ever' exposed group. The number of patients with any person-time of follow-up considered as exposed for each drug group will be lower in Scenario 2, and fewer still in Scenario 3.
[b]Excluding lidocaine, which is commonly prescribed as a local anaesthetic.

For peer review only

Non-VGSC-targeting antiepileptic medications and other Na^+ channel blockers

To investigate whether the reduced cancer-specific survival of patients exposed to VGSC-inhibiting anticonvulsants is attributable to their Na^+ current-inhibiting action, we considered the impact of two other drug groups: (1) anticonvulsants that do not target VGSCs; and (2) drugs that target other types of Na^+ channels, independent of VGSCs. A third (n=46,017, 34.6%) of patients had a prescription for a non-VGSC-targeting anticonvulsant, and 7% (n=9,256) for a non-VGSC-targeting Na^+ channel blocker (Supplementary Table 1). For both drug groups, there was a higher proportion of deaths (from any cause) among those exposed than among those not exposed, and this was true when cancer was considered among the causes of death (Supplementary Table 4). Among those who died, patients exposed to a non-VGSC-targeting antiepileptic medication were more likely to die with any cancer as an underlying cause than unexposed patients (71.1% versus 57.7%); whereas patients exposed to a non-VGSC-targeting Na^+ channel blocker were less likely (51.7% versus 64.1%). Exposure to both drug groups was associated with increased risk of cancer-specific mortality (HR 4.60, 95% CI 4.51 to 4.70, $p<0.001$ for non-VGSC-inhibiting anticonvulsants; and 1.42, 95% CI 1.35 to 1.49, $p<0.001$ for non-VGSC-inhibiting Na^+ channel blockers; Supplementary Figure 3A, B; Table 5). Findings are presented by drug class in Supplementary Table 5.

For peer review only - http://bmjopen.bmj.com/site/about/guidelines.xhtml

Table 5. Estimates of the relationship between exposure to non-VGSC-inhibiting anticonvulsants, non-VGSC-inhibiting Na^+ channel blockers and cancer-specific mortality

Cancer-specific mortality (underlying cause)	Non-VGSC-inhibiting anticonvulsant		Non-VGSC-inhibiting Na^+ channel blocker	
	HR (95% CI)	p-value	HR (95% CI)	p-value
Scenario 1	2.98 (2.92, 3.04)	<0.001	1.25 (1.21, 1.30)	<0.001
Scenario 2	3.60 (3.53, 3.68)	<0.001	1.32 (1.26, 1.39)	<0.001
Scenario 3	4.60 (4.51, 4.70)	<0.001	1.42 (1.35, 1.49)	<0.001

For peer review only

DISCUSSION

This study shows that exposure to VGSC-inhibiting drugs (anticonvulsants, local anaesthetics and tricyclic antidepressants) in breast, bowel and prostate cancer patients is associated with a statistically significant increased risk of death from cancer. This risk is elevated for patients who were exposed to this class of medication before, as well as after, their cancer diagnosis. In addition, both non-VGSC-targeting anticonvulsants and non-VGSC-targeting Na^+ channel blockers are associated with significantly increased risk of death from cancer. Notably, the risk of death from cancer is approximately two times higher for non-VGSC-targeting vs. VGSC-inhibiting anticonvulsants. In contrast, VGSC-inhibiting antiarrhythmic medications display a different pattern, and are associated with moderately improved cancer-specific survival. When subdivided according to the updated Vaughan Williams classification, Class 1c and 1d VGSC inhibitors (which have slow receptor association/disassociation, producing persistent current block) are associated with significantly improved cancer-specific survival in several scenarios.

Strengths and weaknesses of the study

The study uses data from the CPRD, the largest prospectively collected primary care database in the UK containing information on causes of death, comorbidities, and drug exposure based on prescription data (39,40). We studied cancer-specific mortality in addition to overall mortality, and we controlled for other potentially confounding life-limiting indications for which VGSC-inhibiting medications are prescribed (5,6,31,41). A key limitation of observational studies of drug effects on survival is immortal time bias, where patients in the exposed group can enter an “immortal” period in the follow up time between index diagnosis and first prescription of the drug under study (42). We implemented a person-time approach to control for this issue, where exposure status was considered as a time-dependent covariate (31,42). However, this adjustment did not alter the overall conclusions. We also conducted analyses that added a lag of three months to exposure to minimise issues of reverse causation; again, conclusions were unchanged.

For peer review only - http://bmjopen.bmj.com/site/about/guidelines.xhtml

There are several important limitations to the study. Firstly, GP records, including diagnostic codes, covariate data and prescription information, may be incomplete or contain errors. Additionally, a prescription record does not account for non-adherence, and so exposure to the drugs of interest is inferred. Secondly, although the dataset was linked to causes of death, it was not linked to secondary care databases, including the National Cancer Data Repository (43), and so we did not have access to information on cancer stage, progression or treatment. Thirdly, although we were able to identify those cancer patients who had a diagnostic code for a confounding life-limiting indication, for example epilepsy, we had limited information on the severity of the conditions, which is linked to both medication use and survival. It is possible that additional uncontrolled confounding factors in the population may underlie the associations, for example cardiovascular complications (44,45), underscoring a key problem with such retrospective cohort studies. We also did not measure metastasis directly, so further work is required to establish why cancer patients exposed to these medications have altered survival.

Comparison with other studies

Our findings partially agree with our previous study showing that exposure to VGSC-inhibiting medications is associated with reduced overall survival of cancer patients (29,46). Refinements to the design of the current study, including adjustment for epilepsy diagnosis, and analysis of cancer-specific survival in addition to overall survival (31), did not alter this conclusion. However, subdividing VGSC inhibitors according to their primary indication revealed positive associations between exposure to antiarrhythmics (in particular Class 1c and 1d drugs) and cancer-specific survival. In addition, the current study showed for the first time that the negative association between anticonvulsant exposure and cancer-specific survival was greater for non-VGSC-targeting anticonvulsants than for VGSC-inhibiting anticonvulsants. A number of preclinical studies indicate that VGSC-inhibiting medications reduce survival, proliferation, migration, invasion and metastasis of cancer cells (13–15,47–50). These would support the hypothesis that such drugs may have value as anti-metastatic

For peer review only - http://bmjopen.bmj.com/site/about/guidelines.xhtml

agents. In addition, several clinical studies have shown valproate, another VGSC blocker, to have anti-tumour activity (23,51–53). However, this may, at least partially, be as a result of its action as a histone deacetylase inhibitor (15,54).

Implications for clinical practice

The disagreement between the preclinical observations and the primary care data presented here raises the possibility that any beneficial effect of VGSC-inhibiting medications on cancer progression may be masked by larger effects in the population. We previously postulated that estimation of a positive association may be affected by confounding by indication (29). VGSC-inhibiting medications are indicated primarily for epilepsy, but are also prescribed for other life-limiting conditions, including cardiac arrhythmias, ALS and neuropathic pain/painful neuropathy (5,6,31,41). Epilepsy patients have an elevated risk of death from all causes, including cancer, compared to the general population (standardised mortality ratio >2.2) (30,55,56), possibly due to a poorer general health and/or social status (44,57,58). Adjustment for comorbidities and social deprivation had no effect on the relationship between exposure and reduced survival. In addition, several VGSC-inhibiting antiepileptic drugs, including carbamazepine and phenytoin, can induce activity of the hepatic cytochrome P450 isoenzyme system, which in turn metabolises certain chemotherapeutic agents, including camptothecin analogues, methotrexate, taxanes, teniposide and vinca alkaloids (59,60). Some VGSC inhibitors, including phenytoin, have also been shown to impact on immune function (61). Alterations in bioavailability and efficacy of chemotherapeutic agents in the presence of VGSC inhibitors, as well as potential interactions with other treatments, should be studied further.

The observation that non-VGSC-targeting anticonvulsants were associated with worse survival than VGSC-inhibiting anticonvulsants raises the possibility that VGSC inhibition may indeed be beneficial in this cohort of cancer patients, thus indirectly supporting the preclinical hypothesis (9). Moreover, the improved cancer-specific survival of patients exposed to Class 1c and 1d antiarrhythmics, which preferentially target the persistent Na^+ current that is

For peer review only - http://bmjopen.bmj.com/site/about/guidelines.xhtml

responsible for VGSC-dependent metastatic behaviour in preclinical models (11,14,37), further supports the notion that inhibition of these channels may be beneficial in the clinical setting. However, we cannot exclude the possibility that other confounders may exist between patients within these subgroups, for example epilepsy or cardiac arrhythmia severity.

CONCLUSIONS

The unique positive association between antiarrhythmic drug prescriptions and improved survival may point to a specific beneficial effect of certain VGSC inhibitors with this indication, e.g. ranolazine (14,21), and warrants further investigation. These results should be replicated in a study with robust cancer stage data, and an appropriately designed and controlled prospective clinical trial to establish the effect of VGSC inhibition on tumour progression. Such a trial would separate possible uncontrolled confounding from cancer-specific mortality and could also exploit emerging novel pathophysiological biomarkers of disease progression, for example circulating tumour DNA and ^{23}Na-MRI.

ADDITIONAL INFORMATION

Acknowledgements

The authors acknowledge the Medical Research Council and the Wellcome Trust for funding this study.

Contributors

CF, TD and WB had the original idea for this study. CF conducted the analysis under supervision of TD and WB. CF, TD and WB wrote the draft of the manuscript. IW, FM and MB contributed to the development of the idea, the study design, interpretation of the findings and revising the manuscript. All authors approved the final submitted version of the manuscript.

For peer review only - http://bmjopen.bmj.com/site/about/guidelines.xhtml

Ethics approval

This study was performed following ethics approval from the Department of Biology Ethics Committee, University of York (WB201909). GPs do not seek individual patient consent when they share de-identified data with the CPRD (CPRD policy here: https://www.cprd.com/public). The study was performed in accordance with the Declaration of Helsinki.

Consent for publication

Not applicable.

Data availability statements

The datasets used and/or analysed during the current study are available from the corresponding author on reasonable request.

Competing interests

The authors declare no competing interests.

Funding

This work was supported by the Medical Research Council (G1000508) and Wellcome Trust through the Centre for Future Health at the University of York (award number not available).

REFERENCES

1. Fidler IJ, Kripke ML. The challenge of targeting metastasis. Cancer Metastasis Rev. 2015 Dec;34(4):635–41.
2. Anderson RL, Balasas T, Callaghan J, Coombes RC, Evans J, Hall JA, et al. A framework for the development of effective anti-metastatic agents. Nat Rev Clin Oncol. 2019 Mar;16(3):185–204.
3. Capatina AL, Lagos D, Brackenbury WJ. Targeting Ion Channels for Cancer Treatment: Current Progress and Future Challenges. Rev Physiol Biochem Pharmacol [Internet]. 2020 Sep 1;2020/09/01. Available from: http://dx.doi.org/10.1007/112_2020_46
4. Hille B. Ionic channels of excitable membranes. 2nd ed. Vol. 2nd. Sunderland (Massachusetts): Sinauer Associates Inc.; 1992.

For peer review only

For peer review only - http://bmjopen.bmj.com/site/about/guidelines.xhtml

5. Mantegazza M, Curia G, Biagini G, Ragsdale DS, Avoli M. Voltage-gated sodium channels as therapeutic targets in epilepsy and other neurological disorders. Lancet Neurol. 2010 Apr;9(4):413–24.

6. George AL Jr. Inherited disorders of voltage-gated sodium channels. J Clin Invest. 2005 Aug;115(8):1990–9.

7. Leslie TK, James AD, Zaccagna F, Grist JT, Deen S, Kennerley A, et al. Sodium homeostasis in the tumour microenvironment. Biochim Biophys Acta Rev Cancer. 2019;1872(2):188304.

8. Yang M, Brackenbury WJ. Membrane potential and cancer progression. Front Physiol. 2013;4:185.

9. Djamgoz MBA, Fraser SP, Brackenbury WJ. In Vivo Evidence for Voltage-Gated Sodium Channel Expression in Carcinomas and Potentiation of Metastasis. Cancers [Internet]. 2019;11(11). Available from: https://www.ncbi.nlm.nih.gov/pubmed/31661908

10. Besson P, Driffort V, Bon E, Gradek F, Chevalier S, Roger S. How do voltage-gated sodium channels enhance migration and invasiveness in cancer cells? Biochim Biophys Acta. 2015 Oct;1848(10 Pt B):2493–501.

11. Yang M, James AD, Suman R, Kasprowicz R, Nelson M, O'Toole PJ, et al. Voltage-dependent activation of Rac1 by Nav 1.5 channels promotes cell migration. J Cell Physiol. 2020 Apr;235(4):3950–72.

12. Lopez-Charcas O, Pukkanasut P, Velu SE, Brackenbury WJ, Hales TG, Besson P, et al. Pharmacological and nutritional targeting of voltage-gated sodium channels in the treatment of cancers. iScience [Internet]. 2021 Apr 23 [cited 2021 Mar 22];24(4). Available from: https://cell.com/iscience/retrieve/pii/S2589004221002388?_returnURL=https%3A%2F%2Flinkinghub.elsevier.com%2Fretrieve%2Fpii%2FS2589004221002388%3Fshowall%3Dtrue

13. Nelson M, Yang M, Dowle AA, Thomas JR, Brackenbury WJ. The sodium channel-blocking antiepileptic drug phenytoin inhibits breast tumour growth and metastasis. Mol Cancer. 2015;14(1):13.

14. Driffort V, Gillet L, Bon E, Marionneau-Lambot S, Oullier T, Joulin V, et al. Ranolazine inhibits NaV1.5-mediated breast cancer cell invasiveness and lung colonization. Mol Cancer. 2014;13(1):264.

15. Martin F, Ufodiama C, Watt I, Bland M, Brackenbury WJ. Therapeutic value of voltage-gated sodium channel inhibitors in breast, colorectal and prostate cancer: a systematic review. Front Pharmacol. 2015;6:273.

16. Chamaraux-Tran TN, Mathelin C, Aprahamian M, Joshi GP, Tomasetto C, Diemunsch P, et al. Antitumor Effects of Lidocaine on Human Breast Cancer Cells: An In Vitro and In Vivo Experimental Trial. Anticancer Res. 2018 Jan;38(1):95–105.

17. Johnson MZ, Crowley PD, Foley AG, Xue C, Connolly C, Gallagher HC, et al. Effect of perioperative lidocaine on metastasis after sevoflurane or ketamine-xylazine anaesthesia for breast tumour resection in a murine model. Br J Anaesth. 2018 Jul;121(1):76–85.

18. Freeman J, Crowley PD, Foley AG, Gallagher HC, Iwasaki M, Ma D, et al. Effect of

For peer review only - http://bmjopen.bmj.com/site/about/guidelines.xhtml

Perioperative Lidocaine and Cisplatin on Metastasis in a Murine Model of Breast Cancer Surgery. Anticancer Res. 2018 Oct;38(10):5599–606.

19. Freeman J, Crowley PD, Foley AG, Gallagher HC, Iwasaki M, Ma D, et al. Effect of Perioperative Lidocaine, Propofol and Steroids on Pulmonary Metastasis in a Murine Model of Breast Cancer Surgery. Cancers [Internet]. 2019 May 1;11(5). Available from: http://dx.doi.org/10.3390/cancers11050613

20. Tada M, Imazeki F, Fukai K, Sakamoto A, Arai M, Mikata R, et al. Procaine inhibits the proliferation and DNA methylation in human hepatoma cells. Hepatol Int. 2007 Sep;1(3):355–64.

21. Bugan I, Kucuk S, Karagoz Z, Fraser SP, Kaya H, Dodson A, et al. Anti-metastatic effect of ranolazine in an in vivo rat model of prostate cancer, and expression of voltage-gated sodium channel protein in human prostate. Prostate Cancer Prostatic Dis [Internet]. 2019;2019/03/22. Available from: http://dx.doi.org/10.1038/s41391-019-0128-3

22. Raderer M, Depisch D, Haider K, Kwasny W, Djavanmard M, Scheithauer W. A Phase I/II Study of Quinidine, a Potential Multidrug Resistance-Reversing Agent, in Combination with Pirarubicin in Patients with Advanced Refractory Breast Cancer. Onkologie. 1993;16:450–3.

23. Wheler JJ, Janku F, Falchook GS, Jackson TL, Fu S, Naing A, et al. Phase I study of anti-VEGF monoclonal antibody bevacizumab and histone deacetylase inhibitor valproic acid in patients with advanced cancers. Cancer Chemother Pharmacol. 2014 Mar;73(3):495–501.

24. Takada M, Fujimoto M, Motomura H, Hosomi K. Inverse Association between Sodium Channel-Blocking Antiepileptic Drug Use and Cancer: Data Mining of Spontaneous Reporting and Claims Databases. Int J Med Sci. 2016;13(1):48–59.

25. Walker AJ, Card T, Bates TE, Muir K. Tricyclic antidepressants and the incidence of certain cancers: a study using the GPRD. Br J Cancer. 2011;104(1):193–7.

26. Exadaktylos AK, Buggy DJ, Moriarty DC, Mascha E, Sessler DI. Can anesthetic technique for primary breast cancer surgery affect recurrence or metastasis? Anesthesiology. 2006 Oct;105(4):660–4.

27. Biki B, Mascha E, Moriarty DC, Fitzpatrick JM, Sessler DI, Buggy DJ. Anesthetic technique for radical prostatectomy surgery affects cancer recurrence: a retrospective analysis. Anesthesiology. 2008 Aug;109(2):180–7.

28. Reddy JP, Dawood S, Mitchell M, Debeb BG, Bloom E, Gonzalez-Angulo AM, et al. Antiepileptic drug use improves overall survival in breast cancer patients with brain metastases in the setting of whole brain radiotherapy. Radiother Oncol. 2015 Nov;117(2):308–14.

29. Fairhurst C, Watt I, Martin F, Bland M, Brackenbury WJ. Sodium channel-inhibiting drugs and survival of breast, colon and prostate cancer: a population-based study. Sci Rep. 2015;5:16758.

30. Neligan A, Bell GS, Johnson AL, Goodridge DM, Shorvon SD, Sander JW. The long-term risk of premature mortality in people with epilepsy. Brain. 2011 Feb;134(Pt 2):388–95.

For peer review only - http://bmjopen.bmj.com/site/about/guidelines.xhtml

31. Fairhurst C, Martin F, Watt I, Doran T, Bland M, Brackenbury WJ. Sodium channel-inhibiting drugs and cancer survival: protocol for a cohort study using the CPRD primary care database. BMJ Open. 2016;6(9):e011661.

32. Suissa S. Immortal time bias in pharmaco-epidemiology. Am J Epidemiol. 2008 Feb 15;167(4):492–9.

33. College Station, TX: StataCorp LP. Stata Statistical Software: Release 14. 2015.

34. Simon R, Makuch RW. A non-parametric graphical representation of the relationship between survival and the occurrence of an event: application to responder versus non-responder bias. Stat Med. 1984 Jan;3(1):35–44.

35. Fine JP, Gray RJ. A Proportional Hazards Model for the Subdistribution of a Competing Risk. J Am Stat Assoc. 1999 Jun 1;94(446):496–509.

36. Chubak J, Boudreau DM, Wirtz HS, McKnight B, Weiss NS. Threats to validity of nonrandomized studies of postdiagnosis exposures on cancer recurrence and survival. J Natl Cancer Inst. 2013 Oct 2;105(19):1456–62.

37. Lei M, Wu L, Terrar DA, Huang CL-H. Modernized Classification of Cardiac Antiarrhythmic Drugs. Circulation. 2018 Oct 23;138(17):1879–96.

38. Vaughan Williams EM. Classification of antidysrhythmic drugs. Pharmacol Ther B. 1975;1(1):115–38.

39. Herrett E, Gallagher AM, Bhaskaran K, Forbes H, Mathur R, van Staa T, et al. Data Resource Profile: Clinical Practice Research Datalink (CPRD). Int J Epidemiol. 2015 Jun;44(3):827–36.

40. Jick SS, Kaye JA, Vasilakis-Scaramozza C, Garcia Rodriguez LA, Ruigomez A, Meier CR, et al. Validity of the general practice research database. Pharmacotherapy. 2003 May;23(5):686–9.

41. Clare JJ, Tate SN, Nobbs M, Romanos MA. Voltage-gated sodium channels as therapeutic targets. Drug Discov Today. 2000;5(11):506–20.

42. Suissa S. Immortal time bias in observational studies of drug effects. Pharmacoepidemiol Drug Saf. 2007 Mar;16(3):241–9.

43. Morris EJ, Jordan C, Thomas JD, Cooper M, Brown JM, Thorpe H, et al. Comparison of treatment and outcome information between a clinical trial and the National Cancer Data Repository. Br J Surg. 2011 Feb;98(2):299–307.

44. Keezer MR, Sisodiya SM, Sander JW. Comorbidities of epilepsy: current concepts and future perspectives. Lancet Neurol. 2016 Jan;15(1):106–15.

45. Shmuely S, van der Lende M, Lamberts RJ, Sander JW, Thijs RD. The heart of epilepsy: Current views and future concepts. Seizure. 2017 Jan;44:176–83.

46. Fairhurst C, Watt I, Martin F, Bland M, Brackenbury WJ. Exposure to sodium channel-inhibiting drugs and cancer survival: protocol for a cohort study using the QResearch primary care database. BMJ Open. 2014;4(11):e006604.

47. Yang M, Kozminski DJ, Wold LA, Modak R, Calhoun JD, Isom LL, et al. Therapeutic potential for phenytoin: targeting Na(v)1.5 sodium channels to reduce migration and invasion in metastatic breast cancer. Breast Cancer Res Treat. 2012 Jul;134(2):603–15.

For peer review only - http://bmjopen.bmj.com/site/about/guidelines.xhtml

48. Brackenbury WJ. Voltage-gated sodium channels and metastatic disease. Channels . 2012;6(5):352–61.

49. Fraser SP, Diss JK, Chioni AM, Mycielska ME, Pan H, Yamaci RF, et al. Voltage-gated sodium channel expression and potentiation of human breast cancer metastasis. Clin Cancer Res. 2005;11(15):5381–9.

50. Baptista-Hon DT, Robertson FM, Robertson GB, Owen SJ, Rogers GW, Lydon EL, et al. Potent inhibition by ropivacaine of metastatic colon cancer SW620 cell invasion and NaV1.5 channel function. Br J Anaesth. 2014 Jul;113 Suppl 1:i39–48.

51. Tassara M, Dohner K, Brossart P, Held G, Gotze K, Horst HA, et al. Valproic acid in combination with all-trans retinoic acid and intensive therapy for acute myeloid leukemia in older patients. Blood. 2014;123(26):4027–36.

52. Munster P, Marchion D, Bicaku E, Lacevic M, Kim J, Centeno B, et al. Clinical and biological effects of valproic acid as a histone deacetylase inhibitor on tumor and surrogate tissues: phase I/II trial of valproic acid and epirubicin/FEC. Clin Cancer Res. 2009;15(7):2488–96.

53. Munster P, Marchion D, Bicaku E, Schmitt M, Lee JH, DeConti R, et al. Phase I trial of histone deacetylase inhibition by valproic acid followed by the topoisomerase II inhibitor epirubicin in advanced solid tumors: a clinical and translational study. J Clin Oncol. 2007;25(15):1979–85.

54. Michaelis M, Doerr HW, Cinatl J Jr. Valproic acid as anti-cancer drug. Curr Pharm Des. 2007;13(33):3378–93.

55. Shackleton DP, Westendorp RG, Trenite DG, Vandenbroucke JP. Mortality in patients with epilepsy: 40 years of follow up in a Dutch cohort study. J Neurol Neurosurg Psychiatry. 1999 May;66(5):636–40.

56. Nevalainen O, Raitanen J, Ansakorpi H, Artama M, Isojarvi J, Auvinen A. Long-term mortality risk by cause of death in newly diagnosed patients with epilepsy in Finland: a nationwide register-based study. Eur J Epidemiol. 2013 Dec;28(12):981–90.

57. Gaitatzis A, Carroll K, Majeed A, Sander JW. The epidemiology of the comorbidity of epilepsy in the general population. Epilepsia. 2004 Dec;45(12):1613–22.

58. Singh G, Fletcher O, Bell GS, McLean AE, Sander JW. Cancer mortality amongst people with epilepsy: a study of two cohorts with severe and presumed milder epilepsy. Epilepsy Res. 2009 Feb;83(2-3):190–7.

59. Vecht CJ, Wagner GL, Wilms EB. Interactions between antiepileptic and chemotherapeutic drugs. Lancet Neurol. 2003 Jul;2(7):404–9.

60. Singh G, Driever PH, Sander JW. Cancer risk in people with epilepsy: the role of antiepileptic drugs. Brain. 2005 Jan;128(Pt 1):7–17.

61. Beghi E, Shorvon S. Antiepileptic drugs and the immune system. Epilepsia. 2011 May;52 Suppl 3:40–4.

For peer review only - http://bmjopen.bmj.com/site/about/guidelines.xhtml

Figure legend

Figure 1. Simon-Makuch survival curve for unexposed cancer patients and those ever exposed to VGSC-inhibiting drugs in Scenario 3

For peer review only

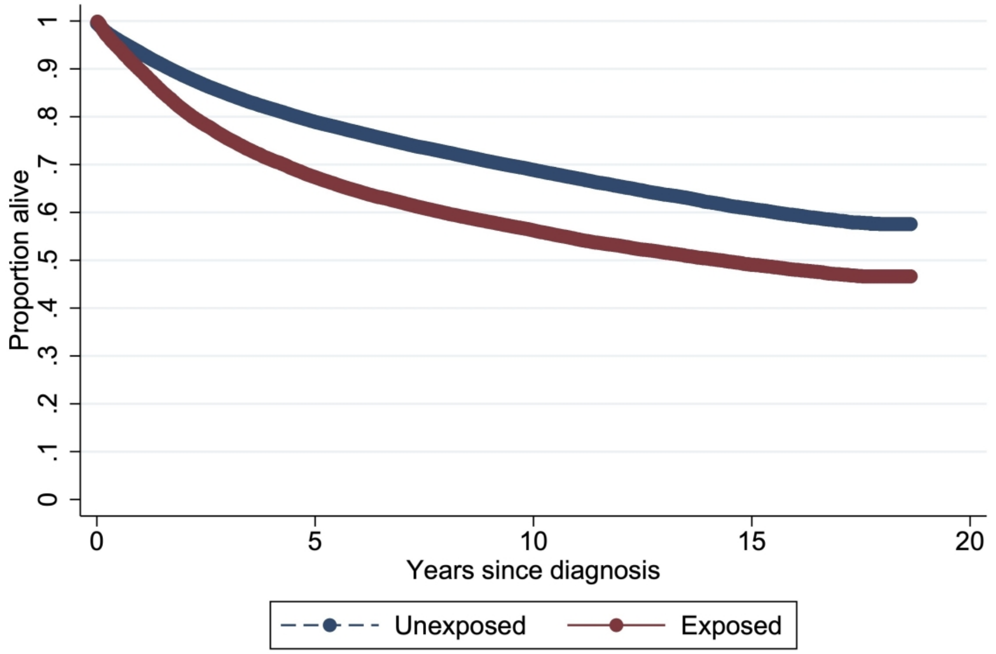

Figure 1

1125x741mm (72 x 72 DPI)

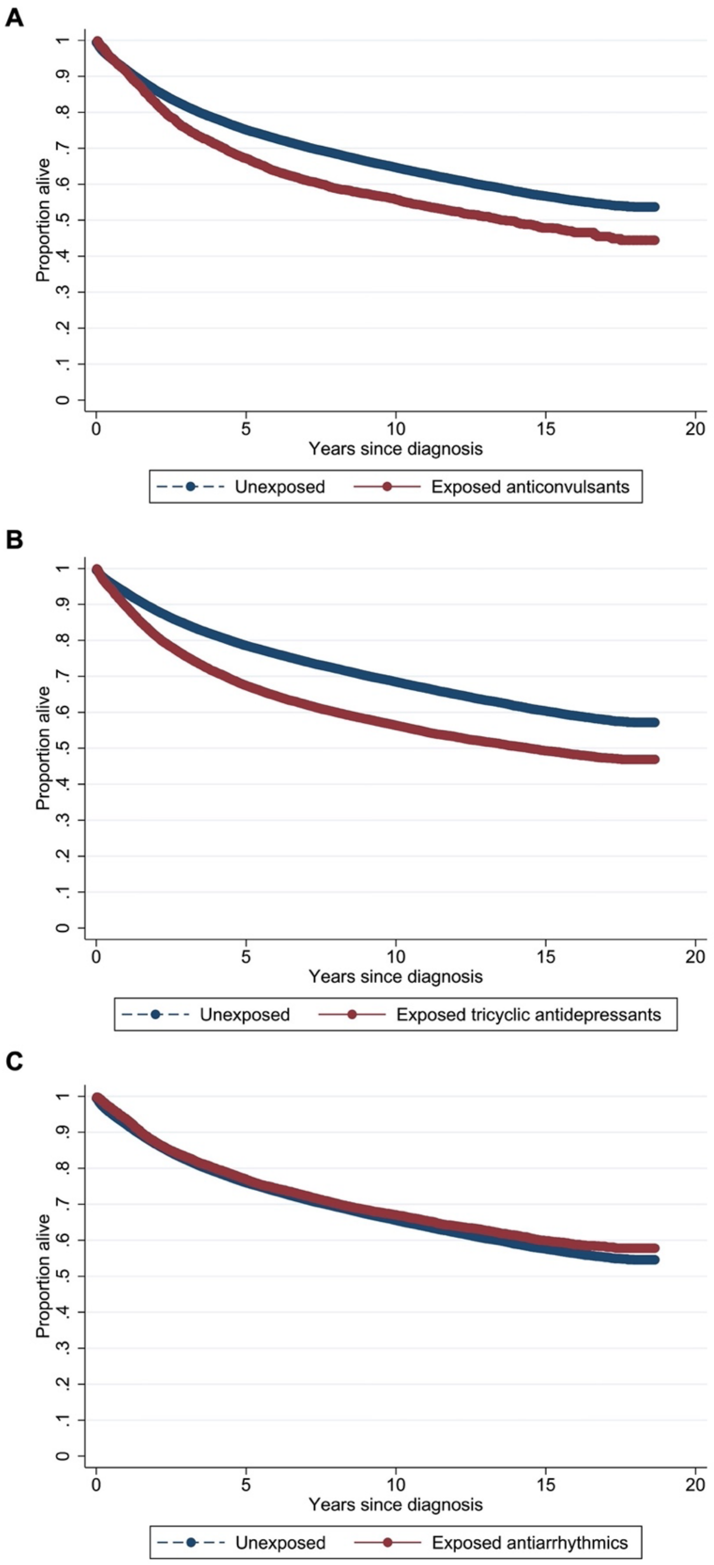

Supplementary Figure 1. Simon-Makuch survival curves for unexposed cancer patients and those ever exposed to VGSC-inhibiting anticonvulsant (A), tricyclic antidepressant (B) and antiarrhythmic (C) drugs in Scenario 3.

For peer review only - http://bmjopen.bmj.com/site/about/guidelines.xhtml

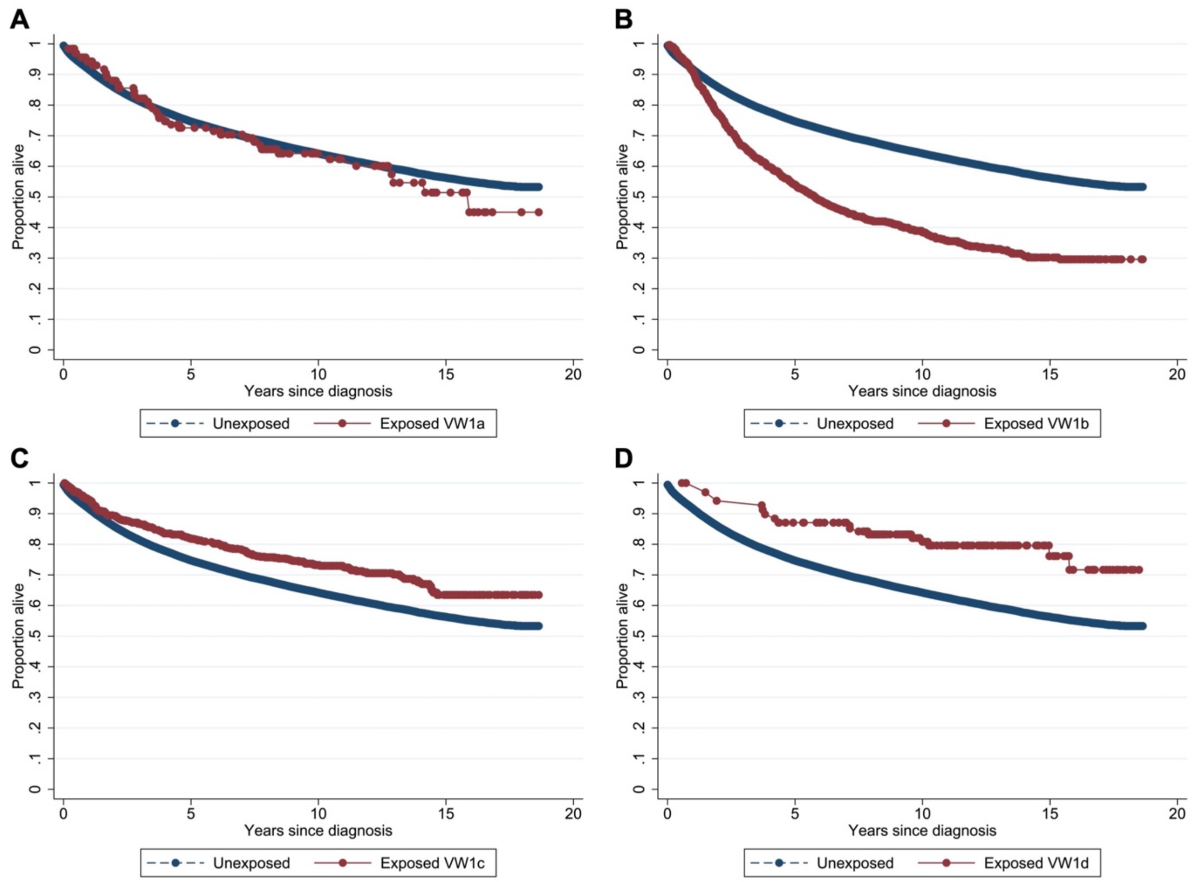

Supplementary Figure 2. Simon-Makuch survival curve for unexposed cancer patients and those ever exposed to Vaughan-Williams Class 1a (A), 1b (B), 1c (C) and 1d (D) drugs in Scenario 3.

For peer review only - http://bmjopen.bmj.com/site/about/guidelines.xhtml

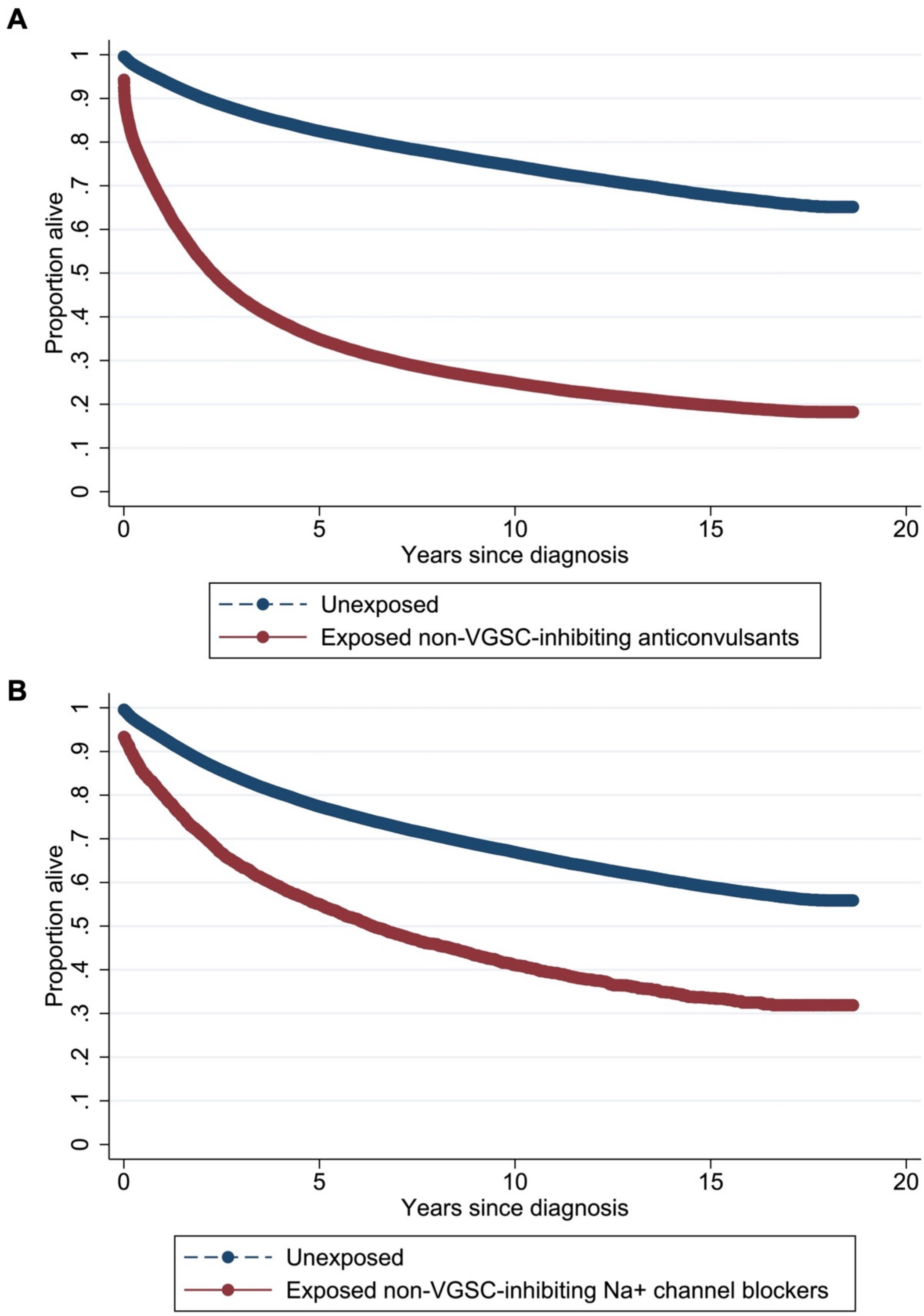

Supplementary Figure 3. Simon-Makuch survival curve for unexposed cancer patients and those ever exposed to non-VGSC-inhibiting anticonvulsants (A) and non-VGSC-inhibiting Na^+ channel blockers (B) in Scenario 3.

Supplementary Table 1. Drug groups and classifications used in this study.

Drug	Classification	Vaughan Williams Classification[a]
**A. VGSC inhibitors**		
Articaine	Amide local anaesthetic	
Bupivacaine	Amide local anaesthetic	
Cinchocaine	Amide local anaesthetic	
Etidocaine	Amide local anaesthetic	
Levobupivacaine	Amide local anaesthetic	
Lidocaine	Amide local anaesthetic	1b
Mepivacaine	Amide local anaesthetic	
Prilocaine	Amide local anaesthetic	
Ropivacaine	Amide local anaesthetic	
Trimecaine	Amide local anaesthetic	
Ranolazine	Antiarrhythmic	1d
Ajmaline	Antiarrhythmic	1a
Amiodarone	Antiarrhythmic	3
Aprindine	Antiarrhythmic	1b
Disopyramide	Antiarrhythmic	1a
Dronedarone	Antiarrhythmic	3
Encainide	Antiarrhythmic	1c
Flecainide	Antiarrhythmic	1c
Mexiletine	Antiarrhythmic	1b
Moricizine/Moricizine hydrochloride	Antiarrhythmic	1c
Pilsicainide	Antiarrhythmic	1c
Procainamide	Antiarrhythmic	1a
Propafenone	Antiarrhythmic	1c
Quinidine	Antiarrhythmic	1a
Tocainide	Antiarrhythmic	1b

For peer review only - http://bmjopen.bmj.com/site/about/guidelines.xhtml

Carvedilol	Antiarrhythmic	2
Labetalol	Antiarrhythmic	2
Oxprenolol	Antiarrhythmic	2
Propranolol	Antiarrhythmic	2
Esmolol	Antiarrhythmic	2
Carbamazepine	Anticonvulsant	
Eslicarbazepine	Anticonvulsant	
Eslicarbazepine acetate	Anticonvulsant	
Ethotoin	Anticonvulsant	
Fosphenytoin	Anticonvulsant	
Lacosamide	Anticonvulsant	
Lamotrigine	Anticonvulsant	
Oxcarbazepine	Anticonvulsant	
Phenytoin	Anticonvulsant	1b
Rufinamide	Anticonvulsant	
Sodium Valproate	Anticonvulsant	
Topiramate	Anticonvulsant	
Valproic acid	Anticonvulsant	
Zonisamide	Anticonvulsant	
Benzocaine	Ester local anaesthetic	
Procaine	Ester local anaesthetic	
Tetracaine	Ester local anaesthetic	
Riluzole	ALS treatment	
Amitriptyline	Tricyclic antidepressant	
Desipramine	Tricyclic antidepressant	
Duloxetine	Tricyclic antidepressant	
Fluoxetine	Tricyclic antidepressant	
Imipramine	Tricyclic antidepressant	
Maprotiline	Tricyclic antidepressant	

For peer review only

Nortriptyline	Tricyclic antidepressant	
**A. Non-VGSC Na^+ channel blocker**		
Amiloride	ENaC inhibitor	
Triamterene	ENaC inhibitor	
**B. Non-VGSC-inhibiting anticonvulsants**		
Perampanel	AMPA receptor non-competitive antagonist	
Stiripentol	Aromatic allylic alcohol	
Phenobarbital	Barbiturate	
Primidone	Barbiturate	
Clobazam	Benzodiazepine	
Clonazepam	Benzodiazepine	
Diazepam	Benzodiazepine	
Lorazepam	Benzodiazepine	
Midazolam	Benzodiazepine	
Ethosuximide	Ca^{2+} channel inhibitor	
Gabapentin	Ca^{2+} channel inhibitor	
Pregabalin	Ca^{2+} channel inhibitor	
Acetazolamide	Carbonic anhydrase inhibitor	
Tiagabine	GABA reuptake inhibitor	
Vigabatrin	GABA reuptake inhibitor	
Brivaracetam	SV2A inhibitor	
Levetiracetam	SV2A inhibitor	

[a]According to (37,38).

For peer review only

For peer review only - http://bmjopen.bmj.com/site/about/guidelines.xhtml

Supplementary Table 2. Characteristics of the participants stratified by exposure status.

	**A VGSC-inhibitor prescription (any at any time exc anaesthetics) (n=53724)**	**No VGSC-inhibitor prescriptions (n=79272)**	**Total (n=132996)**	**p-value**
**Sex, n (%)**				
Male	22531 (41.9)	41402 (52.2)	63933 (48.1)	<0.001
Female	31193 (58.1)	37870 (47.8)	69063 (51.9)	
**Age at diagnosis, years**				
Mean (SD)	65.9 (13.0)	68.0 (13.3)	67.1 (13.2)	<0.001
**Ethnicity, n (%)**				
White	50495 (94.0)	72056 (90.9)	122551 (92.1)	<0.001
Mixed/Multiple ethnic groups	161 (0.3)	299 (0.4)	460 (0.3)	
Asian/Asian British	801 (1.5)	1297 (1.6)	2098 (1.6)	
Black/Black British	733 (1.4)	1597 (2.0)	2330 (1.8)	
Other	190 (0.4)	336 (0.4)	526 (0.4)	

For peer review only - http://bmjopen.bmj.com/site/about/guidelines.xhtml

Not recorded/known	1344 (2.5)	3687 (4.7)	5031 (3.8)	
**Index of Multiple Deprivation, n (%)**				
Mean (SD)	9.2 (5.6)	9.0 (5.5)	9.1 (5.5)	<0.001
**Smoking status, n (%)**				
Heavy smoker	1596 (3.0)	1648 (2.1)	3244 (2.4)	<0.001
Moderate smoker	4503 (8.4)	5259 (6.6)	9762 (7.3)	
Light smoker	1675 (3.1)	2156 (2.7)	3831 (2.9)	
Ex-smoker	16413 (30.6)	23004 (29.0)	39417 (29.6)	
Non-smoker	28676 (53.4)	44072 (55.6)	72748 (54.7)	
Not recorded/known	861 (1.6)	3133 (4.0)	3994 (3.0)	
**Alcohol intake, n (%)**				
Heavy drinker	13656 (25.4)	20565 (25.9)	34221 (25.7)	<0.001
Moderate drinker	3421 (6.4)	5003 (6.3)	8424 (6.3)	
Light drinker	11829 (22.0)	16231 (20.5)	28060 (21.1)	

For peer review only - http://bmjopen.bmj.com/site/about/guidelines.xhtml

Non drinker	9001 (16.8)	10442 (13.2)	19443 (14.6)	
Not recorded/known	15817 (29.4)	27031 (34.1)	42848 (32.2)	
**BMI category, n (%)**				
Overweight/Obese	32166 (59.9)	42432 (53.5)	74598 (56.1)	<0.001
Normal range	15526 (28.9)	24137 (30.4)	39663 (29.8)	
Underweight	2345 (4.4)	3598 (4.5)	5943 (4.5)	
Not recorded/known	3687 (6.9)	9105 (11.5)	12792 (9.6)	
**Physical activity, n (%)**				
Very active	2634 (4.9)	4173 (5.3)	6807 (5.1)	<0.001
Moderately active	18614 (34.6)	25806 (32.6)	44420 (33.4)	
Inactive	7551 (14.1)	8179 (10.3)	15730 (11.8)	
Not recorded/known	24925 (46.4)	41114 (51.9)	66039 (49.7)	
**Type of cancer, n (%)**				
Breast	27106 (50.5)	32422 (40.9)	59528 (44.8)	<0.001

For peer review only - http://bmjopen.bmj.com/site/about/guidelines.xhtml

Bowel	8435 (15.7)	14432 (18.2)	22867 (17.2)	
Prostate	18183 (33.8)	32418 (40.9)	50601 (38.0)	
**Total CCI score**				
Mean (SD)	6.1 (2.8)	5.9 (2.7)	6.0 (2.7)	<0.001
**VGSC-inhibitor indication[a], n (%)**				
Epilepsy	1915 (3.6)	449 (0.6)	2364 (1.8)	<0.001
Cardiac arrhythmia	9646 (18.0)	9791 (12.4)	19437 (14.6)	<0.001
Amyotrophic lateral sclerosis	0 (0.0)	0 (0.0)	0 (0.0)	-
Neuropathic pain/painful neuropathy	9860 (18.4)	7271 (9.2)	17131 (12.9)	<0.001
**≥1 of above, n (%)**	18744 (34.9)	16048 (20.2)	34792 (26.2)	<0.001

[a] not mutually exclusive

VGSC, voltage gated sodium channel; SD, standard deviation; BMI, body mass index; CCI, Charlson Comorbidity Index score.

For peer review only - http://bmjopen.bmj.com/site/about/guidelines.xhtml

Supplementary Table 3. Characteristics of the 'ever' exposed group stratified by timing of exposure relative to their cancer diagnosis.

	**Before only (n=14,157)**	**Before and after (n=17,264)**	**After only (n=22,303)**	**VGSC inhibitor prescription (any at any time excluding local anaesthetics) (n=53,724)**
**Sex, n (%)**				
Male	6049 (42.7)	6325 (36.6)	10157 (45.5)	22531 (41.9)
Female	8108 (57.3)	10939 (63.4)	12146 (54.5)	31193 (58.1)
**Age at diagnosis, years**				
Mean (SD)	68.6 (13.4)	65.7 (12.9)	64.3 (12.5)	65.9 (13.0)
**Ethnicity, n (%)**				
White	13189 (93.2)	16366 (94.8)	20940 (93.9)	50495 (94.0)
Mixed/Multiple ethnic groups	35 (0.2)	47 (0.3)	79 (0.4)	161 (0.3)
Asian/Asian British	172 (1.2)	252 (1.5)	377 (1.7)	801 (1.5)
Black/Black British	169 (1.2)	186 (1.1)	378 (1.7)	733 (1.4)
Other	48 (0.3)	54 (0.3)	88 (0.4)	190 (0.4)
Not recorded/known	544 (3.8)	359 (2.1)	441 (2.0)	1344 (2.5)

For peer review only - http://bmjopen.bmj.com/site/about/guidelines.xhtml
For peer review only

**Index of Multiple Deprivation, n (%)**				
Mean (SD)	9.2 (5.5)	9.5 (5.7)	9.0 (5.5)	9.2 (5.6)
**Smoking status, n (%)**				
Heavy smoker	373 (2.6)	561 (3.2)	662 (3.0)	1596 (3.0)
Moderate smoker	1139 (8.0)	1554 (9.0)	1810 (8.1)	4503 (8.4)
Light smoker	437 (3.1)	520 (3.0)	718 (3.2)	1675 (3.1)
Ex-smoker	4336 (30.6)	5362 (31.1)	6715 (30.1)	16413 (30.6)
Non-smoker	7562 (53.4)	9020 (52.2)	12094 (54.2)	28676 (53.4)
Not recorded/known	310 (2.2)	247 (1.4)	304 (1.4)	861 (1.6)
**Alcohol intake, n (%)**				
Heavy drinker	3467 (24.5)	4030 (23.3)	6159 (27.6)	13656 (25.4)
Moderate drinker	929 (6.6)	1036 (6.0)	1456 (6.5)	3421 (6.4)
Light drinker	3153 (22.3)	3879 (22.5)	4797 (21.5)	11829 (22.0)
Non drinker	2436 (17.2)	3404 (19.7)	3161 (14.2)	9001 (16.8)
Not recorded/known	4172 (29.5)	4915 (28.5)	6730 (30.2)	15817 (29.4)
**BMI category, n (%)**				

For peer review only - http://bmjopen.bmj.com/site/about/guidelines.xhtml

Overweight/Obese	7990 (56.4)	10699 (62.0)	13477 (60.4)	32166 (59.9)
Normal range	4267 (30.1)	4734 (27.4)	6525 (29.3)	15526 (28.9)
Underweight	764 (5.4)	728 (4.2)	853 (3.8)	2345 (4.4)
Not recorded/known	1136 (8.0)	1103 (6.4)	1448 (6.5)	3687 (6.9)
**Physical activity, n (%)**				
Very active	615 (4.3)	752 (4.4)	1267 (5.7)	2634 (4.9)
Moderately active	4497 (31.8)	5896 (34.2)	8221 (36.9)	18614 (34.6)
Inactive	1785 (12.6)	2816 (16.3)	2950 (13.2)	7551 (14.1)
Not recorded/known	7260 (51.3)	7800 (45.2)	9865 (44.2)	24925 (46.4)
**Type of cancer, n (%)**				
Breast	6766 (47.8)	9642 (55.9)	10698 (48.0)	27106 (50.5)
Bowel	2531 (17.9)	2472 (14.3)	3432 (15.4)	8435 (15.7)
Prostate	4860 (34.3)	5150 (29.8)	8173 (36.6)	18183 (33.8)
**Total CCI score**				
Mean (SD)	6.3 (2.8)	6.1 (2.8)	5.9 (2.8)	6.1 (2.8)
**Diagnosis of a VGSC inhibitor indication[a], n (%)**				

For peer review only - http://bmjopen.bmj.com/site/about/guidelines.xhtml

Epilepsy	202 (1.4)	1271 (7.4)	442 (2.0)	1915 (3.6)
Cardiac arrhythmia	2611 (18.4)	3362 (19.5)	3673 (16.5)	9646 (18.0)
Amyotrophic lateral sclerosis	0 (0.0)	0 (0.0)	0 (0.0)	0 (0.0)
Neuropathic pain/painful neuropathy	2126 (15.0)	3790 (22.0)	3944 (17.7)	9860 (18.4)
**≥1 of above, n (%)**	4400 (31.1)	7199 (41.7)	7145 (32.0)	18744 (34.9)
**Died, n (%)**	7800 (55.1)	8119 (47.0)	10109 (45.3)	26028 (48.4)
**Most common prescription 1[a]**				
Tricyclic antidepressant	9312 (65.8)	11495 (66.6)	17793 (79.8)	38600 (71.8)
Antiarrhythmic	4023 (28.4)	3828 (22.2)	3181 (14.3)	11032 (20.5)
Anticonvulsant	822 (5.8)	1935 (11.2)	1305 (5.9)	4062 (7.6)
Treatment for ALS	0 (0.0)	6 (0.0)	24 (0.1)	30 (0.1)
**Most common prescription 2[b]**				
Tricyclic antidepressant	7887 (55.7)	10831 (62.7)	15187 (68.1)	33905 (63.1)
Antiarrhythmic	3604 (25.5)	3726 (21.6)	2881 (12.9)	10211 (19.0)
Amide local anaesthetic	1905 (13.5)	782 (4.5)	2951 (13.2)	5638 (10.5)

For peer review only

Anticonvulsant	708 (5.0)	1907 (11.0)	1191 (5.3)	3806 (7.1)
Ester local anaesthetic	53 (0.4)	12 (0.1)	71 (0.3)	136 (0.3)
Treatment for ALS	0 (0.0)	6 (0.0)	22 (0.1)	28 (0.1)
**Length of exposure (days)**				
Mean (SD)	2095.1 (2220.1)	4889.9 (2473.2)	1912.7 (2077.5)	2917.5 (2627.5)
< 6 months, n (%)	3239 (22.9)	40 (0.2)	4589 (20.6)	7868 (14.6)
≥ 6 months, n (%)	10918 (77.1)	17224 (99.8)	17714 (79.4)	45856 (85.4)
**Recent exposure[c], n (%)**	1017 (7.2)	8580 (49.7)	0 (0.0)	9597 (17.9)

[a] excluding local anaesthetics.

[b] including local anaesthetics.

[c] ≥2 prescriptions relating to one of the VGSC-inhibiting drugs within 2 years before the date of the cancer diagnosis, including at least one within 6 months before.

For peer review only

For peer review only - http://bmjopen.bmj.com/site/about/guidelines.xhtml

Supplementary Table 4. Deaths stratified by exposure to non-VGSC-inhibiting anticonvulsants and non-VGSC-inhibiting Na^+ channel blockers.

	**A VGSC-inhibitor prescription (any other than Amide or Ester local anaesthetics at any time) (n=53724)**	**No VGSC-inhibitor prescriptions (except Amide or Ester local anaesthetics) (n=79272)**	**A non-VGSC-inhibiting anticonvulsant prescription (any at any time) (n=46017)**	**No exposure to a non-VGSC-inhibiting anticonvulsant prescription (n=86979)**	**Non-VGSC-inhibiting Na^+ channel blocker prescription (any at any time) (n=9256)**	**No exposure to a non-VGSC-inhibiting Na^+ channel blocker prescription (n=123740)**
Died (any cause)	26028 (48.4)	40932 (51.6)	25284 (54.9)	41676 (47.9)	6969 (75.3)	59991 (48.5)
Died with any cancer as underlying cause	15933 (29.7)	26104 (32.9)	17987 (39.1)	24050 (27.7)	3601 (38.9)	38436 (31.1)
Died with any cancer as contributory cause	18598 (34.6)	30492 (38.5)	20003 (43.5)	29087 (33.4)	4540 (49.0)	44550 (36.0)
Died with index cancer as underlying cause	12282 (22.9)	20443 (25.8)	13925 (30.3)	18800 (21.6)	2842 (30.7)	29883 (24.1)

For peer review only - http://bmjopen.bmj.com/site/about/guidelines.xhtml

Died with index cancer as contributory cause	15256 (28.4)	25482 (32.1)	16451 (35.7)	24287 (27.9)	3834 (41.4)	36904 (29.8)

For peer review only

Supplementary Table 5. Estimates of the relationship between exposure to non-VGSC-inhibiting drugs, subdivided by type, and cancer-specific mortality (empty cells indicate that analysis was not permitted due to low numbers).

**Non-VGSC-inhibiting anticonvulsants drug groups**	**Exposed* (n=46017), n (%)**	**HR (95% CI) p-value Scenario 1**	**HR (95% CI) p-value Scenario 2**	**HR (95% CI) p-value Scenario 3**
Ever use				
AMPA receptor non-competitive antagonist	4 (0.1)	-	-	-
Aromatic allylic alcohol	0 (0.0)	-	-	-
Barbiturate	469 (1.0)	1.39 (1.19, 1.63) p<0.001	1.28 (1.07, 1.52) p=0.01	1.42 (1.19, 1.69) p<0.001
Benzodiazepine	37696 (81.9)	3.01 (2.95, 3.07) p<0.001	3.73 (3.65, 3.81) p<0.001	4.91 (4.80, 5.02) p<0.001
Calcium channel inhibitor	11643 (21.7)	2.19 (2.12, 2.26) p<0.001	2.27 (2.19, 2.34) p<0.001	2.80 (2.71, 2.90) p<0.001
Carbonic anhydrase inhibitor	14274 (31.0)	0.92 (0.79, 1.06) p=0.25	0.93 (0.76, 1.13) p=0.46	1.25 (1.02, 1.53) p=0.03
GABA reuptake inhibitor	23 (0.1)	-	-	-
SV2A inhibitor	620 (1.4)	2.98 (2.63, 3.38) p<0.001	3.03 (2.67, 3.44) p<0.001	3.75 (3.30, 4.25) p<0.001
Recent use				

For peer review only - http://bmjopen.bmj.com/site/about/guidelines.xhtml

AMPA receptor non-competitive antagonist	0 (0.0)	-	-	-
Aromatic allylic alcohol	0 (0.0)	-	-	-
Barbiturate	208 (0.5)	1.44 (1.16, 1.78) p<0.001	1.37 (1.10, 1.71) p<0.001	1.46 (1.18, 1.82) p<0.001
Benzodiazepine	2686 (5.8)	1.46 (1.37, 1.56) p<0.001	1.34 (1.25, 1.44) p<0.001	1.51 (1.41, 1.62) p<0.001
Calcium channel inhibitor	902 (2.0)	1.12 (0.99, 1.26) p=0.08	1.06 (0.93, 1.21) p=0.38	1.20 (1.05, 1.37) p=0.01
Carbonic anhydrase inhibitor	43 (0.1)	-	-	-
GABA reuptake inhibitor	0 (0.0)	-	-	-
SV2A inhibitor	54 (0.1)	-	-	-
Most common VGSC-inhibitor prescription				
AMPA receptor non-competitive antagonist	0 (0.0)	-	-	-
Aromatic allylic alcohol	0 (0.0)	-	-	-
Barbiturate	348 (0.8)	1.43 (1.20, 1.71) p<0.001	1.30 (1.07, 1.59) p=0.01	1.40 (1.15, 1.71) p<0.001

For peer review only - http://bmjopen.bmj.com/site/about/guidelines.xhtml

Benzodiazepine	32563 (70.8)	3.06 (2.99, 3.12) p<0.001	4.02 (3.93, 4.11) p<0.001	5.11 (4.99, 5.23) p<0.001
Calcium channel inhibitor	12061 (26.2)	2.22 (2.15, 2.30) p<0.001	2.33 (2.24, 2.41) p<0.001	2.80 (2.70, 2.90) p<0.001
Carbonic anhydrase inhibitor	561 (1.2)	0.92 (0.78, 1.08) p=0.31	0.92 (0.72, 1.18) p=0.52	1.22 (0.96, 1.56) p=0.11
GABA reuptake inhibitor	7 (0.0)	-	-	-
SV2A inhibitor	477 (1.0)	3.16 (2.73, 3.65) p<0.001	3.23 (2.79, 3.74) p<0.001	4.00 (3.45, 4.63) p<0.001

*Figures in this column relate to the number of patients recorded as having at least some follow-up time considered as exposed to the drug group of interest in Scenario 1 for each definition (ever use, recent use, most common), as a percentage of the whole 'ever' exposed group. The number of patients with any person-time of follow-up considered as exposed for each drug group will be lower in Scenario 2, and fewer still in Scenario 3.

For peer review only - http://bmjopen.bmj.com/site/about/guidelines.xhtml

Reporting checklist for cohort study.

Based on the STROBE cohort guidelines.

Instructions to authors

Complete this checklist by entering the page numbers from your manuscript where readers will find each of the items listed below.

Your article may not currently address all the items on the checklist. Please modify your text to include the missing information. If you are certain that an item does not apply, please write "n/a" and provide a short explanation.

Upload your completed checklist as an extra file when you submit to a journal.

In your methods section, say that you used the STROBE cohortreporting guidelines, and cite them as:

von Elm E, Altman DG, Egger M, Pocock SJ, Gotzsche PC, Vandenbroucke JP. The Strengthening the Reporting of Observational Studies in Epidemiology (STROBE) Statement: guidelines for reporting observational studies.

		Reporting Item	Page Number
**Title and abstract**			
Title	#1a	Indicate the study’s design with a commonly used term in the title or the abstract	1
Abstract	#1b	Provide in the abstract an informative and balanced summary of what was done and what was found	2
**Introduction**			
Background / rationale	#2	Explain the scientific background and rationale for the investigation being reported	4
Objectives	#3	State specific objectives, including any prespecified hypotheses	4
**Methods**			
Study design	#4	Present key elements of study design early in the paper	5
Setting	#5	Describe the setting, locations, and relevant dates, including periods of	5

For peer review only

		recruitment, exposure, follow-up, and data collection	
Eligibility criteria	#6a	Give the eligibility criteria, and the sources and methods of selection of participants. Describe methods of follow-up.	5
Eligibility criteria	#6b	For matched studies, give matching criteria and number of exposed and unexposed	5
Variables	#7	Clearly define all outcomes, exposures, predictors, potential confounders, and effect modifiers. Give diagnostic criteria, if applicable	7
Data sources / measurement	#8	For each variable of interest give sources of data and details of methods of assessment (measurement). Describe comparability of assessment methods if there is more than one group. Give information separately for for exposed and unexposed groups if applicable.	7
Bias	#9	Describe any efforts to address potential sources of bias	6
Study size	#10	Explain how the study size was arrived at	5
Quantitative variables	#11	Explain how quantitative variables were handled in the analyses. If applicable, describe which groupings were chosen, and why	7
Statistical methods	#12a	Describe all statistical methods, including those used to control for confounding	

5

Statistical methods	#12b	Describe any methods used to examine subgroups and interactions	8
Statistical methods	#12c	Explain how missing data were addressed	8
Statistical methods	#12d	If applicable, explain how loss to follow-up was addressed	6
Statistical methods	#12e	Describe any sensitivity analyses	

8

Results

Participants	#13a	Report numbers of individuals at each stage of study—eg numbers potentially eligible, examined for eligibility, confirmed eligible,	10

For peer review only

		included in the study, completing follow-up, and analysed. Give information separately for for exposed and unexposed groups if applicable.	
Participants	#13b	Give reasons for non-participation at each stage	10
Participants	#13c	Consider use of a flow diagram	
10			
Descriptive data	#14a	Give characteristics of study participants (eg demographic, clinical, social) and information on exposures and potential confounders. Give information separately for exposed and unexposed groups if applicable.	10
Descriptive data	#14b	Indicate number of participants with missing data for each variable of interest	
10			
Descriptive data	#14c	Summarise follow-up time (eg, average and total amount)	
11			
Outcome data	#15	Report numbers of outcome events or summary measures over time. Give information separately for exposed and unexposed groups if applicable.	
11			
Main results	#16a	Give unadjusted estimates and, if applicable, confounder-adjusted estimates and their precision (eg, 95% confidence interval). Make clear which confounders were adjusted for and why they were included	12
Main results	#16b	Report category boundaries when continuous variables were categorized	12
Main results	#16c	If relevant, consider translating estimates of relative risk into absolute risk for a meaningful time period	
12			
Other analyses	#17	Report other analyses done—eg analyses of subgroups and interactions, and sensitivity analyses	14
**Discussion**			
Key results	#18	Summarise key results with reference to study objectives	23

For peer review only

Limitations	#19	Discuss limitations of the study, taking into account sources of potential bias or imprecision. Discuss both direction and magnitude of any potential bias.	24
Interpretation	#20	Give a cautious overall interpretation considering objectives, limitations, multiplicity of analyses, results from similar studies, and other relevant evidence.	25
Generalisability	#21	Discuss the generalisability (external validity) of the study results	25
**Other Information**			
Funding	#22	Give the source of funding and the role of the funders for the present study and, if applicable, for the original study on which the present article is based	27

The STROBE checklist is distributed under the terms of the Creative Commons Attribution License CC-BY. This checklist was completed on 26. April 2022 using https://www.goodreports.org/, a tool made by the EQUATOR Network in collaboration with Penelope.ai

For peer review only
